# Fe‐S Protein FDX1 Triggers Tumor‐Intrinsic Innate Immunity via Mitochondrial Nucleic Acids Release to Orchestrate Ferroptosis in CCRCC

**DOI:** 10.1002/advs.202518323

**Published:** 2025-11-07

**Authors:** Xing Huang, Shaoqing Yu, Wenjie Wei, Wen Tao, Tianwei Cai, Lequan Wen, Jiali Ye, Chi Zhang, Huayi Feng, Senming Cao, Baojun Wang, Xin Ma, Yan Huang, Xu Zhang

**Affiliations:** ^1^ Senior Department of Urology Chinese PLA General Hospital Beijing 100039 China; ^2^ Medical School of PLA Beijing 100853 China; ^3^ School of Medicine Nankai University Tianjin 300071 China

**Keywords:** ccRCC, dsRNA, FDX1, ferroptosis, innate immunity, mitophagy, mtDNA

## Abstract

Activation of cytosolic nucleic acid‐sensing pathways represents a promising strategy to convert immunologically “cold” tumors into inflamed ones. Iron–sulfur (Fe–S) enzymes are critical regulators of innate immunity and nucleic acid sensing, yet their roles in cancer remain poorly defined. Here, ferredoxin‐1 (FDX1), a mitochondrial Fe–S protein frequently downregulated in clear cell renal cell carcinoma (ccRCC), is identified as a dual regulator of ferroptosis and antitumor immunity. FDX1 overexpression triggers mitochondrial permeability transition pore opening, leading to cytosolic release of mitochondrial DNA (mtDNA) and double‐stranded RNA (mt‐dsRNA). This reveals an independent function of FDX1 as a tumor‐intrinsic immunity activator linked to mitochondrial stress signaling. These damage‐associated molecular patterns (DAMPs) engage cytosolic nucleic acid sensors—specifically cGAS and RIG‐I/MDA5—triggering TBK1 phosphorylation and a robust type I interferon response that occurs prior to overt ferroptosis. This innate immune cascade reshapes the tumor microenvironment by enhancing MHC I/II antigen presentation, recruiting CD8+ T cells, and suppressing tumor growth and metastasis in orthotopic syngeneic models. These findings uncover a previously unrecognized antitumor axis through which FDX1 synergizes with mitochondrial nucleic acid release with ferroptosis to promote immunogenic inflammation and T cell infiltration in ccRCC, offering novel therapeutic opportunities targeting mitochondrial‐immune crosstalk.

## Graphical abstract

Restoration of the mitochondrial Fe–S protein FDX1 suppresses clear cell renal cell carcinoma by coupling ferroptosis with tumor‐intrinsic innate immune activation. FDX1 induces mitochondrial dysfunction and mtDNA/mt‐dsRNA release, activating cGAS–STING and RIG‐I/MDA5–MAVS signaling to elicit type I interferon responses, thereby promoting antigen presentation, CD8⁺ T‐cell infiltration, and an inflamed tumor microenvironment.

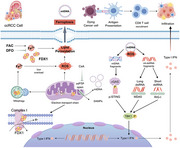

## 1 Introduction

Clear cell renal cell carcinoma (ccRCC), the most prevalent form of kidney cancer, represents a malignancy with distinctive metabolic reprogramming characterized by pseudohypoxia, lipid accumulation, and mitochondrial dysfunction.^[^ ^1^ ^]^ While immune checkpoint inhibitors (ICIs) have revolutionized ccRCC treatment,^[^ ^2^ ^]^ their efficacy remains limited by primary and acquired resistance, often attributed to the immunosuppressive tumor microenvironment (TME).^[^ ^3^, ^4^ ^]^ Recent studies have highlighted ferroptosis–an iron‐dependent form of regulated cell death driven by lipid peroxidation–as a promising therapeutic vulnerability in ccRCC.^[^ ^5^ ^]^ However, tumors frequently develop resistance to ferroptosis induction through various mechanisms,^[^ ^6^ ^]^ underscoring the need for novel approaches to simultaneously trigger tumor cell death and enhance antitumor immunity.

A critical gap in current understanding lies in the identification of molecular integrators capable of coordinating the diverse functions of mitochondria in programmed cell death.^[^ ^7^ ^]^ and the activation of innate immunity,^[^ ^8^, ^9^ ^]^ which could offer therapeutic opportunities in ccRCC. Emerging evidence suggests that mitochondrial damage and subsequent release of mitochondrial DNA (mtDNA) and double‐stranded RNA (mt‐dsRNA) can activate innate immune sensors such as the cyclic GMP‐AMP Synthase‐Stimulator of Interferon Genes (cGAS‐STING) and Retinoic acid‐Inducible Gene‐I/Melanoma Differentiation‐Associated protein 5 (RIG‐I/MDA5) pathways.^[^ ^10^, ^11^ ^]^ While these pathways have been implicated in antiviral responses, their role in tumor immunogenicity and potential synergy with cell death remains poorly understood in ccRCC. Notably, ferredoxin‐1 (FDX1), a mitochondrial iron‐sulfur protein essential for electron donation to Complex I of the electron transport chain (ETC),^[^ ^12^ ^]^ is frequently downregulated in ccRCC and correlates with poor prognosis in our previous cohort.^[^ ^13^ ^]^ Central to renal cancer pathogenesis is mitochondrial dysfunction, characterized particularly by impaired ETC activity and enzymatic dysfunction.^[^ ^14^, ^15^, ^16^ ^]^ Besides, iron‐sulfur (Fe‐S) proteins are primitively active in DNA replication and tRNA modification, and modulation of the antiviral innate immune response.^[^ ^17^, ^18^ ^]^ However, whether and how FDX1 mechanistically orchestrates mitochondrial function in cell fate decision and immunological regulation remains unexplored.

Here, we identify FDX1 as a significant regulator that bridges mitochondrial hemostasis, ferroptosis, and innate immunity in ccRCC. We demonstrate that FDX1 restoration triggers mitochondrial permeability transition pore (mPTP) opening, leading to cytosolic release of mtDNA and mt‐dsRNA. These damage‐associated molecular patterns (DAMPs) activate both cGAS‐STING and RIG‐I/MDA5 pathways, driving a robust type I interferon response that precedes and synergizes with ferroptosis. Importantly, this immune activation remodels the TME by enhancing antigen presentation and recruiting CD8+ T cells, while ferroptosis inhibitors fail to block immune signaling, revealing FDX1's unique role as a molecular activator in tumor intrinsic innate immunity.

These findings fundamentally advance our understanding of mitochondrial‐immune crosstalk in ccRCC and position FDX1 as a promising therapeutic target. By revealing how mitochondrial nucleic acid release can bridge ferroptosis and antitumor immunity, our work opens new avenues for treating ccRCC with metabolic vulnerabilities. The discovery that FDX1 coordinates a feedforward loop of mitochondrial damage, immune activation, and cell death provides a conceptual framework for developing novel combination therapies that simultaneously target multiple cancer hallmarks.

## 2 Results

### 2.1 FDX1 Expression is Regulated by Iron Availability and Selectively Induces Cell Death in ccRCC

**Figure 1:**
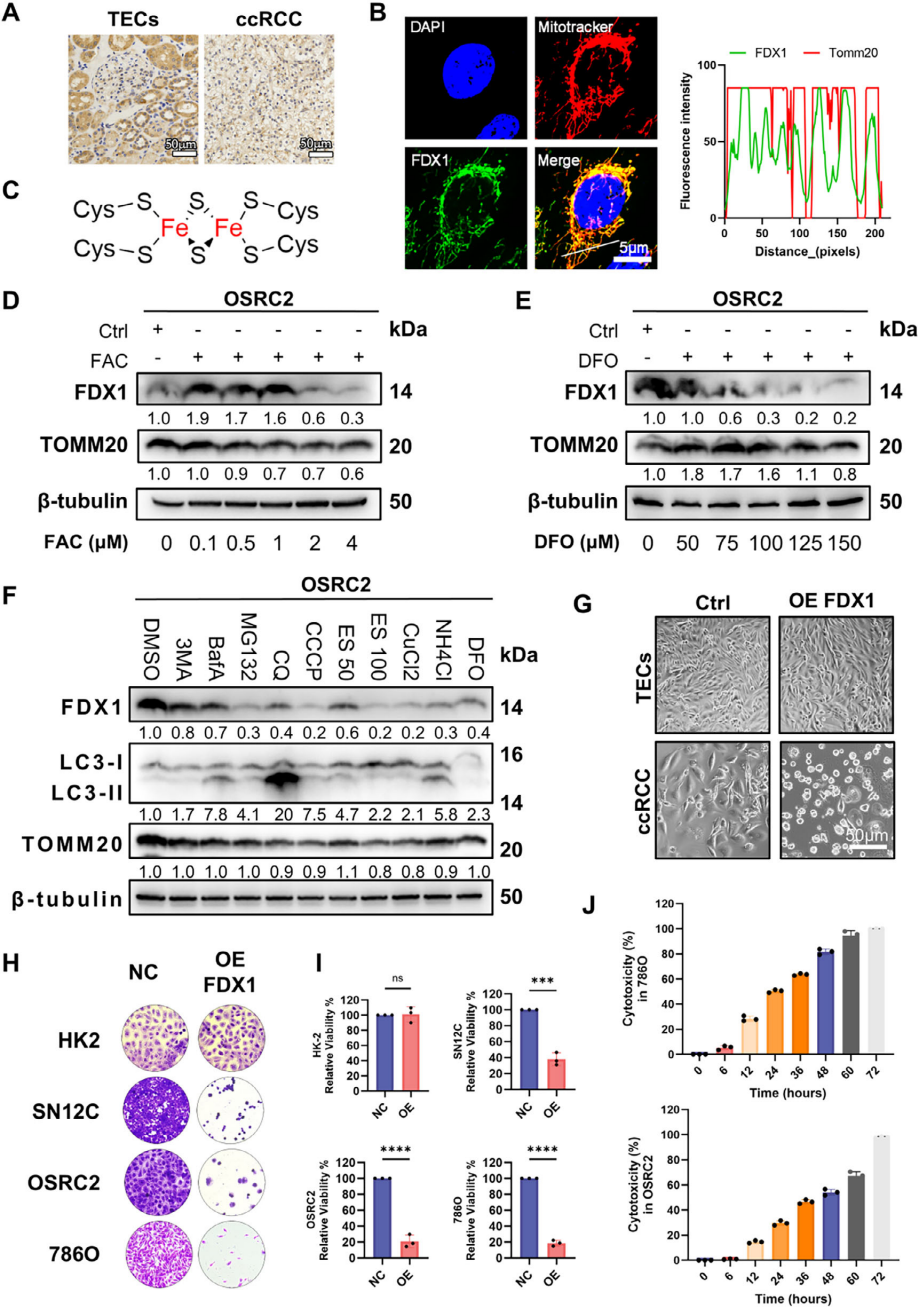
FDX1 expression is iron‐responsive, and its overexpression reduces ccRCC cell viability. A) Immunohistochemical staining of FDX1 in paired patient‐derived TECs and ccRCC tissues reveals markedly reduced expression in tumors. Scale bars: 50 µm. B) Subcellular localization of FDX1 (green, Alexa Fluor 488) in OSRC2 cells transfected with an FDX1 overexpression construct. Mitochondria were stained with MitoTracker Deep Red (red) and nuclei with DAPI (blue). Right panel: fluorescence intensity profile along the indicated line confirms mitochondrial localization. Scale bar: 5 µm. C) Chemical structure of human FDX1. D and E) Immunoblot analysis of FDX1 and mitochondrial marker TOMM20 in OSRC2 cells treated for 24 h with either FAC 0–4 µM or DFO 50–150 µM. FDX1 expression increased with iron supplementation and decreased with iron chelation. β‐Tubulin served as a loading control. F) Immunoblot analysis of FDX1 expression following 12 h treatment with compounds affecting iron homeostasis, autophagy, or mitochondrial stress, including 3‐MA (5 mM), BafA1 (100 nM), MG‐132 (5 µM), CQ (50 µM), CCCP (20 µM), ES (100 nM), CuCl_2_ (150 µM), and DFO (100 µM). β‐Tubulin was used as a loading control. G) Brightfield images of primary cultured TECs and ccRCC cells transfected with control or FDX1 overexpression vector for 48 h. FDX1‐overexpressing tumor cells exhibit reduced cell density. Scale bars: 50 µm. H) Crystal violet staining of normal renal epithelial cells (HK‐2) and ccRCC cell lines (SN12C, OSRC2, 786‐O) 48 h after transfection with control or FDX1 overexpression construct. I) Quantification of relative cell viability based on crystal violet absorbance in cells treated as in (H) (n = 3). J) LDH release assay measuring cytotoxicity in OSRC2 and 786‐O cells at the indicated time points post‐FDX1 transfection (n = 3). Data are presented as mean ± SD; ns, not significant; ^***^ *p*< 0.001; ^****^ *p*< 0.0001, Student's *t*‐test (I).

Analysis of tumor transcriptomic datasets and tissue microarray with 5‐year follow‐up revealed that FDX1 expression is significantly reduced in clear cell renal cell carcinoma and is correlated with poor clinical outcomes in our previous cohorts,^[^ ^13^ ^]^ suggesting a potential tumor‐suppressive role. Immunohistochemical staining confirmed that the cytoplasmic FDX1 protein level was markedly lower in ccRCC tissues than in adjacent normal renal epithelial tissues (**Figure** **1**A). Immunofluorescence further demonstrated that FDX1 colocalizes with mitochondria in OSRC2 cells (Figure 1B), which is consistent with its proposed role in mitochondrial regulation. Importantly, mitochondrial iron–sulfur clusters utilize iron to assemble cofactors essential for oxidoreductase activity.^[^ ^19^ ^]^ Given FDX1's requirement for labile iron and reduced cysteine in Fe–S cluster biogenesis (Figure 1C), we hypothesized that its expression is regulated by intracellular iron levels. Treatment with ferric ammonium citrate (FAC) at subcytotoxic doses (0–1 µM) for 24 h significantly upregulated FDX1 protein expression, whereas higher concentrations (2–4 µM) induced toxicity and altered expression patterns (Figure 1D). Conversely, the iron chelator deferoxamine (DFO, 50–150 µM) suppressed FDX1 expression in a dose‐dependent manner (Figure 1E). Changes in translocase of outer mitochondrial membrane 20 (TOMM20) levels under these conditions suggest that iron availability also affects mitochondrial mass or biogenesis. Inhibitors of lysosomal function—critical for intracellular iron mobilization—such as carbonyl cyanide m‐chlorophenyl hydrazone (CCCP),^[^ ^20^ ^]^ NH4Cl, and bafilomycin A1 (Baf‐A1),^[^ ^21^, ^22^ ^]^ as well as other modulators, including 3‐Methyladenine (3‐MA),^[^ ^23^ ^]^ chloroquine (CQ),^[^ ^24^ ^]^ MG‐132,^[^ ^25^ ^]^ CuCl2, and elesclomol (ES),^[^ ^26^ ^]^ led to decreased FDX1 expression within 12 h of treatment (Figure 1F), collectively confirming that FDX1 expression is tightly regulated by intracellular iron availability through multiple pathways.

To investigate the functional role of FDX1, we titrated FDX1‐overexpressing ccRCC cells and normal renal tubular epithelial cells (TECs) with various concentrations of plasmid DNA (Figure S1A,B, Supporting Information). Medium‐dose (3 µg/2 mL) transfection induced peak FDX1 expression at 36–48 h posttransfection (Figure S1C,D, Supporting Information), with levels comparable to those of HK2 and TECs (Figure S1E,F, Supporting Information), thereby minimizing nonspecific overexpression artifacts. Under these optimized conditions, FDX1 overexpression induced marked cell death in ccRCC lines (OSRC2, 786‐O, and SN12C) but spared normal epithelial cell HK2 and TECs (Figure 1G–I). Cellular lactate dehydrogenase (LDH) release measurements confirmed this selective cytotoxicity after 12 h and suggested a form of cell death with loss of membrane integrity (Figure 1J).

Taken together, these findings establish that FDX1 is a mitochondrion‐localized, iron‐responsive protein whose reactivation preferentially induces death in renal cancer cells.

### 2.2 FDX1 Overexpression Induces Ferroptosis via ROS Accumulation, Lipid Peroxidation, and Mitochondrial Dysfunction

**Figure 2:**
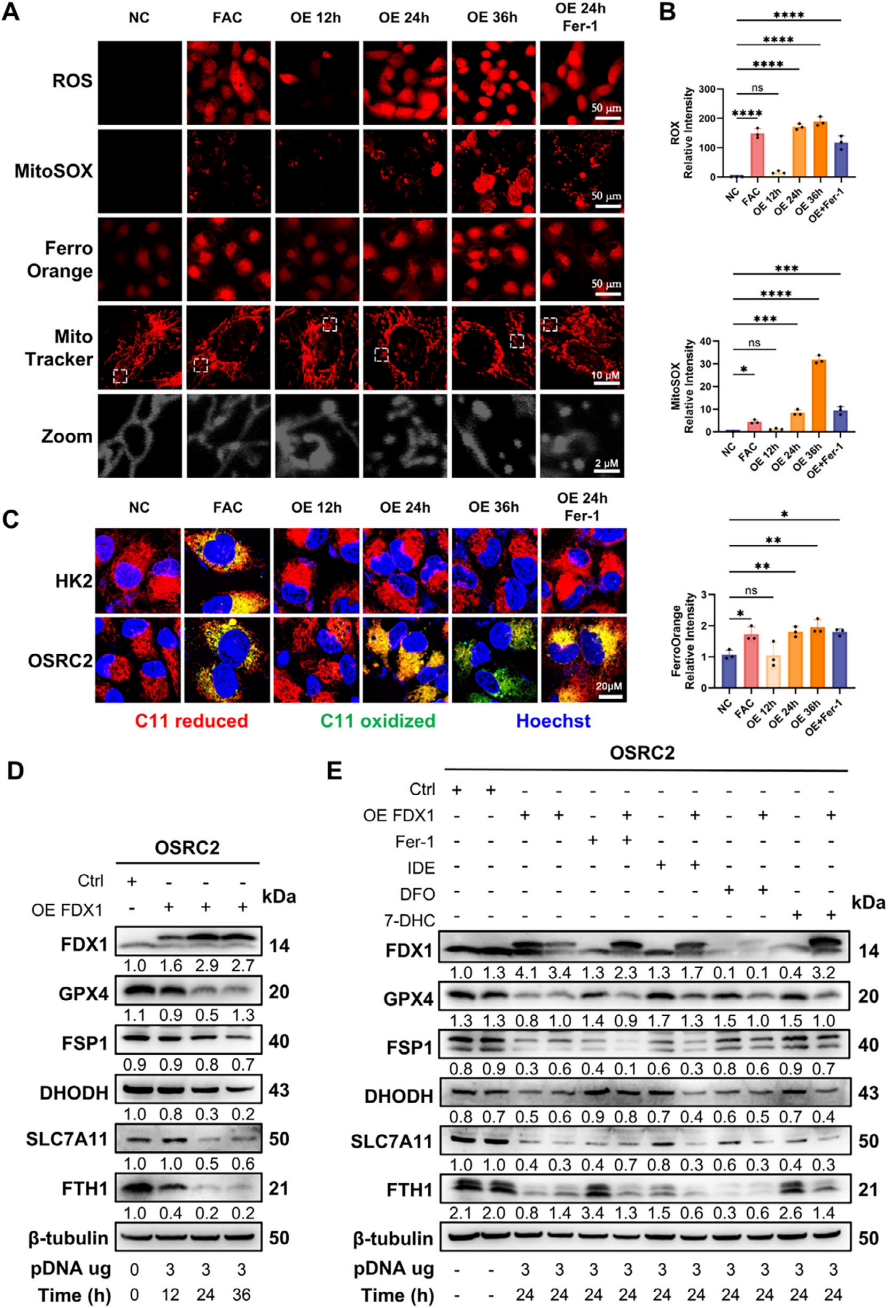
FDX1 promotes lipid peroxidation, which could not be rescued by ferroptosis inhibitors. A) Detection of intracellular ROS (DCFDA Red), mitochondrial superoxide (MitoSOX Red), and labile Fe^2^⁺ (FerroOrange) in OSRC2 cells transfected with control or FDX1 overexpression vector. Positive control: 0.5 µM FAC; rescue: 10 µM Fer‐1. Images captured under identical exposure. Scale bars: 50 µm (cell), 10 µm (mitochondria), 2 µm (zoom). B) Quantification of fluorescence intensity in (A) (n = 3). C) Lipid peroxidation in HK2 and OSRC2 cells assessed using C11‐BODIPY 581/591 (red: reduced; green: oxidized) at 12–36 h post‐transfection with or without 10 µM Fer‐1. Scale bars: 20 µm. D) Immunoblot analysis of ferroptosis defense proteins (GPX4, FSP1, DHODH, SLC7A11, FTH1) in OSRC2 cells at 12, 24, and 36 h after FDX1 overexpression; β‐tubulin as loading control. E) Immunoblots of ferroptosis markers in FDX1‐overexpressing cells treated with Fer‐1 (10 µM), IDE (50 µM), DFO (100 µM), or 7‐DHC (50 µM) for 24 h; β‐tubulin‐normalized expression shown. Data are presented as mean ± SD. ns, not significant; ^*^ *p*< 0.05; ^**^ *p*< 0.01; ^***^ *p*< 0.001; ^****^ *p*< 0.0001, one‐way ANOVA with Dunnett's multiple comparisons test (B).

Given the established role of the mitochondrial respiratory chain—particularly Complex I—as a major source of reactive oxygen species (ROS) and the involvement of FDX1 in mitochondrial electron transfer, we first examined the impact of FDX1 overexpression on oxidative stress. In OSRC2 cells, enforced FDX1 expression led to a time‐dependent increase in total ROS and mitochondrial superoxide levels (MitoSOX), which were detectable from 12–36 h posttransfection, with levels comparable to those induced by 1 µM FAC (**Figure** **2**A,B). In parallel, FDX1 overexpression significantly increased the labile Fe(II) pool between 24 and 36 h, indicating that disrupted iron homeostasis is consistent with ferroptosis initiation. To investigate whether this oxidative‐iron imbalance compromised mitochondrial integrity, MitoTracker staining was performed. Compared with the elongated and reticular mitochondrial networks in control cells, FDX1‐overexpressing cells presented fragmented mitochondria with pronounced perinuclear clustering. Quantitative morphometric analysis revealed significant reductions in the mitochondrial area, perimeter, aspect ratio, and form factor over time (Figure 2A; Figure S2A, Supporting Information). Transmission electron microscopy further confirmed ferroptosis‐associated ultrastructural remodeling, characterized by mitochondrial shrinkage, electron‐dense matrix, crista disruption, outer membrane rupture, and increased autophagosome formation (Figure S2B,C, Supporting Information).

To determine whether lipid peroxidation—a hallmark of ferroptosis—was similarly induced, we employed C11‐BODIPY staining. In OSRC2 cells, FDX1 overexpression caused a marked shift from a reduced (red) to an oxidized (green) C11‐BODIPY signal over time, indicating robust lipid oxidation (Figure 2C; Figure S2D, Supporting Information). In contrast, this oxidative shift was absent in normal renal epithelial HK2 and TEC cells, which retained the reduced lipid form throughout the time course (Figure 2C; Figure S2D, Supporting Information). Notably, ferrostatin‐1 (Fer‐1), a lipid ROS scavenger, failed to fully suppress ROS accumulation and lipid peroxidation in FDX1‐overexpressing OSRC2 cells, suggesting a persistent and possibly overwhelming pro‐oxidant environment (Figure 2A,C). At the molecular level, FDX1 overexpression inhibited multiple essential ferroptosis defense mechanisms, including GPX4, FSP1, DHODH, SLC7A11, and FTH1, with downregulation becoming evident after 24 h (Figure 2E; Figure S2E, Supporting Information). In particular, FTH1, a ferritin subunit essential for iron sequestration, underwent time‐dependent degradation, which was consistent with increased labile iron pools, as corroborated by FerroOrange staining. Notably, this ferroptosis‐associated suppression of defense proteins was selectively observed in ccRCC cells but not in TECs or HK2 controls (Figure S2F, Supporting Information), reinforcing the tumor‐specific nature of the FDX1‐induced ferroptotic response.

To rigorously evaluate the specificity and sufficiency of ferroptosis in facilitating FDX1‐induced cell death, we administered ferroptosis inhibitors—Fer‐1, DFO, idebenone (IDE), and 7‐dehydrocholesterol (7‐DHC)—at the 24 h mark, aligning with the first alterations in ROS, iron buildup, and the inhibition of ferroptosis defenses. Nonetheless, immunoblotting indicated that none of these inhibitors reinstated ferroptosis defense protein levels, and cellular morphology persisted in an abnormal state (Figure 2F). Furthermore, a cell viability analysis demonstrated that these inhibitors did not restore viability in cells overexpressing FDX1 at both 24 and 48 h (Figure S2G, Supporting Information). These results demonstrate that, whereas ferroptosis is a key consequence of FDX1 overexpression, its effects are not fully reversible by traditional ferroptosis inhibitors. These results imply that other pathways—like mitophagy or other underlying mechanisms—that influence irreversible cell fate decisions may be involved in FDX1‐induced mitochondrial dysfunction. Collectively, FDX1 overexpression in ccRCC cells commences a ferroptotic program driven by ROS buildup, lipid peroxidation, iron dysregulation, and mitochondrial structural collapse that was not fully reversed by ferroptosis inhibitors.

### 2.3 FDX1 Promotes Mitophagy and Disrupts Mitochondrial Integrity in ccRCC

**Figure 3:**
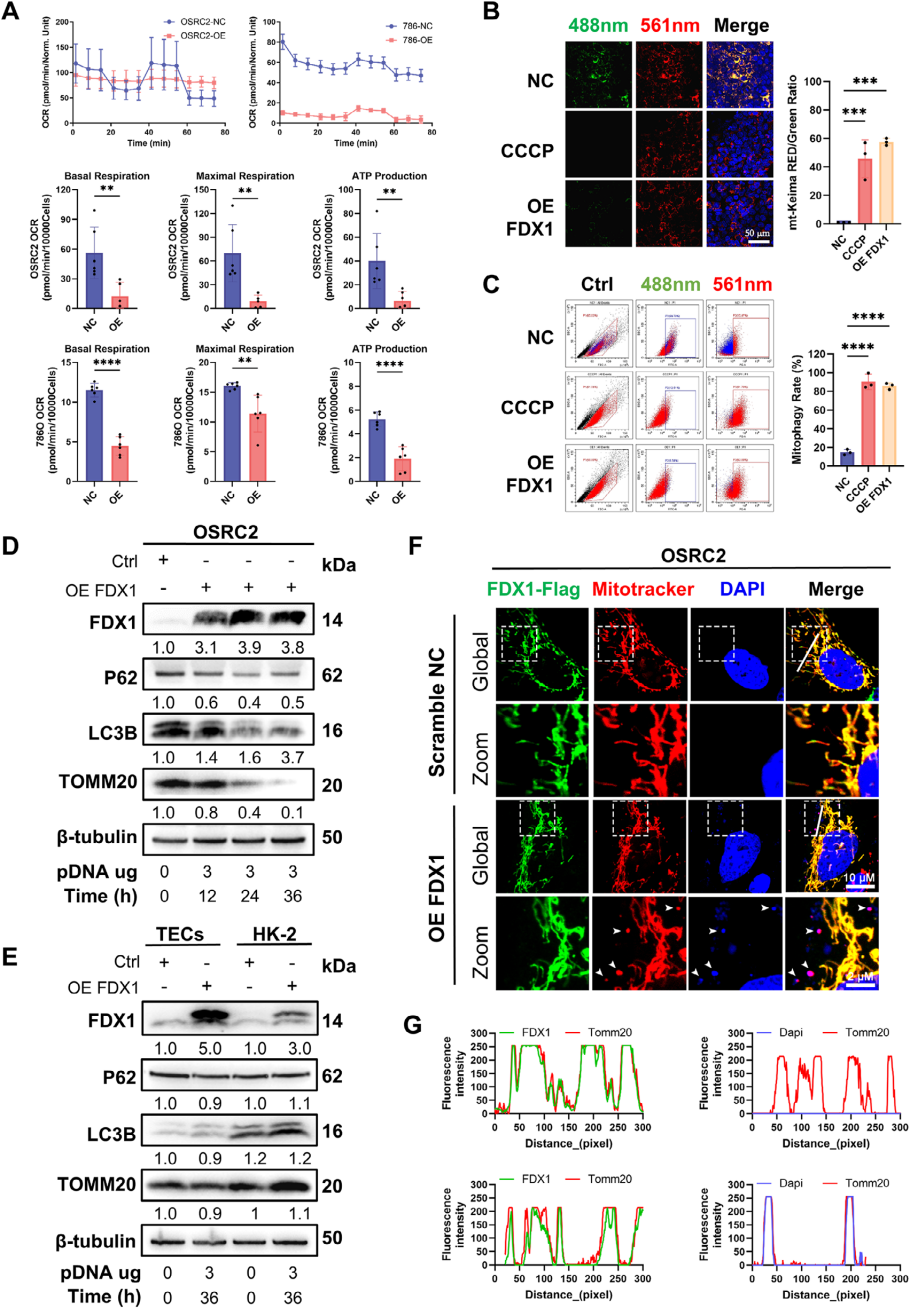
FDX1 overexpression disrupts mitochondrial homeostasis and induces mitophagy. A) Oxygen consumption rate measurements in OSRC2 and 786‐O cells transfected with the control vector or FDX1 overexpression construct, showing basal respiration, maximal respiration, and ATP production (n = 3). B) Mitophagic flux was assessed in OSRC2 cells stably expressing mt‐Keima via confocal microscopy 12 h posttransfection. A fluorescence shift from green (488 nm, mitochondrial localization) to red (561 nm, lysosomal delivery) indicates mitolysosome formation; orange puncta represent mitophagosomes. Positive control: 2 µM CCCP for 3 h. Right panel: Quantification of the red/green fluorescence ratio (n = 30 cells). Scale bar: 50 µm. C) Flow cytometric analysis of mt‐Keima fluorescence (Ex/Em 488/561 nm) under the same conditions as in (B). Quantification of cells with a high red/green fluorescence ratio (n = 3). D) Immunoblot analysis of mitophagy markers (P62, LC3B‐I/II, and TOMM20) in FDX1‐overexpressing OSRC2 cells at the indicated time points. The relative expression normalized to that of β‐tubulin is shown below each blot. E) Immunoblot analysis of mitophagy markers in primary TECs and HK2 cells transfected as indicated. F) Subcellular localization of FDX1‐Flag (green), mitochondria (MitoTracker Red), and nuclei (DAPI) in OSRC2 cells transfected with either the empty vector or the FDX1 overexpression plasmid. The white arrows indicate cytoplasmic mtDNA and nuclear foci. G) Fluorescence intensity profiles along the indicated transect lines. Left: FDX1‐MitoTracker colocalization. Right: DAPI‐MitoTracker colocalization. Scale bar: 10 µm (cell), 2 µm (zoom). Data are presented as mean ± SD. ns, not significant; ^**^ *p*< 0.01; ^***^ *p*< 0.001; ^****^ *p*< 0.0001, Student's *t*‐test (A) and one‐way ANOVA with Dunnett's multiple comparisons test (B and C).

To investigate the downstream effects of FDX1 overexpression on mitochondrial homeostasis, we first assessed mitochondrial function via Seahorse‐based stress assays. Compared with the vector control, FDX1 overexpression significantly reduced oxygen consumption rates (OCRs), including basal respiration, maximal respiration, and ATP‐linked respiration, in both the OSRC2 and 786‐O ccRCC cell lines (**Figure** **3**A). These findings indicate compromised mitochondrial respiration and bioenergetic failure, which is consistent with FDX1‐induced oxidative stress and structural damage (Figure 2A; Figure S2A,B, Supporting Information). Given that defective mitochondria are normally eliminated by mitophagy, we next examined mitophagic flux using OSRC2 cells stably expressing the mt‐Keima reporter, with CCCP as a positive control. Twelve hours posttransfection, confocal imaging indicated a substantial drop in green mt‐Keima fluorescence in FDX1‐overexpressing cells, similar to that in CCCP‐treated cells, indicating improved mitophagic activity (Figure 3B). Flow cytometric quantification confirmed these results, revealing a significant reduction in the green/red fluorescence ratio under both conditions (Figure 3C).

To further substantiate mitophagy induction at the molecular level, we examined mitophagy‐associated proteins by immunoblotting. In OSRC2 cells, FDX1 overexpression led to a time‐dependent increase in the LC3B‐II/I ratio and a reduction in P62 and TOMM20 protein levels, with notable changes occurring as early as 12 h posttransfection (Figure 3D), indicating increased autophagic processing of damaged mitochondria. In contrast, these changes were not detected in normal renal epithelial cells (TECs and HK2), which maintained stable levels of mitophagy markers following FDX1 overexpression (Figure 3E). These findings suggest that FDX1 selectively induces mitophagy in ccRCC cells while sparing normal renal cells.

To elucidate the subcellular localization of FDX1 during this process, we expressed FLAG‐tagged FDX1 in OSRC2 cells. Confocal microscopy revealed strong colocalization of FLAG‐FDX1 with mitochondrial markers (yellow–orange merge), confirming its mitochondrial localization (Figure 3F,G). Intriguingly, punctate DAPI‐positive signals were also observed in the cytoplasm near the mitochondria, which is distinct from nuclear staining. These structures colocalized with mitochondria, implying the release of mitochondrial double‐stranded DNA (mtDNA) from ruptured membranes (Figure 3F,G). While DAPI is a nonspecific dsDNA stain and nuclear contamination cannot be fully excluded, the spatial distribution of these cytoplasmic puncta supports the interpretation of mtDNA release, a known trigger of innate immune responses and a downstream consequence of mitochondrial damage. Together, FDX1 overexpression in FDX1‐deficient ccRCC cells induces a coordinated mitochondrial stress response characterized by impaired respiration, mitophagy activation, and potential mtDNA release, whereas these effects are not observed in normal renal epithelial cells.

### 2.4 FDX1 Activates Type I Interferon Signaling in ccRCC

**Figure 4:**
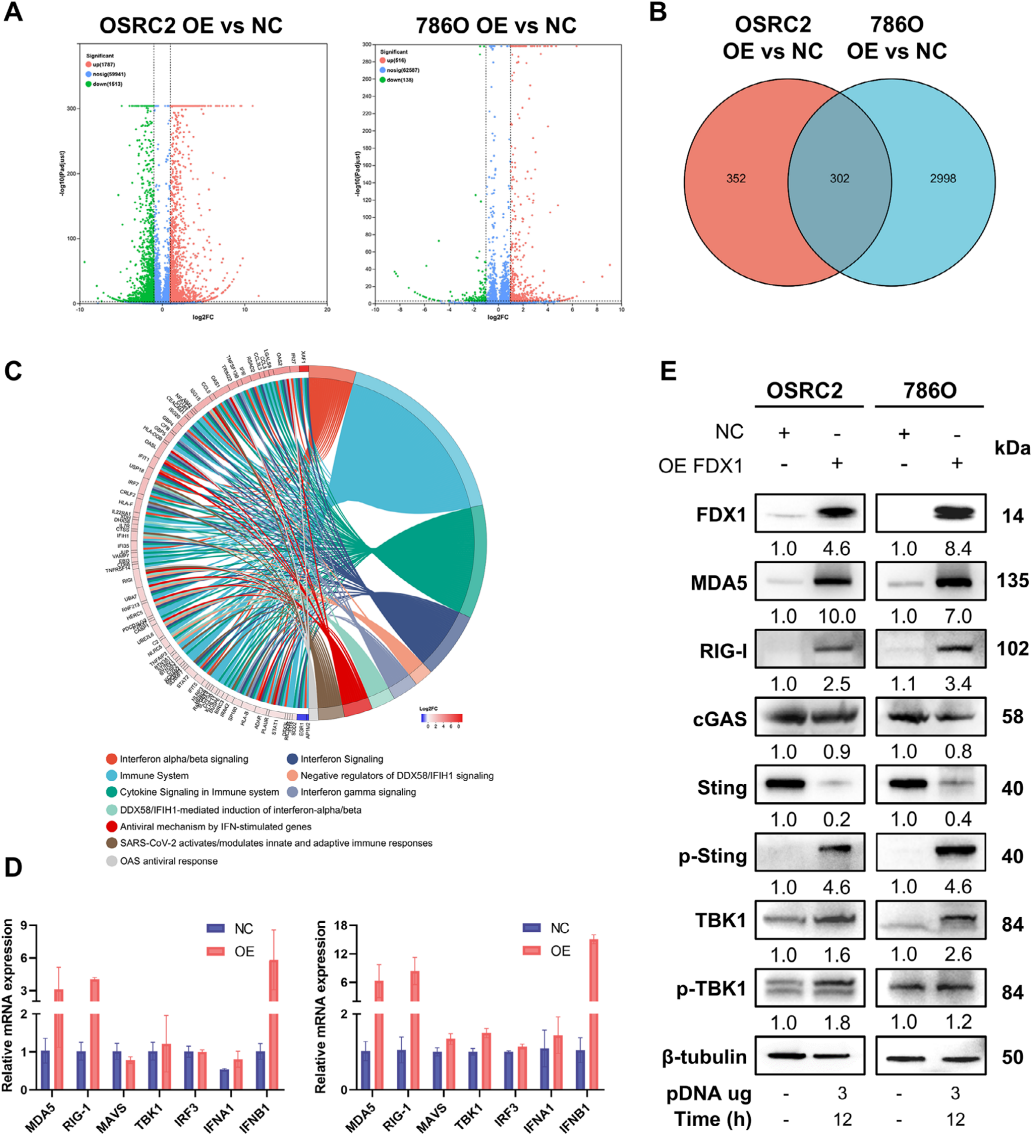
FDX1 overexpression activated the type I interferon pathway in ccRCC cells. A) Volcano plot of differentially expressed genes (DEGs) from RNA‐seq analysis of OSRC2 and 786‐O cells transfected with the FDX1 overexpression construct vs the negative control (NC) (FDR< 0.05). Red: significantly upregulated genes; green: significantly downregulated genes; gray: nonsignificant. Padjust: adjusted *p*‐value. B) Venn diagram showing 302 overlapping DEGs common to both 786‐O and OSRC2 cells. C) chord plot of Reactome pathway enrichment. The right arc displays significantly enriched pathways, whereas the left arc shows DEGs arranged by descending log2‐fold change (log2FC), where a larger log_2_FC indicates stronger upregulation. The annotated pathways included the type I interferon signaling pathway (see figure annotations). D) RT‒qPCR validation of DDX58‐mediated interferon‐α/β pathway genes in OSRC2 and 786‐O cells overexpressing FDX1 (n = 3). E) Immunoblot analysis of key proteins involved in innate immune signaling pathways in OSRC2 and 786‐O cells at 12 h after FDX1 overexpression. The relative protein expression normalized to that of β‐tubulin is indicated below each blot. Data are presented as mean ± SD.

Given the incomplete rescue of cell death by ferroptosis inhibitors and the established interplay between mitophagy and ferroptosis, we hypothesized that FDX1 may also engage innate immune signaling pathways. To investigate this, we performed RNA‐seq analysis of OSRC2 and 786‐O cells following FDX1 overexpression. Transcriptomic profiling revealed extensive differential gene expression, with 1787 genes upregulated and 1513 downregulated in OSRC2 cells and 516 upregulated and 138 downregulated in 786‐O cells (**Figure** **4**A). Reactome and KEGG pathway enrichment analyses of the OSRC2 dataset revealed prominent enrichment in cytokine signaling, TNF/NF‐κB signaling, and type I interferon pathways (Figure S3A,B, Supporting Information), whereas the 786‐O dataset was enriched in interferon alpha/beta signaling, cytosolic DNA‐sensing pathways, and immune responses related to viral defense, including COVID‐19–associated transcriptional signatures (Figure S3C,D, Supporting Information). Venn diagram analysis revealed 302 commonly upregulated genes across both cell lines (Figure 4B), indicating a robust FDX1‐regulated immune response signature. Reactome analysis of this overlapping gene set, which included XAF1, IFI27, and OAS2, confirmed strong enrichment in antiviral innate immunity pathways, particularly Type I interferon signaling and OAS‐mediated antiviral responses (Figure 4C).

To validate these transcriptomic findings, we performed qPCR analysis in both cell lines. FDX1 overexpression significantly upregulated the key RNA sensors MDA5 and RIG‐I, along with a moderate increase in IFNA1 and a pronounced increase in IFNB1 expression (Figure 4D). At the protein level, immunoblotting confirmed increased expression of MDA5 and RIG‐I, accompanied by phosphorylation of the downstream effector kinase TANK binding kinase 1 (TBK1) (Figure 4E), indicating activation of RNA sensor–mediated innate immune signaling. Furthermore, FDX1‐induced mitophagy was associated with the release of mtDNA into the cytoplasm, as previously reported. This cytosolic mtDNA bound and activated the DNA sensor cGAS, leading to the phosphorylation of STING, a hallmark of canonical DNA‐sensing pathway activation (Figure 4E). Notably, total protein levels of cGAS and STING were reduced following FDX1 overexpression, likely due to their turnover via autophagic degradation postactivation (Figure 4E), a mechanism consistent with feedback regulation of innate immune sensors. In contrast, to assess whether FDX1 depletion activates innate immunity, three shRNA constructs targeting FDX1 were generated. Knockdown of FDX1 did not induce activation of MDA5, RIG‐I, or cGAS, nor did it alter ISG15 or IFNB1 expression, indicating that loss of FDX1 fails to elicit innate immune signaling in ccRCC cells (Figure S3E,F, Supporting Information). Collectively, FDX1 activates type I interferon signaling and antiviral immune responses through the dsRNA and the dsDNA sensing pathway in ccRCC.

### 2.5 FDX1 Triggers Mitochondrial Nucleic Acid Release via mPTP Opening and Activates Type I Interferon Signaling at Early Stages

**Figure 5:**
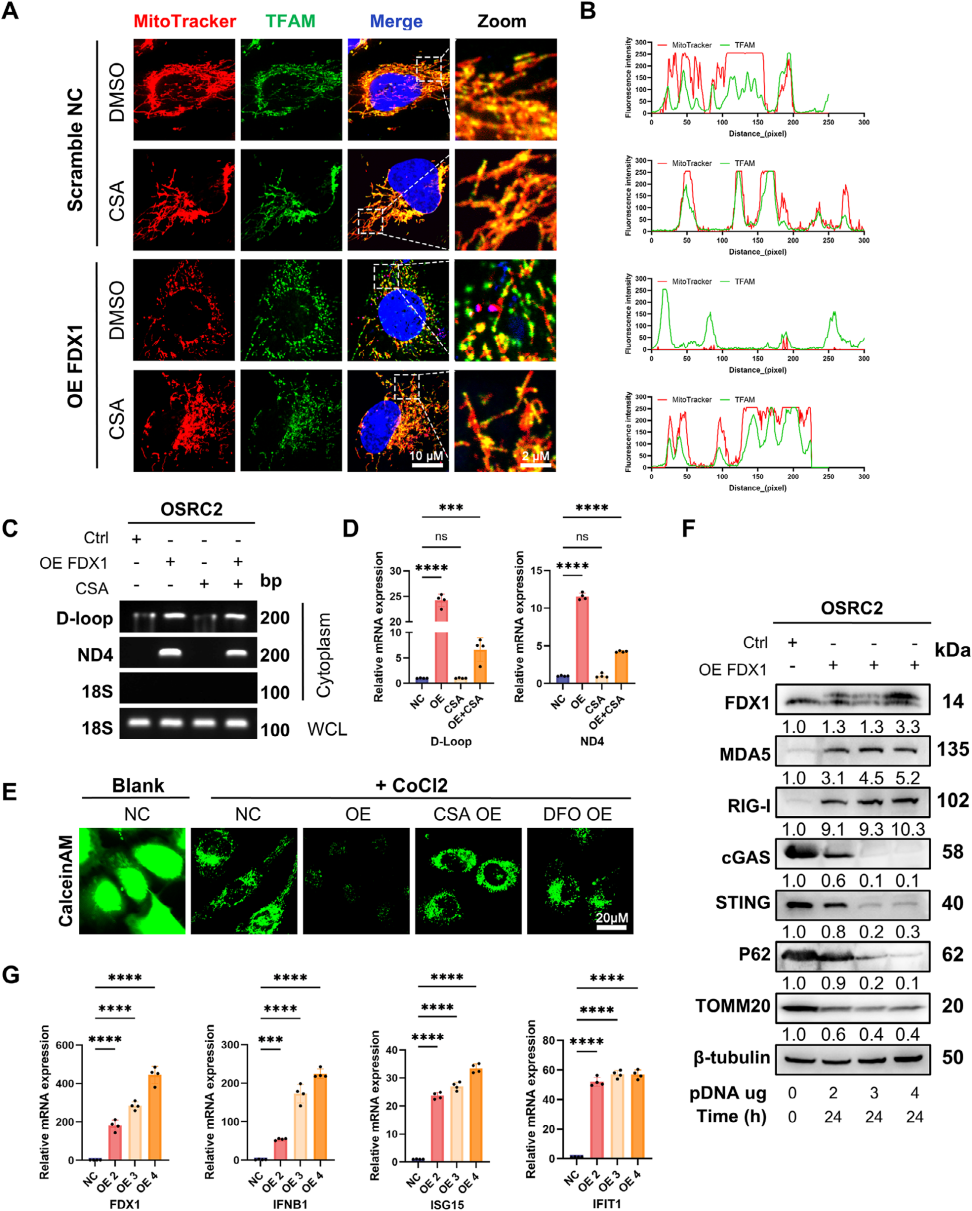
FDX1 overexpression induces mtDNA and mt‐dsRNA release, activating the cGAS–STING and MDA5/RIG‐I signaling pathways. A) Confocal microscopy of OSRC2 cells transfected with the FDX1 overexpression construct for 12 h, with or without 10 µM cyclosporine A (CsA). The cells were stained with MitoTracker Red (mitochondria, 100 nM), anti‐TFAM antibody (mtDNA), and Hoechst 33342 (nuclei). CsA partially inhibits FDX1‐induced mtDNA release. Scale bar: 10 µm (cell), 2 µm (zoom). B) Fluorescence intensity profiles along the transect showing the loss of MitoTracker–TFAM colocalization in FDX1‐overexpressing cells. Nonoverlapping fluorescence peaks (arrows) indicate mtDNA extrusion. C) PCR detection of cytosolic mtDNA via primers targeting the D‐loop and ND4 regions in the subcellular fractions of OSRC2 cells transfected with FDX1 with or without 10 µM CsA. 18S rRNA served as the nuclear DNA control. D) qPCR quantification of cytosolic mtDNA levels normalized to nuclear DNA levels (n = 3). E) Mitochondrial mPTP opening was assessed via calcein‐AM (1 µM) retention after CoCl_2_ (1 mM) quenching in OSRC2 cells treated with FDX1, 10 µM CsA (mPTP inhibitor), or 100 µM DFO (FDX1 synthesis inhibitor). FDX1‐induced mPTP opening is blocked by CsA or DFO. Scale bar: 20 µm. F) Immunoblot analysis of the expression of innate immune sensors (cGAS and STING), mitophagy markers, and viral response proteins in OSRC2 cells transfected with increasing doses of the FDX1 plasmid (0–4 µg mL^−1^ medium, 24 h). Relative protein expression levels normalized to those of β‐tubulin are shown below each blot. G) RT‒qPCR analysis of interferon and interferon‐stimulated genes in OSRC2 cells transfected with increasing doses of the FDX1 plasmid (0–4 µg per 2 mL medium) for 24 h (n = 3), demonstrating dose‐dependent transcriptional activation of innate immune responses. Data are presented as mean ± SD. ns, not significant; ^***^ *p*< 0.001; ^****^ *p*< 0.0001, one‐way ANOVA with Dunnett's multiple comparisons test (D and G).

To directly assess whether FDX1 overexpression drives endogenous mtDNA release, we performed confocal immunofluorescence for mitochondrial transcription factor A (TFAM), which binds mtDNA and helps retain it within mitochondria.^[^ ^27^ ^]^ Compared with vector control cells, FDX1‐overexpressing cells presented markedly reduced TFAM–mtDNA colocalization and a significant increase in the number of cytoplasmic dsDNA puncta (**Figure** **5**A,B). Critically, pretreatment with 5 µM cyclosporine A (CsA), an inhibitor of mitochondrial permeability transition pore (mPTP) opening, restored TFAM–mtDNA colocalization and reduced cytosolic dsDNA accumulation, indicating that the mPTP is the primary conduit for FDX1‐induced mtDNA escape. Consistent with these findings, PicoGreen staining revealed diffuse cytoplasmic dsDNA in FDX1‐overexpressing cells, in contrast with the predominant nuclear fluorescence in the controls (Figure S4A,B, Supporting Information).

Next, we biochemically quantified mtDNA release via NP‐40 subcellular fractionation,^[^ ^28^ ^]^ which selectively permeabilizes the plasma membrane while preserving organelle integrity. PCR amplification of mtDNA‐specific regions (ND4, D‐loop) in the cytoplasmic fraction (normalized to 18S rRNA in whole‐cell lysates) revealed a significant increase in FDX1‐overexpressing cells (Figure 5C). RT‒qPCR confirmed this increase, which was partially reversed by CsA (Figure 5D). Accordingly, CsA pretreatment suppressed FDX1‐induced upregulation of the expression of cytosolic nucleic acid sensors (MDA5 and RIG‐I), type I interferons (IFNA1 and IFNB1), and ISGs (ISG15 and IFIT1) (Figure S4C, Supporting Information). Moreover, 18S rRNA, a nuclear gene marker, was undetectable in the cytoplasmic fractions under either FDX1 overexpression or CsA treatment, thereby excluding the possibility of nuclear contamination (Figure 5C).

Mitochondrial damage under oxidative stress (elevated ROS/mitoSOX/iron) releases DAMPs such as mtDNA and mt‐dsRNA.^[^ ^29^ ^]^ To define the release mechanism, we employed a calcein‐cobalt quenching assay to assess mPTP opening. FDX1‐overexpressing cells exhibited significant calcein quenching—indicative of mPTP opening—while the control cells did not (Figure 5E). As FDX1 increases the amount of labile iron, we found that pretreatment with the iron chelator DFO could partially prevent calcein quenching (Figure 5E,F). Both CsA and the DFO effectively prevented quenching, demonstrating that mPTP opening and iron accumulation are jointly required for mtDNA escape (Figure 5E,F).

Finally, kinetic and dose–response analyses revealed that MDA5 and RIG‑I levels began to rise as early as 6 h posttransfection—in combination with detectable mtDNA/mt‑dsRNA release—while cGAS protein levels declined, reflecting activation‑coupled turnover (Figure S4D, Supporting Information). By 24 h of infection, graded FDX1 expression elicited dose‑dependent increases in MDA5/RIG‑I, decreased P62/TOMM20 (mitophagy markers), and reduced cGAS/STING (activation‑linked degradation), culminating in robust ISG induction (Figure 5F,G). Importantly, qPCR revealed no activation of endogenous retroviruses (Figure S4E, Supporting Information), confirming the mitochondrial rather than the viral origin of the dsRNA signal. Together, FDX1 overexpression rapidly induces mPTP opening and the cytosolic release of mtDNA and mt‑dsRNA that activate the cGAS–STING and MDA5/RIG‑I pathways, eliciting a potent, time‑ and dose‑dependent type I interferon.

### 2.6 FDX1‐Induced dsRNA Accumulation and Immune Activation Precede and are Largely Resistant to Ferroptosis Inhibition

**Figure 6:**
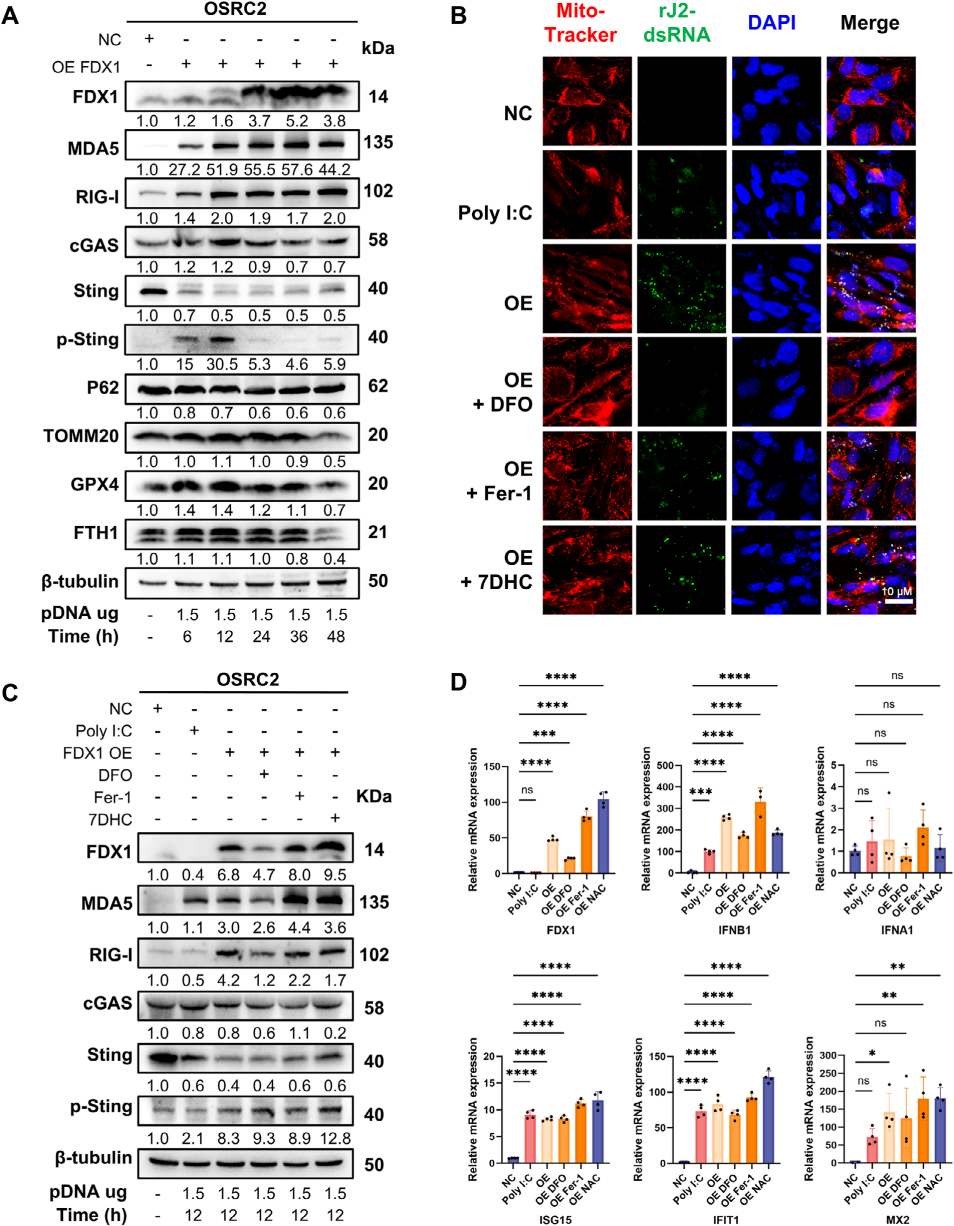
FDX1‐induced mitochondrial double‐stranded RNA accumulation initiates innate immune activation independent of ferroptosis. A) Immunoblot analysis of OSRC2 cells transfected with a low dose of FDX1 plasmid (1.5 µg per 2 mL medium) revealed early upregulation of the expression of the innate immune sensors MDA5 and RIG‐I, which were detectable as early as 6 h posttransfection. The expression of mitophagy markers and ferroptosis defense proteins was also assessed over time. β‐Tubulin served as the loading control. B) Immunofluorescence staining of mitochondrial dsRNA via the J2 antibody (green) and MitoTracker Red (100 nM) in OSRC2 cells overexpressing FDX1 and treated with ferroptosis inhibitors, including DFO 100 µM, 7DHC 50 µM, or Fer‐1 10 µM. Poly(I:C) 20 µg mL^−1^ was used as a positive control, and the empty vector was used as a negative control. DFO, but not Fer‐1 or 7DHC, partially reduced FDX1‐induced dsRNA accumulation. Scale bar: 10 µm. C) Immunoblot analysis of FDX1, RIG‐I, MDA5, cGAS, STING, and phosphorylated STING (p‐STING) in OSRC2 cells transfected with FDX1 or Poly(I:C) for 12 h, followed by treatment with DFO, Fer‐1, or 7DHC. DFO partially attenuated the activation of innate immune proteins by suppressing FDX1 expression, whereas Fer‐1 and 7DHC had minimal effects. β‐Tubulin was used as a loading control. The data are representative of three independent experiments. D) RT‒qPCR analysis of type I interferons and ISGs in OSRC2 cells overexpressing FDX1, with or without DFO, Fer‐1, or 7DHC treatment. Only DFO modestly reduced FDX1‐induced expression of ISGs, suggesting a ferroptosis‐independent mechanism of innate immune activation (n = 4). Data are presented as mean ± SD. ns, not significant; ^*^ *p*< 0.05; ^**^ *p*< 0.01; ^***^ *p*< 0.001; ^****^ *p*< 0.0001, one‐way ANOVA with Dunnett's multiple comparisons test (D).

Our data indicate that FDX1‐induced immune activation (evident at 6 h; Figure S4D, Supporting Information) occurs prior to significant ferroptosis (observed at 24 h; Figure 2A,D). Therefore, we hypothesized that innate immune signaling constitutes an upstream event, whereas ferroptosis represents a downstream cell death pathway. To test this hypothesis, OSRC2 cells transduced with low‐dose FDX1 (1.5 µg/2 mL) were analyzed at early time points (6 h posttransduction). Low‐dose FDX1 activated the RNA sensors MDA5 and RIG‐I within 6 h, with peak STING phosphorylation occurring at 6–12 h (**Figure** **6**A). Notably, the expression of mitophagy (P62 and TOMM20) and ferroptosis defense markers (GPX4 and FTH1) remained unchanged from 6 to 24 h, indicating significant degradation by 48 h (Figure 6A). This finding confirms that FDX1 initially triggers innate immune activation before inducing mitophagy and ferroptosis.

Activated cytosolic RNA sensors MDA5 and RIG‐I signal through the mitochondrial antiviral‐signaling protein (MAVS) adaptor to drive interferon responses. To confirm that this cascade initiates FDX1‐induced inflammation, we generated MAVS‐knockdown OSRC2 cells. Immunoblotting revealed that FDX1‐mediated upregulation of MDA5 and RIG‐I was attenuated in MAVS‐knockdown cells compared with control cells (Figure S5A, Supporting Information). Consequently, FDX1‐induced expression of IFNA1, IFNB1, ISG15, and IFIT1 mRNAs was significantly suppressed by MAVS knockdown (Figure S5B, Supporting Information), demonstrating that MAVS is essential for downstream signaling.

To directly investigate whether ferroptosis inhibition affects the initiation of FDX1‐induced immune signaling, we assessed dsRNA accumulation and sensor activation. Immunofluorescence using the dsRNA‐specific J2 antibody revealed increased cytoplasmic dsRNA puncta in FDX1‐overexpressing cells at 12 h, phenocopying the positive control poly(I:C) (a dsRNA mimic; Figure 6B). This dsRNA colocalized partially with mitochondria, which is also indicative of mitochondrial origin. Importantly, pretreatment with ferroptosis inhibitors and Fer‐1 or 7‐DHC did not reduce the amount of cytoplasmic dsRNA accumulation induced by FDX1 (Figure 6B; Figure S5C, Supporting Information). While the iron chelator DFO reduced dsRNA at an early stage, this effect is likely attributable to its ability to suppress FDX1 expression itself (Figure 1E) rather than ferroptosis inhibition per se.

Consistent with persistent dsRNA, immunoblotting revealed that FDX1‐induced MDA5/RIG‐I upregulation was unaffected by Fer‐1 or 7‐DHC treatment (Figure 6C). DFO modestly reduced MDA5/RIG‐I levels, which was consistent with its suppression of FDX1 expression. qPCR analysis confirmed that FDX1‐induced expression of downstream effectors (IFNA1, IFNB1, ISG15, IFIT1, and MX2) was not suppressed by Fer‐1 or 7‐DHC (Figure 6D). DFO caused modest reductions, again attributable to reduced FDX1. Together, accumulation of dsRNA and immune activation upon FDX1 restoration occur prior to ferroptosis and are largely refractory to ferroptosis inhibitors, although these responses can be partially attenuated by iron chelation.

### 2.7 FDX1 Elevation Inhibits ccRCC Progression while Enhancing Immune Responses In Vivo

**Figure 7:**
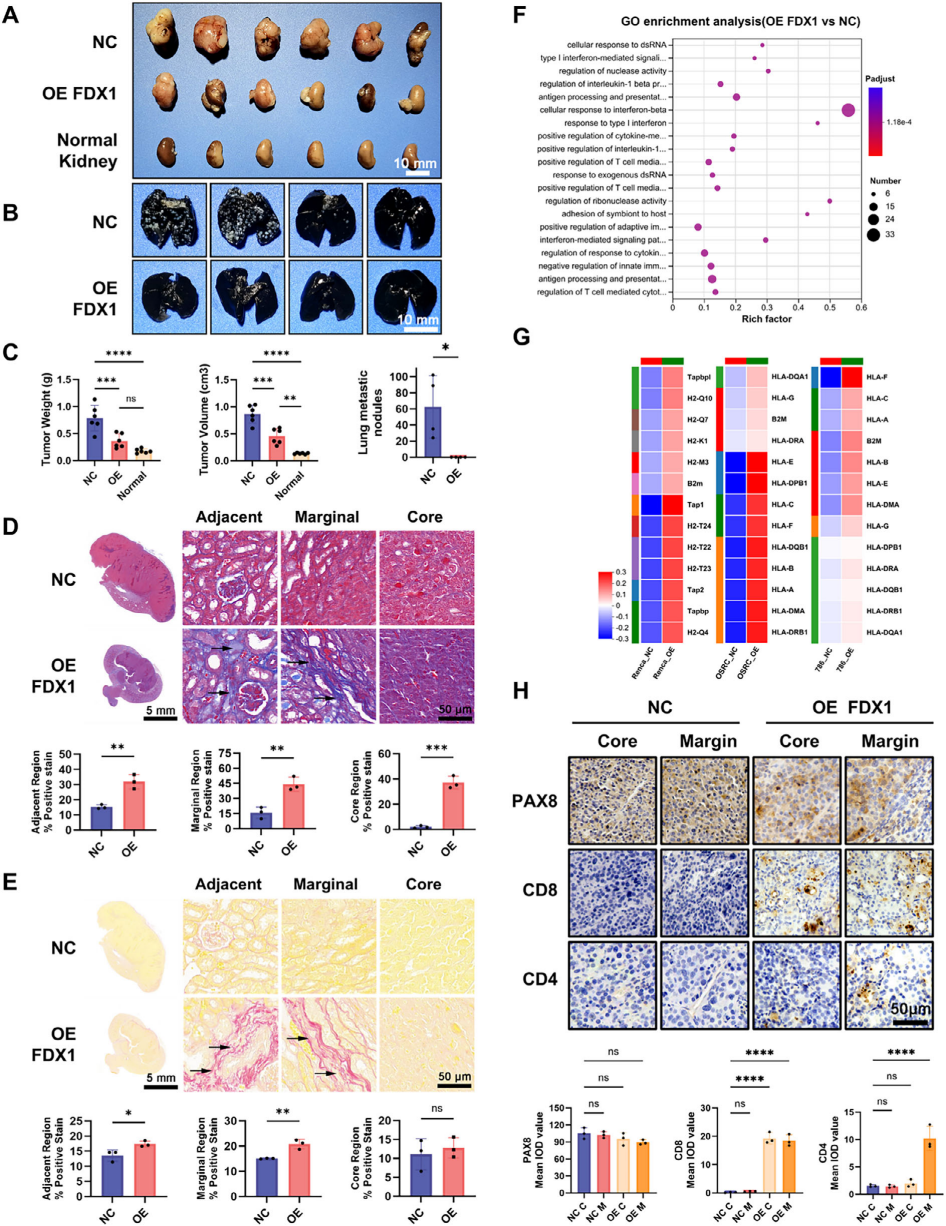
FDX1 overexpression inhibits tumor growth and metastasis while enhancing immune activation in vivo. A) Representative image of tumors harvested from BALB/c mice orthotopically implanted with RENCA cells stably expressing vector control (NC) or FDX1 (OE‐FDX1) (n = 6 per group). Scale bars: 10 mm. B) Representative images of lungs stained with India ink to visualize metastatic nodules (white) two weeks postimplantation (n = 4 per group). Scale bars: 10 mm. C) Quantification of tumor volume, tumor weight, and the number of lung metastatic nodules in the NC and OE‐FDX1 groups. D) Masson's trichrome staining of paraffin‐embedded tumor sections to evaluate fibrosis in the tumor core, margin, and adjacent normal tissue. The fibrotic areas are indicated in blue (black arrows). The bar graphs show the quantification of the fibrotic area. Scale bars: 5 mm (whole section), 50 µm (magnified region). E) Sirius Red staining of collagen fiber deposition in different tumor regions. Red‐stained areas (black arrows) indicate collagen‐positive regions. The quantification of collagen deposition between the groups is shown. Scale bars: 5 mm (whole section), 50 µm (magnified region). F) RNA‐seq analysis of tumor tissues from the NC and OE‐FDX1 groups. GO enrichment of DEGs is shown in a bubble plot; the dot size represents the number of genes, and the color intensity reflects the adjusted *p*‐value. G) Heatmap of MHC antigen presentation‐related DEGs from RNA‐seq of RENCA, OSRC2, and 786‐O cells following FDX1 overexpression. H) Immunohistochemical staining of PAX8 (RCC tumor marker), CD8, and CD4 in RENCA tumor tissues revealed enhanced infiltration of CD8⁺ and CD4⁺ T cells in the OE‐FDX1 group. The quantification of positive staining is shown as the integrated optical density. Scale bars: 50 µm. Data are presented as mean ± SD. ns, not significant; ^*^ *p*< 0.05; ^**^ *p*< 0.01; ^***^ *p*< 0.001; ^****^ *p*< 0.0001, Student's *t*‐test with Mann–Whitney test (C, for lung nodules), Student's *t*‐test (D‐E), and one‐way ANOVA with Dunnett's multiple comparisons test (C and H).

To investigate the in vivo role of FDX1 in ccRCC, we established orthotopic kidney tumors by implanting syngeneic Renca cells into BALB/c mice. Fourteen days postimplantation, the tumors, contralateral kidneys, and lungs were harvested for analysis. Compared with those in the negative control (NC) group transfected with an empty vector, the tumors in the FDX1‐overexpressing (FDX1‐OE) group were significantly smaller and had markedly lower tumor weights and volumes (**Figure** **7**A,C). In the metastasis assays, the NC group developed numerous lung metastases (55.00 ± 53.56 nodules), whereas no metastases were observed in the FDX1‐OE group (Figure 7B,C), indicating that FDX1 suppresses tumor dissemination.

We next assessed inflammation‐associated remodeling of the tumor microenvironment via Masson's trichrome and Sirius red staining. Compared with those of NC controls, Masson's trichrome staining revealed increased collagen deposition (blue), indicative of fibrosis, within the core, margin, and peritumoral regions of FDX1‐OE tumors (Figure 7D). Consistently, Sirius red staining revealed significantly increased collagen deposition (red birefringence) specifically within the tumor margin and adjacent peritumoral tissues of FDX1‐OE mice (Figure 7E), indicating that fibrotic remodeling was associated with FDX1 expression.

RNA sequencing of resected FDX1‐OE vs NC tumors revealed differentially expressed genes (DEGs) (Figure S6A, Supporting Information). Gene Ontology (GO) enrichment analysis of these DEGs revealed significant enrichment in critical immune pathways, including those related to the cellular response to dsRNA, type I interferon signaling, and antigen presentation (Figure 7F). Complementary enrichment analyses via the KEGG and Reactome pathways further revealed involvement of Toll‐like receptor signaling and extracellular matrix organization (Figure S6B,C, Supporting Information), which is consistent with the fibrotic inflammatory alterations observed histologically (Figure 7D,E). Correspondingly, FDX1‐OE tumors presented upregulated expression of major histocompatibility complex (MHC) class I and II genes (Figure 7G), which aligns with enhanced antigen presentation ability. In vitro qPCR validation confirmed the significant upregulation of interferon‐stimulated genes, namely, IFNA4, IFNB1, IFIT1, ISG15, and RSAD2, in FDX1‐OE Renca cells (Figure S6D, Supporting Information). Notably, the expression of the T‐cell‐recruiting chemokines C‐X‐C motif chemokine ligand 9 (CXCL9) and CXCL10 was also substantially elevated (Figure S6D, Supporting Information), indicating that FDX1 promotes immune activation via interferon and chemokine production.

Immunohistochemical analyses supported these findings. Ki67 staining revealed decreased proliferative activity in FDX1‐OE tumors, particularly at the tumor margins (Figure S6E). The expression of the hypoxia marker HIF1α and the angiogenesis marker CD31 was significantly lower in FDX1‐OE tumors than in NC tumors, indicating that FDX1 mitigates hypoxia and suppresses neovascularization (Figure S6E, Supporting Information). Importantly, FDX1‐OE tumors displayed robust infiltration of CD8⁺ T cells throughout the tumor core and periphery, along with CD4⁺ T cells localized to the tumor margins, whereas NC tumors exhibited minimal T‐cell infiltration (Figure 7H). Collectively, these findings demonstrate that FDX1 overexpression potently suppresses ccRCC tumor growth and metastasis in vivo while remodeling the TME to favor immune activation.

## 3 Discussion

**Figure 8:**
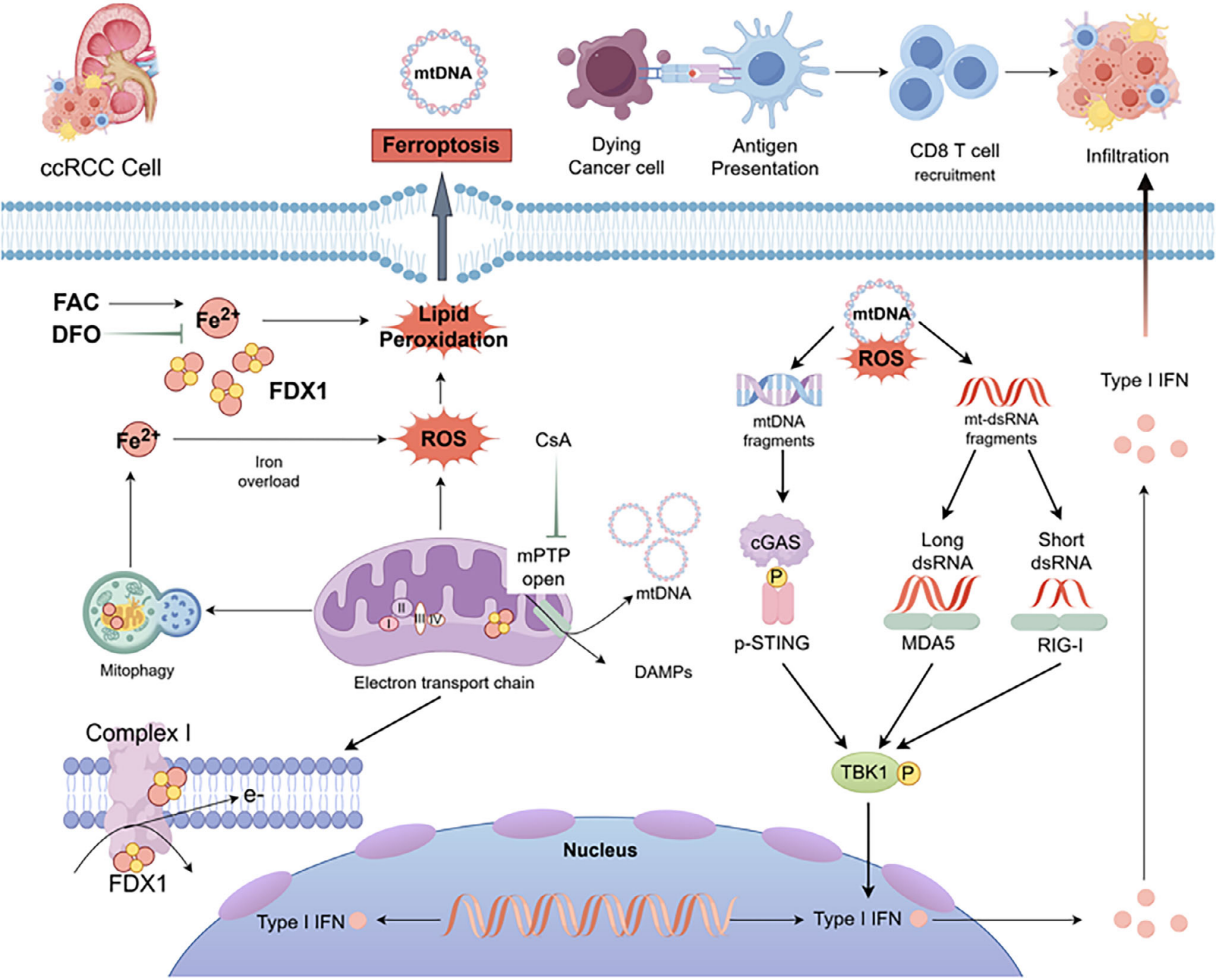
Schematic overview of the dual mechanism by which FDX1 restoration suppresses ccRCC progression. Restoring FDX1 expression triggers two interconnected pathways: 1) It induces ferroptosis characterized by mitochondrial dysfunction, iron accumulation, ROS, and lipid peroxidation. 2) FDX1 promotes mPTP opening, leading to the cytosolic release of mtDNA and mt‐dsRNA. These DAMPs activate innate immune‐sensing pathways, specifically the cGAS‐STING and RIG‐I/MDA5‐MAVS pathways, resulting in the production of type I interferons. This potent antiviral‐like response drives an inflamed tumor microenvironment characterized by collagen remodeling, enhanced MHC I/II antigen presentation, and robust CD8+ T‐cell infiltration.

Our study establishes FDX1 as a pivotal regulator of tumor suppression in ccRCC, demonstrating that its restoration induces ferroptosis while simultaneously triggering innate immune activation through the cytosolic release of mtDNA and mt‐dsRNA (**Figure** **8**). Through comprehensive in vitro and in vivo experiments, we validated this mechanism, revealing that FDX1 restoration disrupts mitochondrial iron metabolism, thereby promoting the accumulation of ROS and elevated mitoSOX levels, which in turn drives lipid peroxidation and culminates in ferroptosis. Concurrently, FDX1 facilitates the release of mtDNA via opening of the mPTP and mt‐dsRNA cytosolic accumulation, activating the cGAS‐STING and RIG‐I/MDA5‐MAVS pathways. This signaling cascade elicits a robust type I interferon response that precedes the onset of ferroptosis, thereby synergistically amplifying antitumor immunity and ultimately inhibiting tumor growth.

This research builds upon and extends prior findings on FDX1, illuminating a dual‐role mechanism that bridges programmed cell death and immune surveillance in ccRCC. Previous reports have consistently documented FDX1 downregulation in ccRCC and its association with poor prognosis.^[^ ^13^ ^]^ Besides, FDX1 was reported to regulate glycolysis,^[^ ^12^ ^]^ fatty acid oxidation,^[^ ^30^ ^]^ amino acid metabolism,^[^ ^30^ ^]^ and indispensable role in cellular survival and embryonic development.^[^ ^31^ ^]^ Since the discovery of FDX1 as a pivotal regulator of cuproptosis,^[^ ^26^ ^]^ pan‐cancer analyses have revealed a strong correlation between its expression and TME,^[^ ^32^, ^33^ ^]^ a finding particularly evident for CD4+ and CD8+ T cell infiltration in ccRCC.^[^ ^13^, ^34^ ^]^ These findings collectively underscore the need for further mechanistic inquiry on inflammatory cell death, and our study uniquely elucidates FDX1's novel functional contributions to orchestrating ferroptosis and innate immunity. Although ferroptosis has been recognized as a potential therapeutic vulnerability in ccRCC,^[^ ^35^ ^]^ the specific involvement of FDX1 in driving iron dysregulation, mitophagy, and subsequent cell death represents a novel mechanistic coordination. Moreover, our discovery that mtDNA/mt‐dsRNA release via mPTP opening serves as a trigger for immune activation further distinguishes this work from prior findings. For instance, recent studies in renal cancer and hereditary leiomyomatosis have identified fumarate hydratase, a key tricarboxylic acid cycle enzyme, whose loss‐of‐function mutations lead to fumarate accumulation; this, in turn, prompts mtDNA release into the cytosol through SNX9‐dependent mitochondrial‐derived vesicles, thereby activating the cGAS‐STING pathway and promoting inflammation and tumorigenesis.^[^ ^36^ ^]^ In contrast, our findings show that endogenous retroviral transcripts remain unchanged upon FDX1 restoration, indicating a mitochondrial—rather than viral—origin for the immunogenic dsRNA, which may be partly attributed to bidirectional transcription of circular mtDNA.^[^ ^37^ ^]^

Our findings demonstrate that immune activation initiated by FDX1 occurs in a cell‐autonomous manner during the early stages, primarily through mtDNA leakage, and is subsequently amplified by the induction of ferroptosis. In contrast to classical inflammatory cell death modalities—such as necroptosis and pyroptosis, where danger signals typically arise from late‐stage membrane rupture—we observed rapid phosphorylation of STING and activation of MDA5/RIG‐I within hours following FDX1 restoration. This early signaling suggests that mtDNA/mt‐dsRNA sensing acts as an upstream amplifier of ferroptosis, rather than a mere secondary effect. Furthermore, induction of ferroptosis in immunologically cold tumors could enhance T‐cell infiltration and synergize with PD‐1 blockade, thereby potentiating antitumor immunity.^[^ ^38^, ^39^ ^]^

In contrast to malignancies reliant on OXPHOS,^[^ ^40^ ^]^ ccRCC is characterized by profound metabolic alterations coupled with suppressed OXPHOS activity.^[^ ^41^ ^]^ Complex I acts as the gatekeeper of the ETC, while FDX1 donates electrons to Complex I, playing an indispensable role in this process. Enhancing ETC flux—specifically through Complex I—emerges as a promising therapeutic avenue in ccRCC. Consistent with this, both primary ccRCC tissues and cell models demonstrate markedly reduced FDX1 expression,^[^ ^13^ ^]^ a phenotype linked to its indispensable role in cellular survival and embryonic development.^[^ ^31^ ^]^ Besides, loss of functions in other cancers has been elucidated mainly in metabolic reprogramming.^[^ ^12^, ^30^, ^42^ ^]^ This inherent low expression complicates functional studies, necessitating overexpression models in our experimental framework. While directly targeting FDX1 pharmacologically remains challenging, our findings suggest viable therapeutic strategies. Clinically accessible ferrous salts increase FDX1 expression at noncytotoxic concentrations. These agents may exploit the elevated labile iron pools in therapy‐resistant tumors^[^ ^43^ ^]^ to potentiate both FDX1‐mediated immunity and ferroptosis. Nutritional adjuvants reported to upregulate FDX1—such as dehydroepiandrosterone, coenzyme Q10, and triiodothyronine—represent additional candidates for exploration.^[^ ^44^ ^]^

Despite these advances, alternative interpretations and unresolved questions remain. First, cGAS‐STING trafficking has been linked to autophagy induction through interactions with self‐ or microbial DNA.^[^ ^45^, ^46^ ^]^ STING‐LC3 interaction^[^ ^46^ ^]^ or ER‒Golgi trafficking^[^ ^45^ ^]^ may contribute to FDX1‐regulated mitophagy, which could play both protective and pro‐death roles.^[^ ^47^ ^]^ Second, while mtDNA/mtRNA are major players, we cannot exclude roles for HMGB1, ATP, or oxidized phospholipids released during ferroptosis.^[^ ^48^ ^]^ For instance, HMGB1 from ferroptotic cells activates macrophages,^[^ ^49^ ^]^ and oxidized lipids may serve as “eat‐me” signals. Third, our data revealed that normal renal epithelial cells are resistant to FDX1 overexpression with no mitophagy or cell death, highlighting context‐specific effects. Differences in baseline antioxidant defenses or iron handling likely underlie this selectivity. Finally, the precise pathways and kinetics of mtDNA/mt‐dsRNA release during FDX1‐induced mitochondrial dysfunction also remain incompletely characterized. Although BAX/BAK oligomerization and voltage‐dependent anion channels have been implicated in mtDNA extrusion,^[^ ^50^, ^51^ ^]^ their relationship with FDX1 warrants further investigation. To address these limitations, future studies should employ live‐cell imaging to track mtDNA dynamics, endogenous gene activation systems via CRISPRa, and single‐cell transcriptomics in patient‐derived xenograft models to better elucidate the underlying regulatory mechanisms and therapeutic potential.

The in vivo data powerfully translate these mechanistic insights into a relevant physiological context. FDX1 overexpression not only suppressed primary tumor growth but also completely abolished lung metastasis. The remodeling of the TME was profound, featuring increased fibrotic collagen deposition, a signature often associated with immune activation and containment. Most importantly, FDX1 expression created an immunogenic TME, characterized by enhanced antigen presentation (upregulated MHC I/II), production of T‐cell chemoattractants (CXCL9/10), and robust infiltration of CD8⁺ and CD4⁺ T cells. This shift from an immunologically “cold” to “hot” TME suggests that FDX1 reactivation could potentially synergize with immunotherapies.

Our study demonstrates that FDX1 restoration in ccRCC triggers a dual tumor‐suppressive mechanism by inducing mitochondrial dysfunction, iron‐dependent ferroptosis, and innate immune activation via mtDNA/mt‐dsRNA release. The interplay between these pathways creates a feed‐forward loop of anti‐tumor immunity and cell death. FDX1‐mediated mPTP opening activates cGAS‐STING and RIG‐I/MDA5 pathways, generating a robust type I interferon response that stimulates immune cell recruitment and enhances tumor immunogenicity. Our findings unveil the therapeutic potential of targeting or mimicking FDX1 function, proposing a novel strategy to simultaneously induce tumor cell killing and ignite innate immune responses for the treatment of ccRCC and potentially other malignancies.

## 4 Experimental Section

### Cell Lines

Human renal epithelial cells (HEK293T, CVCL_0045; HK‐2, CVCL_0302), human renal cancer cell lines (786‐O, CVCL_1051; OSRC‐2, CVCL_1626; SN12C, CVCL_1705), and the murine renal cancer cell line RENCA (CVCL_2174) were procured from Wuhan PunoSai Life Technology Co., Ltd. in December 2023. The murine renal epithelial cell line TCMK‐1 (CVCL_2772) was sourced from Abiowell Biotechnology Co., Ltd. in the same month. All cell lines were authenticated by short tandem repeat (STR) profiling (Supporting Information) and confirmed to be free of mycoplasma contamination. The cells were cultured in DMEM, DMEM‐F12, MEM, or RPMI 1640 medium supplemented with 10% fetal bovine serum (FBS) and 1% penicillin/streptomycin. The RENCA cells were additionally supplemented with 1% sodium pyruvate and nonessential amino acids. All the cell lines were maintained at 37 °C in a humidified incubator with 5% CO_2_.

### Chemicals

The following chemicals were used in this study: ammonium iron(III) citrate (FAC, HY‐B1645), ferrostatin‐1 (HY‐100579), idebenone (HY‐N0303), deferoxamine mesylate (HY‐B0988), 7‐dehydrocholesterol (HY‐113279), elesclomol (HY‐12040), 3‐methyladenine (HY‐19312), MG‐132 (HY‐13259), chloroquine (HY‐17589A), CCCP (HY‐100941), and NH4Cl (HY‐Y1269C), purchased from MedChemExpress; and CuCl2 (751944) from Sigma–Aldrich.

### RNA Isolation and qRT‒PCR

Total RNA was extracted from cells or tissues via the FastPure RNA Isolation Kit (Vazyme, RC112‐01). cDNA synthesis was performed with HiScript III RT SuperMix (Vazyme, R323‐01). qRT‒PCR was performed with iTaq SYBR Green Supermix (Bio‐Rad, 1725122) on a CFX96 system (Bio‐Rad). TUBA1A served as the reference gene; expression levels were calculated via the 2−ΔΔCT method. The primers used are listed in Table S1 (Supporting Information).

### Patient Specimens and Primary Cell Culture

Normal renal epithelial cells and ccRCC tissue samples, including formalin‐fixed paraffin‐embedded (FFPE) samples, were collected from patients undergoing radical nephrectomy at the institution, with informed consent obtained from all participants. Surgical samples were carefully dissected into small pieces and extensively washed with PBS and red blood cell lysis buffer, followed by two rounds of washing. The tissue was then digested via a 1:1 mixture of collagenase I and collagenase II (1–2 mg mL^−1^ in PBS) to create an enzymatic digestion solution. After digestion, the tissue was filtered through progressively smaller filters to obtain a single‐cell suspension. The cells were washed and plated in 6‐well plates, with media replaced every 1–2 days until confluence was reached, at which point they were passaged.

### Plasmid Construction and Infections

Human and murine FDX1 cDNA fragments were amplified via PCR from normal kidney tissues and cloned and inserted into overexpression vectors via restriction enzyme‐based strategies: human FDX1 was ligated into the pLVX‐IRES‐Puro vector (EcoRI/BamHI sites), whereas murine FDX1 was inserted into pLGX‐GSF (EcoRI/AgeI sites), with both constructs generated via homologous recombination. For FDX1 and MAVS knockdown, shRNA sequences (Table S1, Supporting Information) were subcloned and inserted into the pLKO.1 lentiviral vector. Lentiviral particles were produced in HEK293T cells by cotransfecting transfer plasmids with packaging vectors (VSV‐G/pMD2. G and pSPAX2). Plasmid transfection was performed via jetPRIME transfection reagent (Polyplus) according to the manufacturer's protocol. Viral supernatants were harvested at 48 and 72 h posttransfection, concentrated by ultracentrifugation, and titered via qPCR. Target cells were transduced at specified multiplicities of infection (MOIs) with 8 µg mL^−1^ polybrene. Stable polyclonal populations were selected with puromycin (2 µg mL^−1^) for 48 h, and the overexpression/knockdown efficiency was validated by immunoblotting. Owing to the amount of cell death induced by FDX1 overexpression, a transient GOI overexpression system was used to exclude the effect of puromycin screening on cytotoxicity.^[^ ^52^ ^]^ To optimize transient transfection, cells were cultured with the same medium type at 70% cell density, with a plasmid DNA/jetPRIME ratio of 1:2.

### Immunoblotting

Cells or renal tissue samples were lysed in RIPA buffer containing a protease inhibitor cocktail. Protein concentrations were determined via the BCA assay. Equal amounts of protein were mixed with sample loading buffer and heated for denaturation. The protein samples were separated by SDS‒PAGE and transferred to polyvinylidene fluoride (PVDF) membranes via the eBlot L1 fast wet transfer system (GenScript). The membranes were blocked with 5% bovine serum albumin in TBST and incubated overnight with the primary antibody at 4 °C. After washing, the membranes were incubated with a secondary antibody (1:5000) and detected via the StarSignal Chemiluminescent Assay Kit (E171–01, GenStar). Chemiluminescent signals were captured via a chemiluminescent imaging system (Tanon 5200). A list of the antibodies used is provided in Table S2 (Supporting Information).

### Immunohistochemistry

The tissues were washed with PBS, fixed in 4% paraformaldehyde for 48 h, and then dehydrated through a graded alcohol series. After being embedded in paraffin, the tissues were sectioned into slices. The sections were deparaffinized at 65 °C, rehydrated, and subjected to antigen retrieval in citrate buffer. Endogenous peroxidase activity was blocked, followed by incubation with the primary antibody, enhancement reagents, and a biotin‐conjugated secondary antibody. Chromogenic detection was performed via DAB, followed by hematoxylin counterstaining, differentiation, dehydration, and mounting with neutral resin.

### Immunofluorescence and Colocalization Assessment

The cells seeded onto coverslips were incubated overnight, fixed with 4% paraformaldehyde for 20 min, and permeabilized on ice with PBS containing 0.3% Triton X‐100 (PBST) for 5 min to enable antibody penetration. After blocking with 3% normal goat serum, mitochondrial DNA (mtDNA) was visualized by immunostaining mitochondrial transcription factor A (TFAM), which specifically binds mtDNA,^[^ ^27^, ^28^ ^]^ while dsRNA was detected via the J2 monoclonal antibody. Primary antibodies—including anti‐J2 (1:200), anti‐FDX1 (1:200), anti‐Flag (1:800), and anti‐TFAM (1:400)—were applied overnight at 4 °C in a humidified, light‐protected chamber. Species‐appropriate secondary antibodies conjugated to Alexa Fluor or FITC were subsequently incubated for 1 hour. The mitochondria were prelabeled in live cells with 200 nM MitoTracker prior to fixation, and the nuclei were counterstained with DAPI‐containing mounting medium following coverslip mounting. For dsRNA positive controls, cells were transfected with 5 µg mL^−1^ poly(I:C) using JetPrime reagent. Confocal microscopy images were acquired, and mitochondrial colocalization was quantified via Sparse Deconvolution 2.0 for image restoration, followed by Manders’/Pearson's coefficient analysis in ImageJ.

### Cell Viability Assays

The Cell Counting Kit‐8 assay uses WST‐8 to measure cell viability, proliferation, and cytotoxicity. To achieve this goal, 5000 cells were seeded into each well of a 96‐well plate and incubated overnight. After incubation, 10 µL of CCK‐8 solution was added to each well and incubated for an additional 2 h. The absorbance was measured at 450 nm via a microplate reader. The cell viability was calculated. For additional analysis, crystal violet staining was performed. The cells were fixed with 4% paraformaldehyde, stained with 0.5% crystal violet, and destained with acetic acid solution. The destaining solution was transferred to a new 96‐well plate, and the absorbance was measured at 570 nm via a microplate reader.

### LDH Release Assay

Cellular cytotoxicity was quantified via the LDH Cytotoxicity Assay Kit (C0016, Beyotime) according to the manufacturer's specifications. The cells were seeded in 96‐well plates at densities optimized for logarithmic growth (typically 5–10 × 10^3^ cells/well), ensuring ≤80–90% confluency at assay initiation. Following viral transduction or pharmacological treatments with appropriate controls, the plates were centrifuged at 400 × g for 5 min. The supernatants were carefully aspirated, and the supernatant aliquots were transferred to a fresh plate. The reaction mixtures were developed by adding assay reagent and incubating for 30 min at 25 °C under light‐protected conditions, and the absorbance was measured at 490 nm via a microplate reader (Bio‐Tek).

### ROS, mitoSOX, Lipid Peroxidation, Iron Content, and dsDNA Assays

Cellular reactive oxygen species (ROS) were measured via a DCFH‐DA fluorescent probe (10 µM working concentration, Ex/Em 488 nm/525 nm). Mitochondrial superoxide levels were assessed with the MitoSOX Red probe (5 µM working concentration, Ex/Em 510 nm/580 nm). Mitochondrial staining was performed with the MitoTracker Orange CMTMRos probe at a final working concentration of 100–200 nM (Ex/Em 550 nm/580 nm). Lipid peroxidation during ferroptosis was evaluated via C11‐BODIPY581/591 dye, with the cells incubated at 37 °C for 20 min with 3 µM BODIPY 581/591 C11 dye. The oxidized lipid state was quantified by detecting green fluorescence at 510 nm, whereas the reduced lipid state was measured by red fluorescence at 591 nm. To assess iron levels, the FerroOrange probe was employed to detect Fe^2^⁺ in live cells (Ex/Em 561 nm/620 nm). Nuclear staining was performed via Hoechst 33342, and double‐stranded DNA (dsDNA) was visualized via PicoGreen staining. The fluorescence intensity was analyzed via ImageJ, which is based on images captured via confocal microscopy with a 100× oil‐immersion objective. The data were quantified via ImageJ and GraphPad software.

### Transmission Electron Microscopy

The cells were digested with trypsin, centrifuged into cell clumps, and fixed in prechilled 2.5% glutaraldehyde at 4 °C overnight. After fixation, the cell clumps were washed with PBS to remove excess fixative, dehydrated, and then subjected to critical point drying. The samples were then coated with a conductive layer via vacuum sputtering. Finally, images were acquired and analyzed via transmission electron microscopy.

### Mitophagy Flux Assay

Stable cell lines expressing the mt‐Keima plasmid were generated to monitor mitophagy. Mitophagy was quantified via fluorescence microscopy or flow cytometry. The cells were cultured on confocal dishes, treated with CCCP for 3 h, and then transfected with FDX1 overexpression plasmids or empty vectors for 24 h. Imaging was performed via a confocal microscope, and multiple regions were captured for analysis. For flow cytometry, the cells were resuspended in HBSS buffer to create single‐cell suspensions and analyzed for green and red fluorescence. The data were analyzed with ImageJ software, and the mitophagy index was calculated as the percentage of red fluorescence relative to total fluorescence. The mitophagy rate was calculated as follows:

(1) $$\begin{array}{ccc} & & \mathrm{mitophagy}\;\mathrm{flux}\;\mathrm{rate}\%\;=\;\mathrm{red}\;\mathrm{fluorescence}/\\ & & \;\;\;\left(\mathrm{red}\;\mathrm{fluorescence}\;+\;\mathrm{green}\;\mathrm{fluorescence}\right)\times 100\%\; \end{array}$$

### Seahorse OCR Measurements

The oxygen consumption rate (OCR) was measured via a Seahorse XF Cell Mitostress Test Kit and an XF 96 Analyzer (Agilent, USA). Briefly, 3 × 10^4 cells were transfected with either a vehicle control or OE FDX1 plasmid for 24 h and seeded into a 96‐well plate. After overnight incubation, the cells were washed and treated with 1 µM oligomycin, 1 µM FCCP, 0.5 µM rotenone, or 0.5 µM antimycin A. The OCR values were normalized to those of cells, as determined by Hoechst (33342) staining.

### Subcellular Fractionation for Cytosolic DNA Isolation

OSRC2 and 786O cells were seeded in 6‐well plates at a density of 1 × 10⁶ cells per well. Following the experimental treatments, the cells were harvested and resuspended in hypotonic lysis buffer containing 0.25% NP‐40. The cell suspensions were incubated on ice for 15 min to achieve plasma membrane permeabilization while preserving nuclear integrity.^[^ ^53^ ^]^ The resulting lysates were divided equally into two microcentrifuge tubes. One aliquot was designated for total cellular DNA extraction. The second aliquot was centrifuged at 13 500 × g for 15 min at 4 °C to pellet the nuclei and cellular debris. The supernatant, which represented the cytosolic fraction, was carefully transferred to a new tube. Genomic DNA was isolated from both the total cellular fraction and the cytosolic supernatant via the TIANamp Genomic DNA Kit (Cat. P304, TIANGEN Biotech, China), which strictly adhered to the manufacturer's protocol.

### mtDNA Subcellular Isolation and Quantification by PCR and qPCR

Mitochondrial DNA (mtDNA) levels within the cytosolic fraction were quantified via PCR and qPCR. qPCR assays were performed via primer sets specific to the mitochondrial D‐loop region, NADH dehydrogenase subunit 1 (ND1), or NADH dehydrogenase subunit 4 (ND4) genes. Nuclear DNA encoding 18S ribosomal RNA (18S rRNA) served as the reference gene. To pharmacologically inhibit mtDNA release, cells were pretreated with cyclosporin A (CSA; 10 µM) for 6 h prior to fractionation.

DNA samples (10 ng) were amplified via KOD Plus Mix (Cat. # KOD‐201, TOYOBO, Japan) containing each primer (200 nM) specific for the D‐loop, ND4, or 18S rRNA genes. The thermocycling conditions consisted of denaturation, annealing, and extension. The amplified PCR products were separated using 2% agarose gel electrophoresis in 1x TBE buffer at 120 V for 40 min. DNA bands were stained with ethidium bromide (a DNA dye) and visualized under UV light. All primer sequences are listed in Table S1 (Supporting Information).

### Mitochondrial Permeability Transition Pore Opening Assay

mPTP opening activity was monitored with an MPTP Assay Kit (Beyotime, C2009S) according to the manufacturer's protocols. The cells were transiently transfected with FDX1 for 12 h in 35 mm dishes. Then, vehicle‐treated cells were treated with 2 µM CCCP for 3 h as a negative control, while cells treated with 0.5 µM ionomycin for 3 h were used as a positive control. These cells were incubated with 0.01 µM calcein AM for at least 0.5 h at 37 °C. The cytosolic fluorescence was then quenched upon treatment with 0.5 mM CoCl2 for 0.5 h. Mitochondrial calcein fluorescence was maintained or quenched depending on the opening activity of the mPTP. The cells were washed with warm PBS twice and imaged via confocal microscopy.

### Homologous Transplantation of the Orthotopic Tumor Model

Female BALB/c mice (n = 12, 5 weeks old) were obtained from Vital River Laboratory Animal Technology (Beijing, China). The mice were housed in specific‐pathogen‐free facilities maintained at 23 ± 2 °C and 50–60% humidity under a 12 h light/dark cycle, with unrestricted access to food and water. BALB/c mice were randomly assigned to two groups to establish an orthotopic renal tumor model. pLGX empty vector and pLGX‐FDX1‐overexpressing viruses were prepared and transfected into RENCA cells for 24 h. After transfection, the cells were digested, resuspended in PBS, and adjusted to a concentration of 3 × 106 cells mL^−1^. The cell suspension was mixed 1:1 with Matrigel and injected orthotopically into the renal tissue of the mice.

Fourteen days post‐injection, the mice were euthanized. The right atrium was excised, and PBS was injected to flush the organs. Tumor‐bearing and contralateral kidney weights were measured, and the length, width, and height of the kidneys were recorded. The tumor volume was calculated via the following formula

(2) $$volume\left(mm^{3}\right)=\left(W^{2}\times L\right)/2$$

where W is the width and L is the length of the kidney. The kidneys were removed, and the lungs were flushed with Indian ink and then fixed in Fekete's solution for 24 h at room temperature. The number of white tumor nodules on the lung surface was counted to assess lung metastasis.

### Masson's Trichrome and Sirius Red Staining

For Masson's trichrome staining, Deparaffinized and rehydrated sections were sequentially stained with: Weigert's iron hematoxylin (nuclei), Biebrich scarlet‐acid fuchsin solution (cytoplasm/muscle), and phosphomolybdic‐phosphotungstic acid for differentiation. Sections were then counterstained with aniline blue (collagen), rinsed in 1% acetic acid. For Sirius red staining, Deparaffinized and hydrated sections were incubated for 60 min at room temperature in 0.1% Sirius red F3B dissolved in saturated aqueous picric acid. After counterstaining nuclei with Mayer's hematoxylin, sections were washed in acidified water (0.5% acetic acid), dehydrated and mounted with resin. All stained sections underwent whole‐slide digital scanning using TissueFAXS Spectra. Quantitative collagen analysis was performed on these images using ImageJ, applying consistent thresholds within each experimental group.

### RNA Sequencing and Bioinformatic Analysis

The cells were cultured to 70% confluence and transfected with either the pLVX lentivirus empty vector or the pLVX‐FDX1 plasmid. After transfection, the adherent cells were collected, and RNA was extracted with 1 mL of TRIzol reagent. The RNA samples were then sent for sequencing analysis. Gene and transcript quantification was performed to obtain read counts, followed by differential expression analysis between the two groups, with at least two samples per group. Differentially expressed genes were identified using a threshold of Padjust< 0.05 and a fold change ≥2. Multiple hypothesis testing was conducted to calculate the adjusted p value (Padjust). Gene functional annotation and enrichment analysis were performed via the GO, KEGG, and Reactome databases. The results were visualized via volcano plots, differential expression plots, Venn diagrams, and other statistical representations.

### Statistical Analysis

The data were analyzed via standard statistical methods. For comparisons between two groups, an independent two‐tailed Student's *t*‐test was applied. Data are expressed as mean ± standard deviation. One‐way analysis of variance (ANOVA) was used for comparisons involving more than two groups. If ANOVA revealed significant differences, post hoc tests were performed to identify specific group differences. Prior to analysis, the normality of the data was assessed via the Shapiro–Wilk test. For nonnormally distributed data, nonparametric tests, such as the Mann–Whitney U test or Kruskal–Wallis test, were used. All the statistical analyses were conducted with IBM SPSS Statistics 26 or GraphPad Prism 10, with statistical significance defined as a *p* value < 0.05.

### Ethics Approval and Patient Consent Statement

The study was approved by the Hospital Ethics Committee with written informed consent for the use of paraffin‐embedded human tissues. All animal procedures were approved by the Institutional Animal Care and Use Committee (IACUC) of the Chinese People's Liberation Army General Hospital and conducted in accordance with institutional guidelines, including regular oversight, to ensure ethical compliance (Approval No. GPT‐BJAP001).

## Conflict of Interest

The authors declare no conflict of interest.

## Supporting information



Supporting Information

Supporting Information

Supporting Information

Supporting Information

Supporting Information

## Data Availability

The data generated in this study are available within the article and its supplementary data files. All other data supporting the findings of this study are available from the corresponding author upon reasonable request.

## References

[1] E. Jonasch , C. L. Walker , W. K. Rathmell , Nat. Rev. Nephrol. 2021, 17, 245. 33144689 10.1038/s41581-020-00359-2 PMC8172121

[2] Y. Wang , E. R. Suarez , G. Kastrunes , N. S. P. de Campos , R. Abbas , R. S. Pivetta , N. Murugan , G. M. Chalbatani , V. D'Andrea , W. A. Marasco , Mol Cancer 2024, 23, 8. 38195534 10.1186/s12943-023-01911-x PMC10775455

[3] R. J. Motzer , K. Penkov , J. Haanen , B. Rini , L. Albiges , M. T. Campbell , B. Venugopal , C. Kollmannsberger , S. Negrier , M. Uemura , J. L. Lee , A. Vasiliev , W. H. Miller , H. Gurney , M. Schmidinger , J. Larkin , M. B. Atkins , J. Bedke , B. Alekseev , J. Wang , M. Mariani , P. B. Robbins , A. Chudnovsky , C. Fowst , S. Hariharan , B. Huang , A. di Pietro , T. K. Choueiri , N. Engl. J. Med. 2019, 380, 1103. 30779531 10.1056/NEJMoa1816047 PMC6716603

[4] B. I. Rini , E. R. Plimack , V. Stus , R. Gafanov , R. Hawkins , D. Nosov , F. Pouliot , B. Alekseev , D. Soulières , B. Melichar , I. Vynnychenko , A. Kryzhanivska , I. Bondarenko , S. J. Azevedo , D. Borchiellini , C. Szczylik , M. Markus , R. S. McDermott , J. Bedke , S. Tartas , Y.‐H. Chang , S. Tamada , Q. Shou , R. F. Perini , M. Chen , M. B. Atkins , T. Powles , N. Engl. J. Med. 2019, 380, 1116. 30779529 10.1056/NEJMoa1816714

[5] Y. Zou , W. S. Henry , E. L. Ricq , E. T. Graham , V. V. Phadnis , P. Maretich , S. Paradkar , N. Boehnke , A. A. Deik , F. Reinhardt , J. K. Eaton , B. Ferguson , W. Wang , J. Fairman , H. R. Keys , V. Dančík , C. B. Clish , P. A. Clemons , P. T. Hammond , L. A. Boyer , R. A. Weinberg , S. L. Schreiber , Nature 2020, 585, 603. 32939090 10.1038/s41586-020-2732-8 PMC8051864

[6] W.‐J. Chen , X.‐W. Pan , X. Song , Z.‐C. Liu , D. Xu , J.‐X. Chen , K.‐Q. Dong , S.‐C. Di , J.‐Q. Ye , S.‐S. Gan , L.‐H. Wang , W. Zhou , X.‐G. Cui , Cancer Lett. 2024, 593, 216963. 38768682 10.1016/j.canlet.2024.216963

[7] E. Vringer , S. W. G. Tait , Cell Death Differ. 2023, 30, 304. 36447047 10.1038/s41418-022-01094-w PMC9950460

[8] E. Marques , R. Kramer , D. G. Ryan , npj Metabolic Health and Disease 2024, 2, 6. 38812744 10.1038/s44324-024-00008-3 PMC11129950

[9] S. Marchi , E. Guilbaud , S. W. G. Tait , T. Yamazaki , L. Galluzzi , Nat. Rev. Immunol. 2023, 23, 159. 35879417 10.1038/s41577-022-00760-x PMC9310369

[10] H. Xian , K. Watari , E. Sanchez‐Lopez , J. Offenberger , J. Onyuru , H. Sampath , W. Ying , H. M. Hoffman , G. S. Shadel , M. Karin , Immunity 2022, 55, 1370. 35835107 10.1016/j.immuni.2022.06.007 PMC9378606

[11] K. Cui , K. Wang , Z. Huang , J. Experiment. Clinic. Cancer Res. 2024, 43, 1. 10.1186/s13046-024-03235-0 PMC11607824 39614322

[12] V. Schulz , S. Basu , S. A. Freibert , H. Webert , L. Boss , U. Mühlenhoff , F. Pierrel , L. O. Essen , D. M. Warui , S. J. Booker , O. Stehling , R. Lill , Nat. Chem. Biol. 2022, 19, 206. 36280795 10.1038/s41589-022-01159-4 PMC10873809

[13] X. Huang , T. Wang , J. Ye , H. Feng , X. Zhang , X. Ma , B. Wang , Y. Huang , X. Zhang , Front. Genet. 2022, 13, 994741. 36186457 10.3389/fgene.2022.994741 PMC9523472

[14] D. Bezwada , L. Perelli , N. P. Lesner , L. Cai , B. Brooks , Z. Wu , H. S. Vu , V. Sondhi , D. L. Cassidy , S. Kasitinon , S. Kelekar , F. Cai , A. B. Aurora , M. Patrick , A. Leach , R. Ghandour , Y. Zhang , D. Do , P. McDaniel , J. Sudderth , D. Dumesnil , S. House , T. Rosales , A. M. Poole , Y. Lotan , S. Woldu , A. Bagrodia , X. Meng , J. A. Cadeddu , P. Mishra , et al., Nature 2024, 633, 923. 39143213 10.1038/s41586-024-07812-3 PMC11424252

[15] J. A. Mayr , D. Meierhofer , F. Zimmermann , R. Feichtinger , C. Kögler , M. Ratschek , N. Schmeller , W. Sperl , B. Kofler , Clin. Cancer Res. 2008, 14, 2270. 18413815 10.1158/1078-0432.CCR-07-4131

[16] G. Gasparre , E. Hervouet , E. de Laplanche , J. Demont , L. F. Pennisi , M. Colombel , F. Mège‐Lechevallier , J.‐Y. Scoazec , E. Bonora , R. Smeets , J. Smeitink , V. Lazar , J. Lespinasse , S. Giraud , C. Godinot , G. Romeo , H. Simonnet , Hum. Mol. Genet. 2008, 17, 986. 18156159 10.1093/hmg/ddm371

[17] N. Maio , T. A. Rouault , Trends Biochem. Sci. 2020, 45, 411. 32311335 10.1016/j.tibs.2020.02.001 PMC8349188

[18] K. H. Ebrahimi , S. Ciofi‐Baffoni , P. L. Hagedoorn , Y. Nicolet , N. E. Le Brun , W. R. Hagen , F. A. Armstrong , Nat. Chem. 2022, 14, 253. 35165425 10.1038/s41557-021-00882-0

[19] B. T. Paul , D. H. Manz , F. M. Torti , S. V. Torti , Expert Rev. Hematol. 2017, 10, 65. 27911100 10.1080/17474086.2016.1268047 PMC5538026

[20] B. S. Padman , M. Bach , G. Lucarelli , M. Prescott , G. Ramm , Autophagy 2013, 9, 1862. 24150213 10.4161/auto.26557

[21] R. A. Weber , F. S. Yen , S. P. V. Nicholson , H. Alwaseem , E. C. Bayraktar , M. Alam , R. C. Timson , K. La , M. Abu‐Remaileh , H. Molina , K. Birsoy , Mol. Cell 2020, 77, 645. 31983508 10.1016/j.molcel.2020.01.003 PMC7176020

[22] K. F. Yambire , C. Rostosky , T. Watanabe , D. Pacheu‐Grau , S. Torres‐Odio , A. Sanchez‐Guerrero , O. Senderovich , E. G. Meyron‐Holtz , I. Milosevic , J. Frahm , A. P. West , N. Raimundo , Elife 2019, 8, 51031. 10.7554/eLife.51031 PMC6917501 31793879

[23] Y. Hou , S. Wang , L. Jiang , X. Sun , J. Li , N. Wang , X. Liu , X. Yao , C. Zhang , H. Deng , G. Yang , J. Agric. Food Chem. 2022, 20, 6213. 10.1021/acs.jafc.1c08349 35543324

[24] E. Q. Roldan , G. Biasiotto , P. Magro , I. Zanella , Pharmacol. Res. 2020, 158, 104904. 32430286 10.1016/j.phrs.2020.104904 PMC7217799

[25] F. Fiorito , C. Irace , F. P. Nocera , M. Piccolo , M. G. Ferraro , R. Ciampaglia , G. C. Tenore , R. Santamaria , L. De Martino , Res. Vet. Sci. 2021, 137, 1. 33906007 10.1016/j.rvsc.2021.04.023

[26] P. Tsvetkov , S. Coy , B. Petrova , M. Dreishpoon , A. Verma , M. Abdusamad , J. Rossen , L. Joesch‐Cohen , R. Humeidi , R. D. Spangler , J. K. Eaton , E. Frenkel , M. Kocak , S. M. Corsello , S. Lutsenko , N. Kanarek , S. Santagata , T. R. Golub , Science 2022, 375, 1254. 35298263 10.1126/science.abf0529 PMC9273333

[27] H. Liu , C. Zhen , J. Xie , Z. Luo , L. Zeng , G. Zhao , S. Lu , H. Zhuang , H. Fan , X. Li , Z. Liu , S. Lin , H. Jiang , Y. Chen , J. Cheng , Z. Cao , K. Dai , J. Shi , Z. Wang , Y. Hu , T. Meng , C. Zhou , Z. Han , H. Huang , Q. Zhou , P. He , D. Feng , Nat. Cell Biol. 2024, 26, 878. 38783142 10.1038/s41556-024-01419-6

[28] H. Liu , H. Fan , P. He , H. Zhuang , X. Liu , M. Chen , W. Zhong , Y. Zhang , C. Zhen , Y. Li , H. Jiang , T. Meng , Y. Xu , G. Zhao , D. Feng , EMBO J. 2022, 41, 111173. 10.15252/embj.2022111173 PMC9753472 36245295

[29] X. Xu , Y. Pang , X. Fan , Signal Transduct. Target Ther. 2025, 10, 190. 40500258 10.1038/s41392-025-02253-4 PMC12159213

[30] Z. Zhang , Y. Ma , X. Guo , Y. Du , Q. Zhu , X. Wang , C. Duan , Front. Pharmacol. 2021, 12, 749134. 34690780 10.3389/fphar.2021.749134 PMC8531531

[31] S. Mohibi , Y. Zhang , V. Perng , M. Chen , J. Zhang , X. Chen , Elife 2024, 13, 91656. 10.7554/eLife.91656 PMC10846857 38251655

[32] R. Zhao , O. Sukocheva , E. Tse , M. Neganova , Y. Aleksandrova , Y. Zheng , H. Gu , D. Zhao , S. V. Madhunapantula , X. Zhu , J. Liu , R. Fan , Cell Commun. Signal 2024, 22, 379. 39068453 10.1186/s12964-024-01743-2 PMC11282696

[33] L. Yang , Y. Zhang , Y. Wang , P. Jiang , F. Liu , N. Feng , Front. Pharmacol. 2022, 13, 938134. 36210836 10.3389/fphar.2022.938134 PMC9532935

[34] M. Xie , B. Cheng , S. Yu , Y. He , Y. Cao , T. Zhou , K. Han , R. Dai , R. Wang , Cells 2022, 12, 173. 36611966 10.3390/cells12010173 PMC9818076

[35] Y. Zou , M. J. Palte , A. A. Deik , H. Li , J. K. Eaton , W. Wang , Y.‐Y. Tseng , R. Deasy , M. Kost‐Alimova , V. Dančík , E. S. Leshchiner , V. S. Viswanathan , S. Signoretti , T. K. Choueiri , J. S. Boehm , B. K. Wagner , J. G. Doench , C. B. Clish , P. A. Clemons , S. L. Schreiber , Nat. Commun. 2019, 10, 1617. 30962421 10.1038/s41467-019-09277-9 PMC6453886

[36] V. Zecchini , V. Paupe , I. Herranz‐Montoya , J. Janssen , I. M. N. Wortel , J. L. Morris , A. Ferguson , S. R. Chowdury , M. Segarra‐Mondejar , A. S. H. Costa , G. C. Pereira , L. Tronci , T. Young , E. Nikitopoulou , M. Yang , D. Bihary , F. Caicci , S. Nagashima , A. Speed , K. Bokea , Z. Baig , S. Samarajiwa , M. Tran , T. Mitchell , M. Johnson , J. Prudent , C. Frezza , Nature 2023, 615, 499. 36890229 10.1038/s41586-023-05770-w PMC10017517

[37] Y. G. Chen , S. Hur , Nat. Rev. Mol. Cell Biol. 2022, 23, 286. 34815573 10.1038/s41580-021-00430-1 PMC8969093

[38] L. Yu , K. Huang , Y. Liao , L. Wang , G. Sethi , Z. Ma , Cell Prolif 2024, 57, 13644. 10.1111/cpr.13644 PMC11294428 38594879

[39] P. Liao , W. Wang , W. Wang , I. Kryczek , X. Li , Y. Bian , A. Sell , S. Wei , S. Grove , J. K. Johnson , P. D. Kennedy , M. Gijón , Y. M. Shah , W. Zou , Cancer Cell 2022, 40, 365. 35216678 10.1016/j.ccell.2022.02.003 PMC9007863

[40] J. R. Molina , Y. Sun , M. Protopopova , S. Gera , M. Bandi , C. Bristow , T. McAfoos , P. Morlacchi , J. Ackroyd , A.‐N. A. Agip , G. Al‐Atrash , J. Asara , J. Bardenhagen , C. C. Carrillo , C. Carroll , E. Chang , S. Ciurea , J. B. Cross , B. Czako , A. Deem , N. Daver , J. F. de Groot , J.‐W. Dong , N. Feng , G. Gao , J. Gay , M. G. Do , J. Greer , V. Giuliani , J. Han , et al., Nat. Med. 2018, 24, 1036. 29892070 10.1038/s41591-018-0052-4

[41] Y. Zhang , S. Zhang , H. Sun , L. Xu , Cell Death Discovery 2025, 11, 186. 40253354 10.1038/s41420-025-02479-9 PMC12009291

[42] M. B. Dreishpoon , N. R. Bick , B. Petrova , D. M. Warui , A. Cameron , S. J. Booker , N. Kanarek , T. R. Golub , P. Tsvetkov , J. Biol. Chem. 2023, 299, 105046. 37453661 10.1016/j.jbc.2023.105046 PMC10462841

[43] L. V. Loftus , L. T. A. Rolle , B. Wang , K. J. Pienta , S. R. Amend , Int. J. Mol. Sci. 2025, 26, 4193. 40362430 10.3390/ijms26094193 PMC12072162

[44] C. C. Wu , C. J. Li , L. T. Lin , Z. H. Wen , J. T. Cheng , K. H. Tsui , Nutrients 2024, 16, 10. 10.3390/nu16101470 PMC11123998 38794708

[45] X. Gui , H. Yang , T. Li , X. Tan , P. Shi , M. Li , F. Du , Z. J. Chen , Nature 2019, 567, 262. 30842662 10.1038/s41586-019-1006-9 PMC9417302

[46] D. Liu , H. Wu , C. Wang , Y. Li , H. Tian , S. Siraj , S. A. Sehgal , X. Wang , J. Wang , Y. Shang , Z. Jiang , L. Liu , Q. Chen , Cell Death Differ. 2019, 26, 1735. 30568238 10.1038/s41418-018-0251-z PMC6748081

[47] M. Yang , X. Wei , X. Yi , D.‐S. Jiang , Cell Death Dis. 2024, 15, 505. 39013891 10.1038/s41419-024-06804-5 PMC11252137

[48] I. Efimova , E. Catanzaro , L. Van der Meeren , V. D. Turubanova , H. Hammad , T. A. Mishchenko , M. V. Vedunova , C. Fimognari , C. Bachert , F. Coppieters , S. Lefever , A. G. Skirtach , O. Krysko , D. V. Krysko , J. Immunother. Cancer 2020, 8, 001369. 10.1136/jitc-2020-001369 PMC7668384 33188036

[49] Z. Feng , F. Meng , F. Huo , Y. Zhu , Y. Qin , Y. Gui , H. Zhang , P. Lin , Q. He , Y. Li , J. Geng , J. Wu , Redox Biol. 2024, 75, 103255. 39029270 10.1016/j.redox.2024.103255 PMC11304870

[50] K. McArthur , L. W. Whitehead , J. M. Heddleston , L. Li , B. S. Padman , V. Oorschot , N. D. Geoghegan , S. Chappaz , S. Davidson , H. San Chin , R. M. Lane , M. Dramicanin , T. L. Saunders , C. Sugiana , R. Lessene , L. D. Osellame , T.‐L. Chew , G. Dewson , M. Lazarou , G. Ramm , G. Lessene , M. T. Ryan , K. L. Rogers , M. F. van Delft , B. T. Kile , Science 2018, 359,6378. 10.1126/science.aao6047 29472455

[51] J. Kim , R. Gupta , L. P. Blanco , S. Yang , A. Shteinfer‐Kuzmine , K. Wang , J. Zhu , H. E. Yoon , X. Wang , M. Kerkhofs , H. Kang , A. L. Brown , S.‐J. Park , X. Xu , E. Zandee van Rilland , M. K. Kim , J. I. Cohen , M. J. Kaplan , V. Shoshan‐Barmatz , J. H. Chung , Science 2019, 366, 1531. 31857488 10.1126/science.aav4011 PMC8325171

[52] Y. Fu , Z. Han , W. Cheng , S. Niu , T. Wang , X. Wang , Appl. Microbiol. Biotechnol. 2024, 108, 480. 39365308 10.1007/s00253-024-13315-y PMC11452495

[53] H. Liu , H. Zhuang , L. Zeng , J. Xie , K. Qiu , D. Feng , Mitochondrial Commun. 2024, 2, 100.

