# Supplementary figures and images for: Fe‐S Protein FDX1 Triggers Tumor‐Intrinsic Innate Immunity via Mitochondrial Nucleic Acids Release to Orchestrate Ferroptosis in CCRCC
Source: Adv Sci (Weinh). 2025 Nov 7;13(6):e18323. doi: 10.1002/advs.202518323 (PMC12866870; doi:10.1002/advs.202518323)

**Supplemental Material**

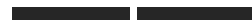

**Original Western Blots  
& PCR**

1D

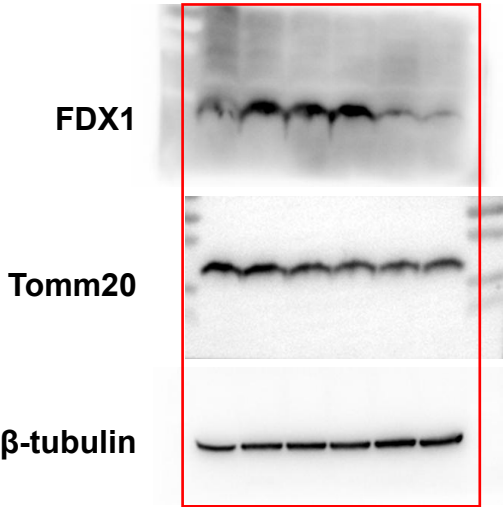

1E

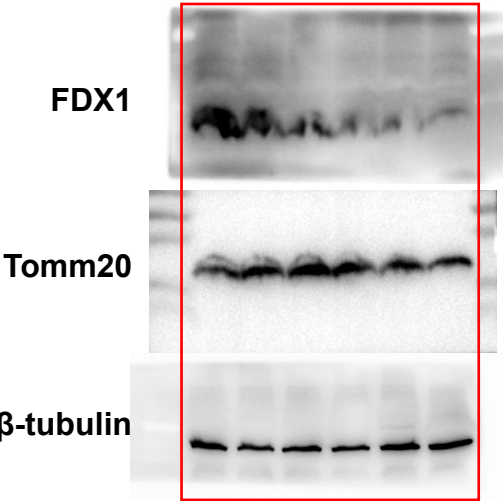

1F

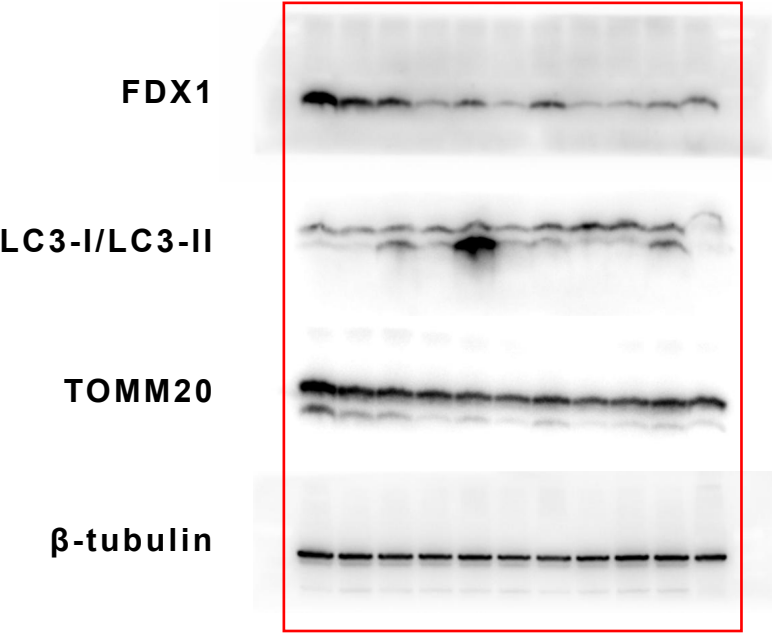

2D

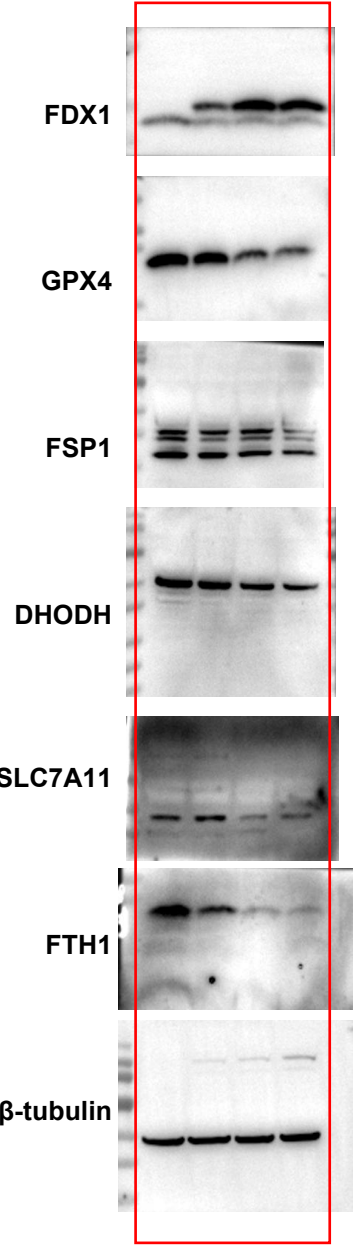

2E

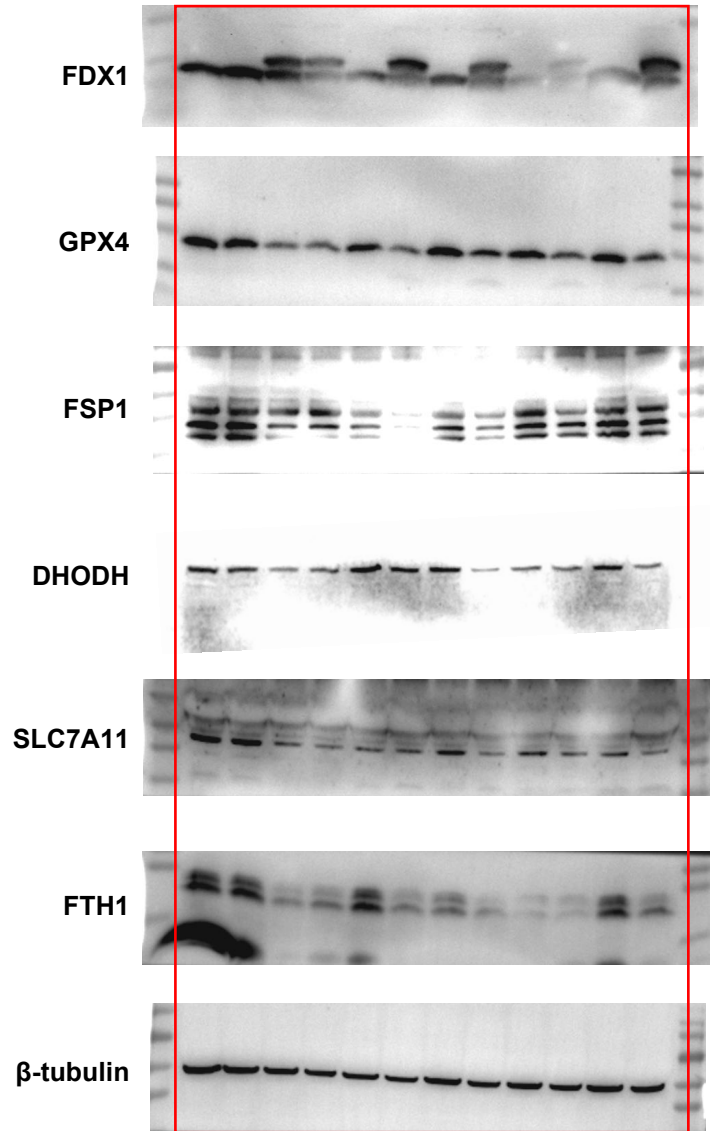

3D

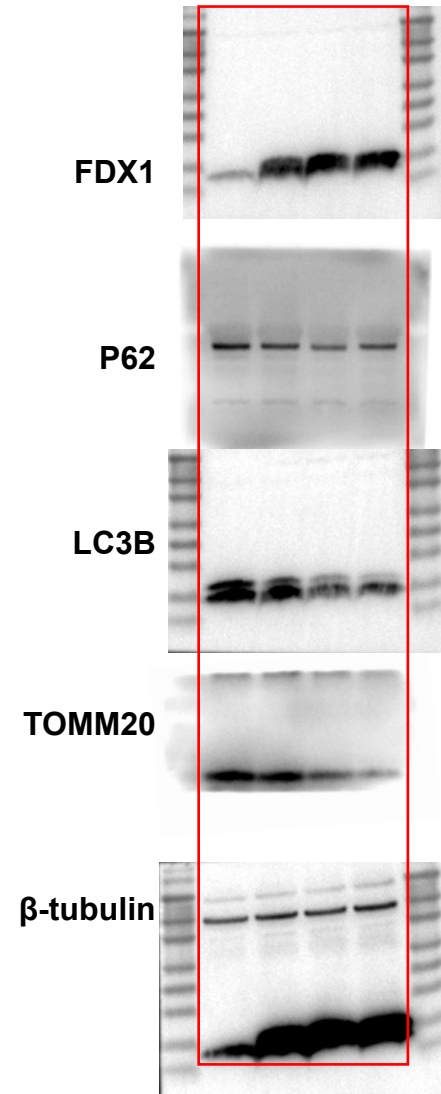

3E

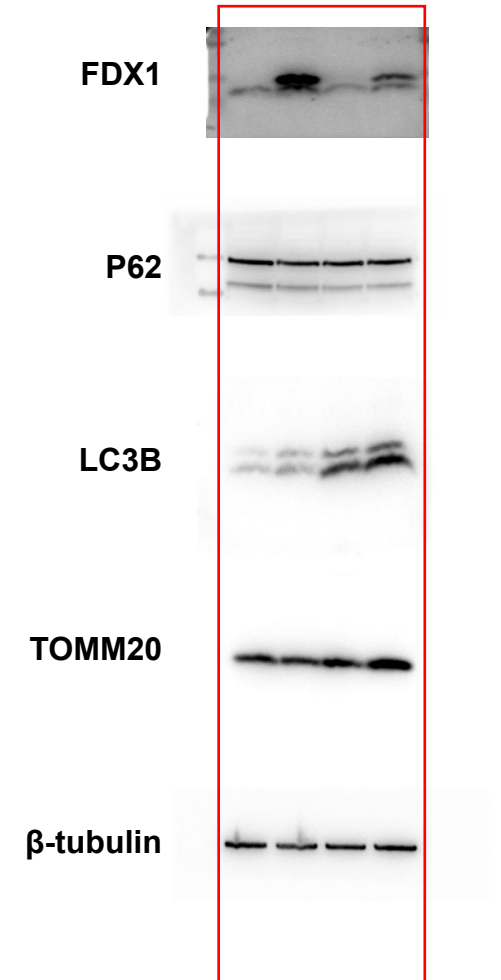

4E

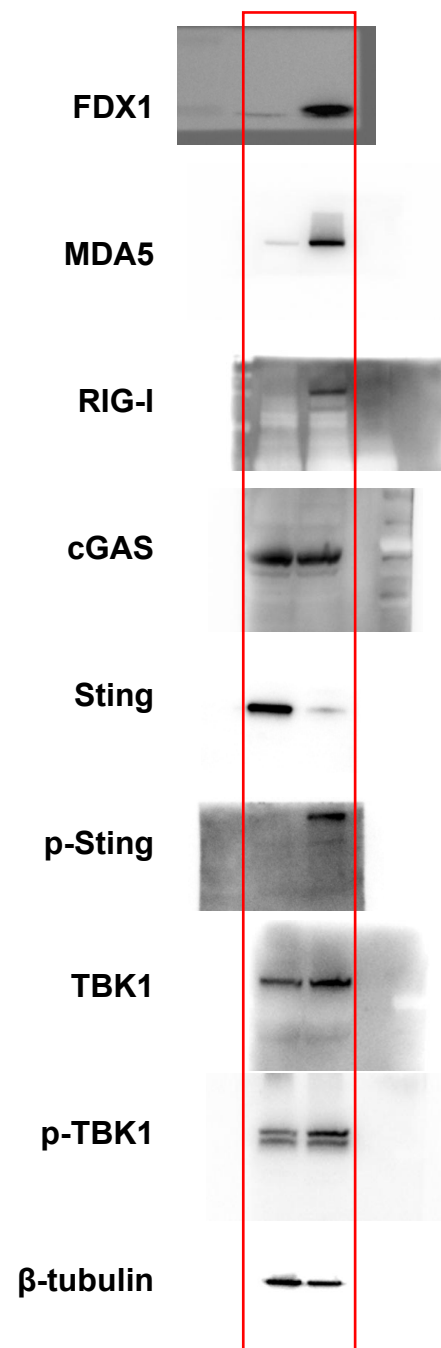

5C

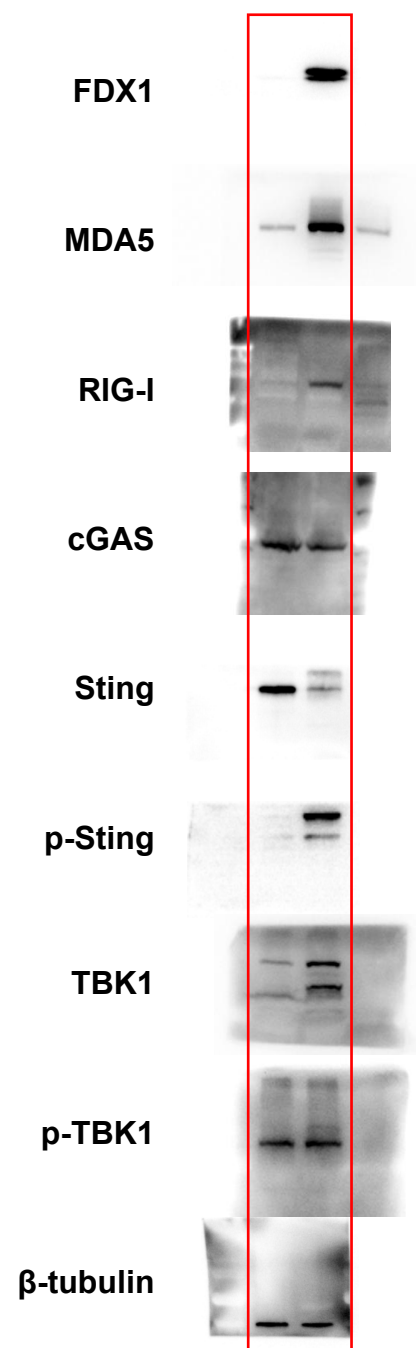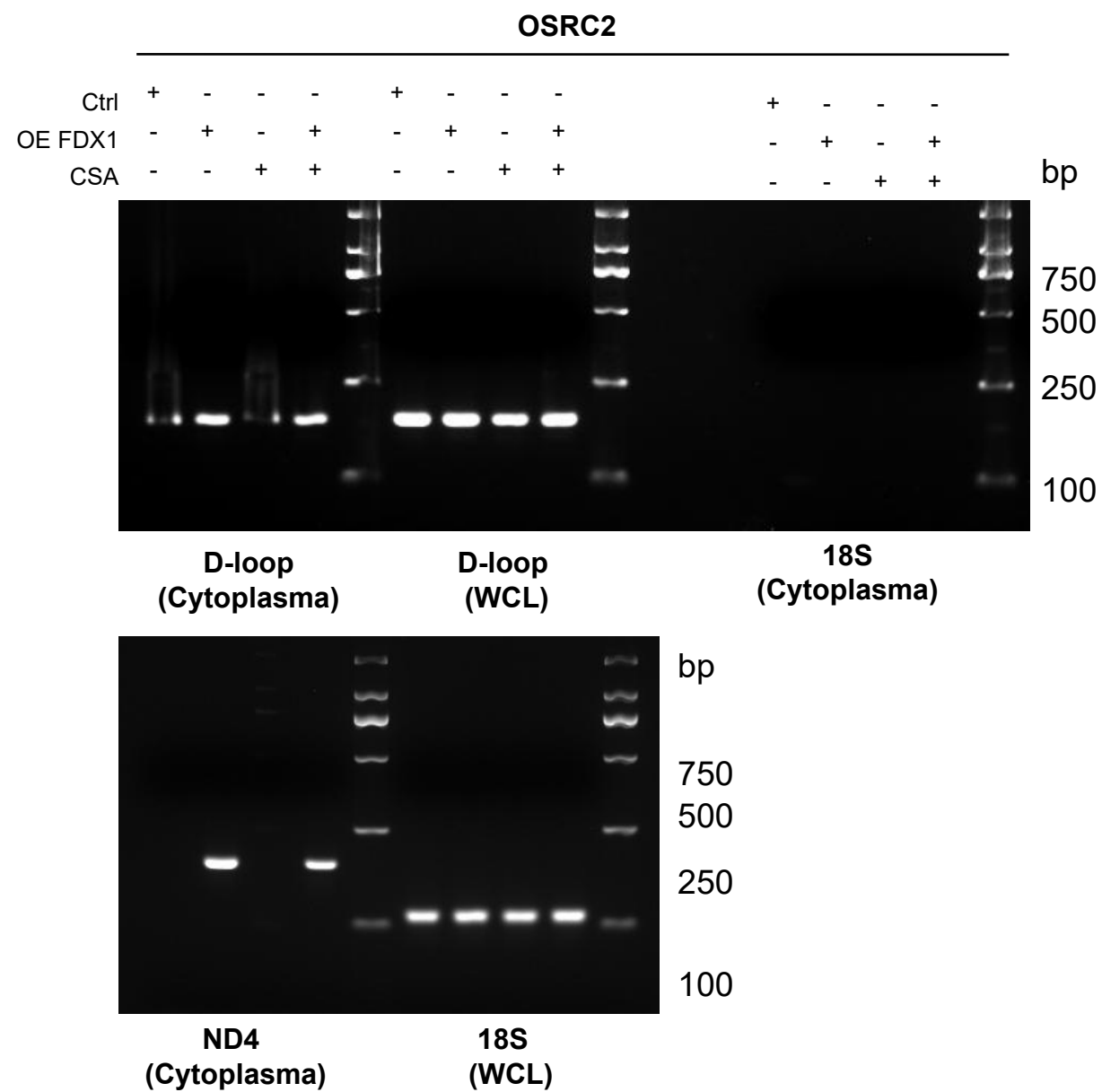

**5F**

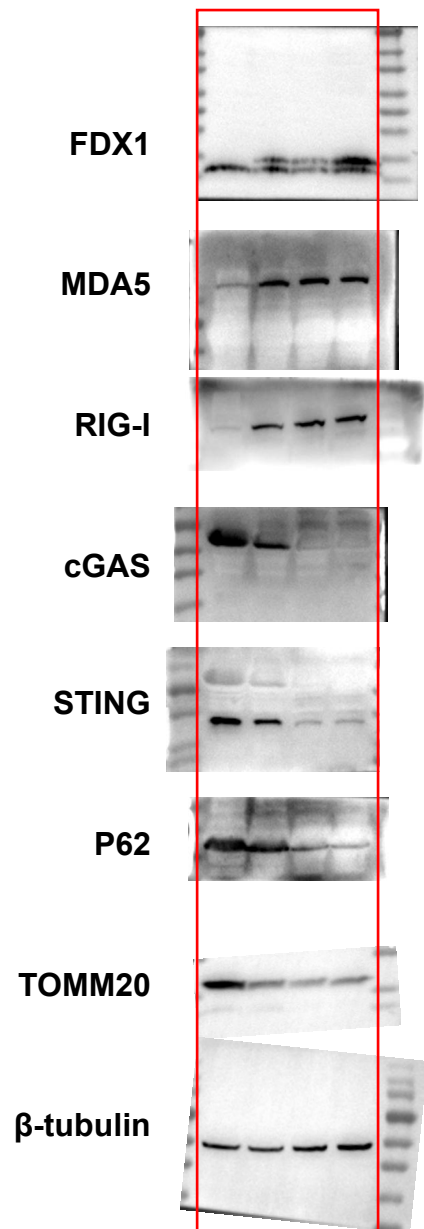

**6A**

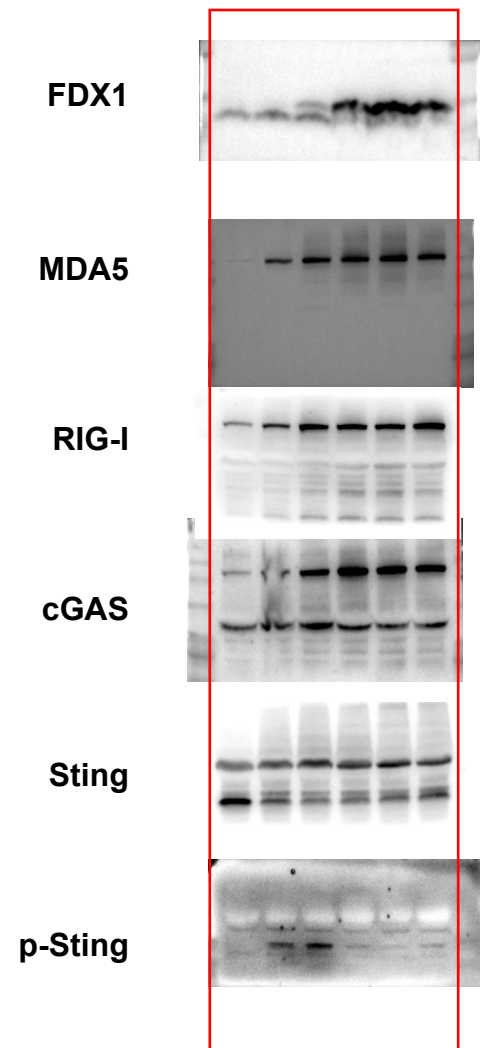

**6C**

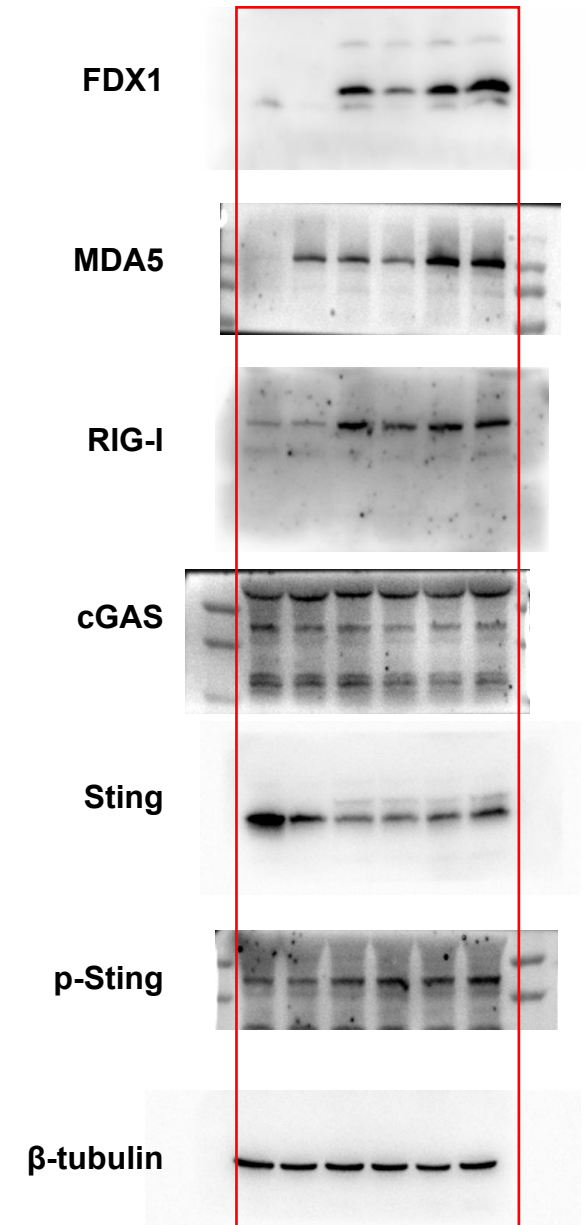

S1A

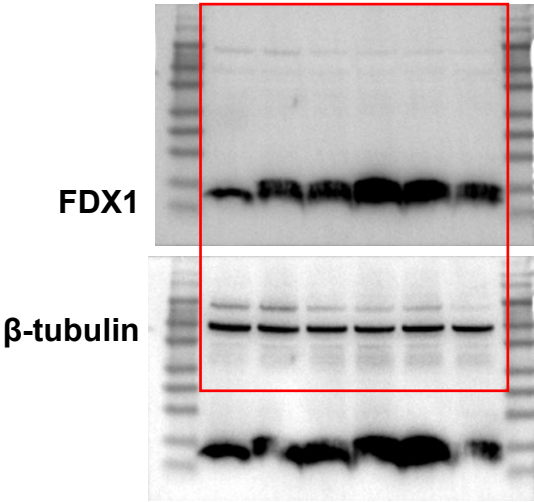

S1C

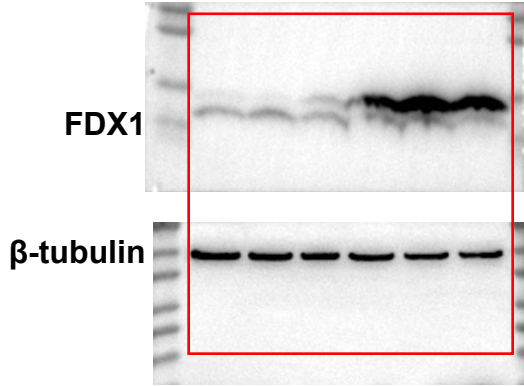

S1E

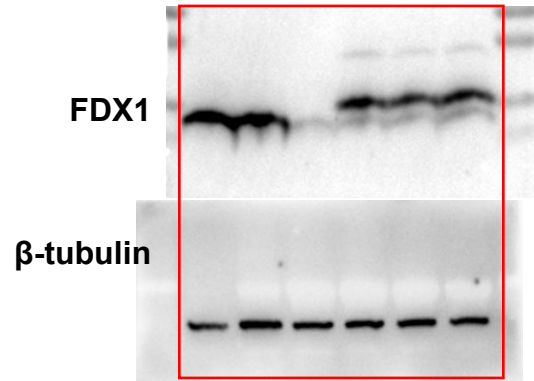

S2F

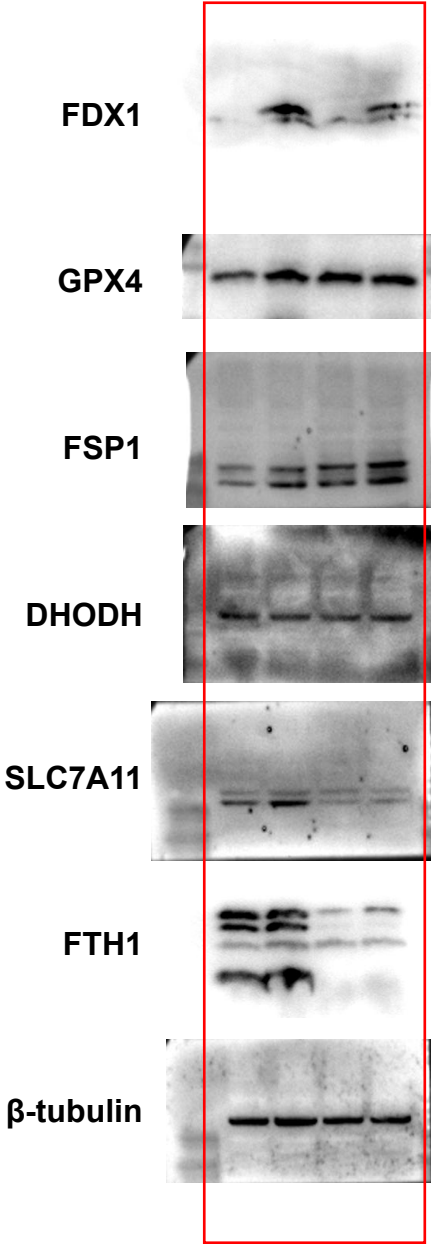

S4E

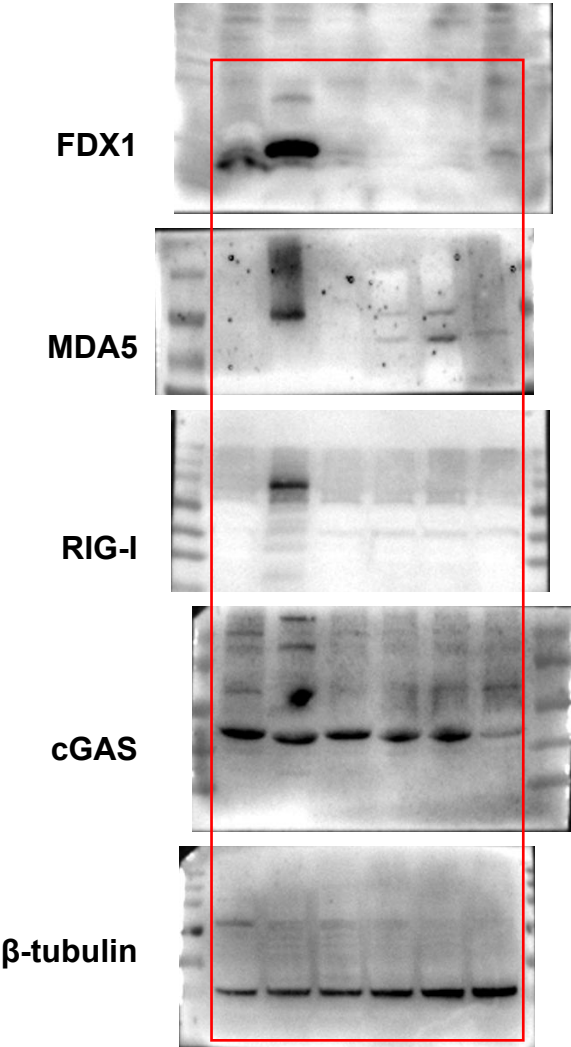

S4D

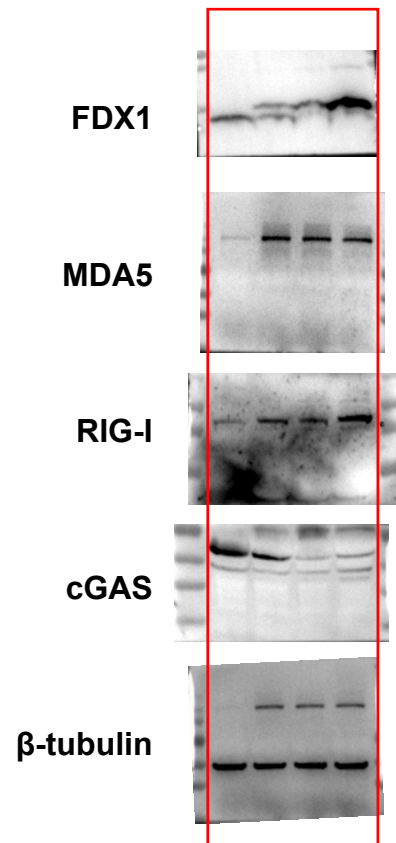

S5A

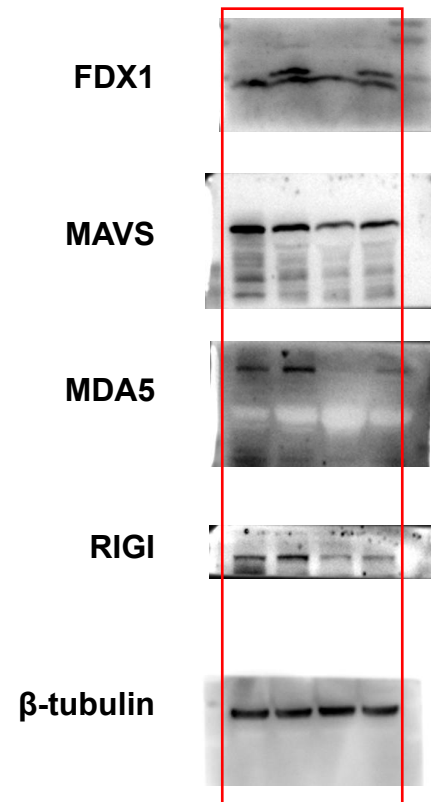

Supplement: Supplementary file 1 — Supporting Information [file ADVS-13-e18323-s005.pdf]

| STR Loci | 样品名称: PC-H2025071612 | 数据库名称: RenCa |
|----------|----------------------|--------------|
| 1-1      | 16,17,18             | 16,17,18     |
| 1-2      | 16,18                | 16,18        |
| 2-1      | 15,16,17             | 15,16,17     |
| 3-2      | 14,15                | 14,15        |
| 4-2      | 21.3                 | 21.3         |
| 5-5      | 14,15                | 14,15        |
| 6-4      | 18,19                | 18,19        |
| 6-7      | 12                   | 12           |
| 7-1      | 24.2,25.2,26.2       | 23.2,25.2    |
| 8-1      | 13                   | 13           |
| 11-2     | 17,18                | 17,18        |
| 12-1     | 16                   | 16           |
| 13-1     | 16.2,17.2            | 16.2,17.2    |
| 15-3     | 22.3,23.3            | 22.3,23.3    |
| 17-2     | 15,17                | 15,17        |
| 18-3     | 18,20                | 18,20        |
| 19-2     | 13,14                | 13,14        |
| X-1      | 25                   | 25           |
| TH01     |                      |              |
| D5S818   |                      |              |

ExPASy数据库匹配度95.52%，匹配位点数18 (<https://www.cellosaurus.org/index.html>)

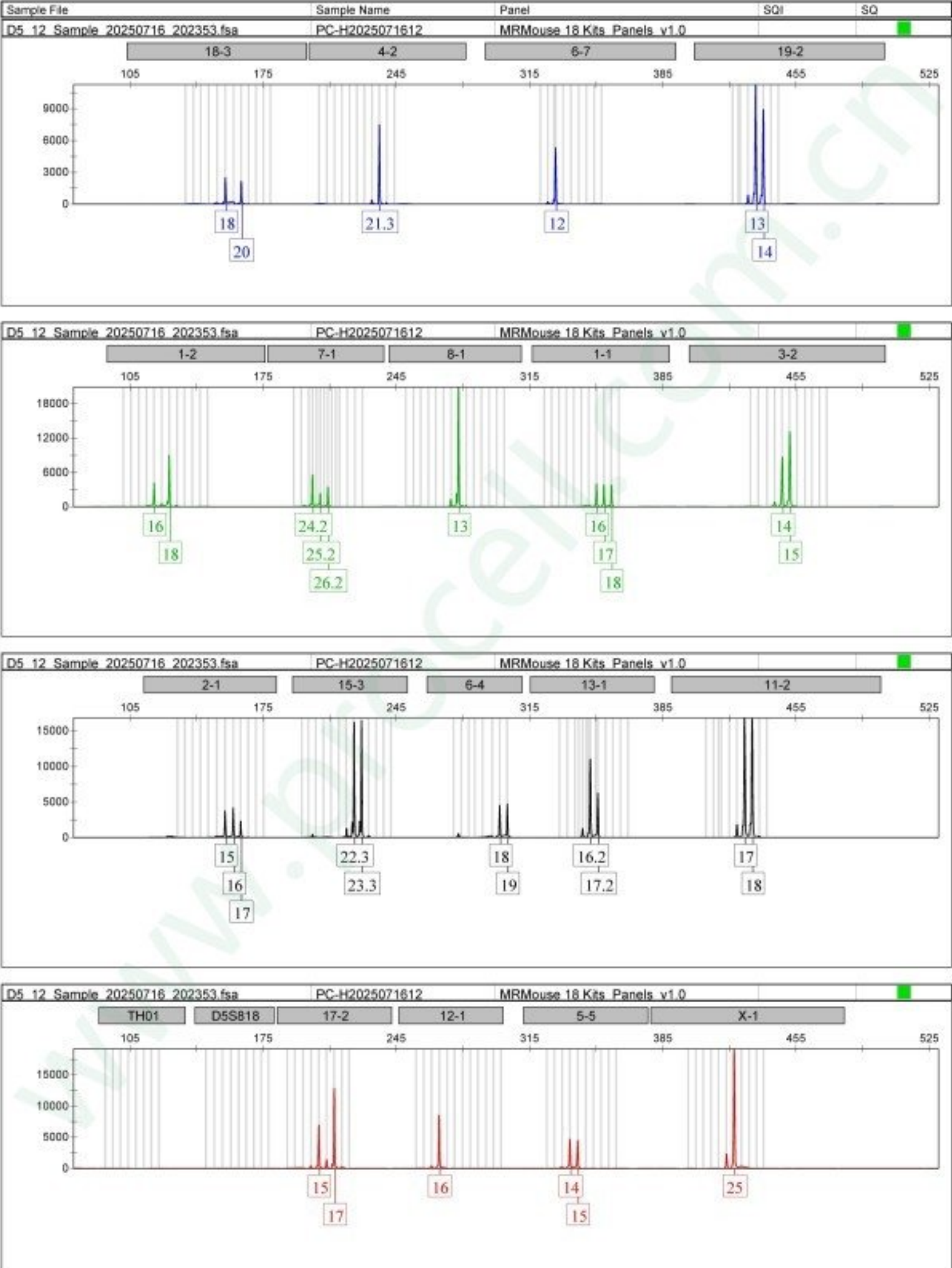

Supplement: Supplementary file 5 — Supporting Information [file ADVS-13-e18323-s001.zip › Renca STR RRID CVCL_2174.pdf]
