# Supplementary material for: Fe‐S Protein FDX1 Triggers Tumor‐Intrinsic Innate Immunity via Mitochondrial Nucleic Acids Release to Orchestrate Ferroptosis in CCRCC
Source: Adv Sci (Weinh). 2025 Nov 7;13(6):e18323. doi: 10.1002/advs.202518323 (PMC12866870; doi:10.1002/advs.202518323)
Supplement: Supplementary file 2 — Supporting Information [file ADVS-13-e18323-s002.pdf]

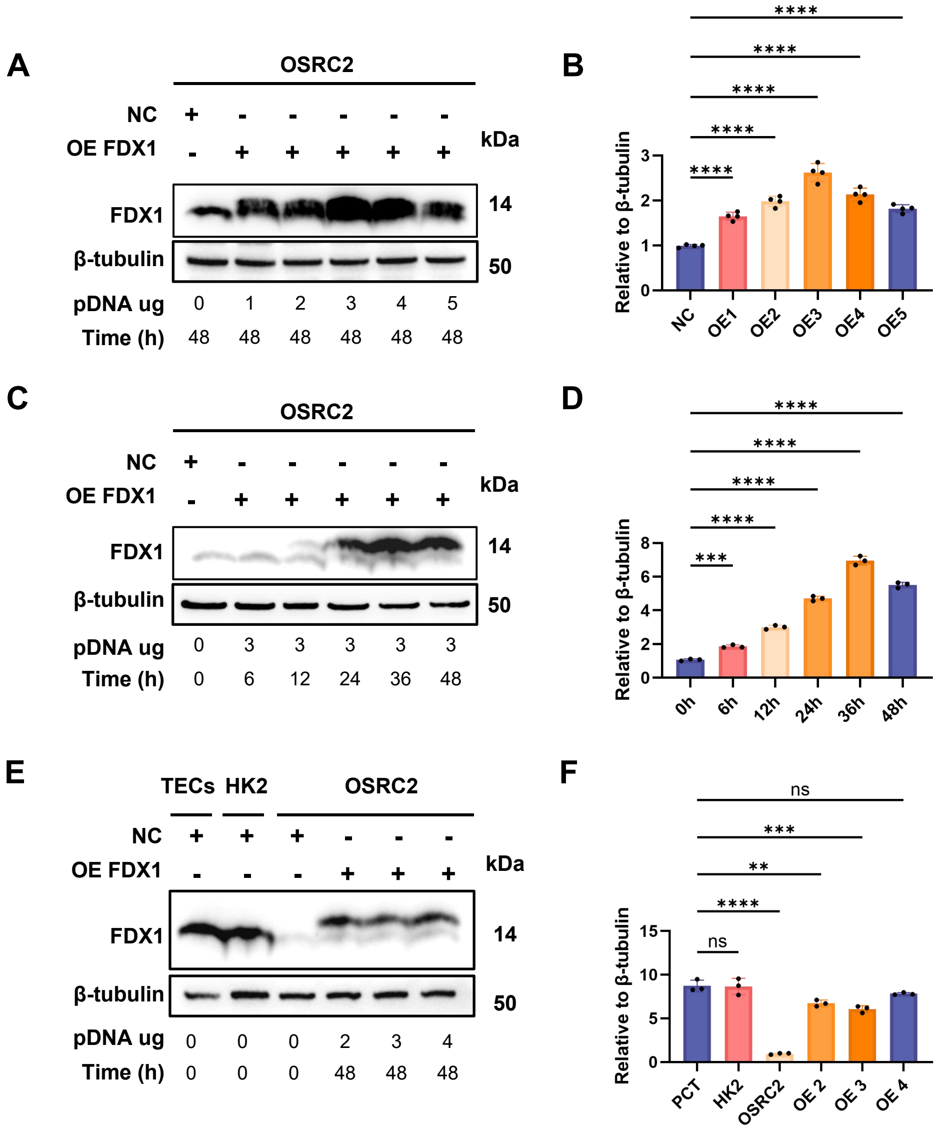

Fig.S1. Optimization of FDX1 overexpression conditions and expression efficiency in renal cell models. A and B) FDX1 expression efficiency following transient transfection with increasing concentrations of FDX1 plasmid DNA (0-5 μg) in 2 mL culture medium for 48 hours. (A) Representative immunoblot; (B) Quantification of FDX1 expression normalized to β-tubulin (loading control). C and D) Time-course analysis of FDX1 expression after transient transfection with 3 μg FDX1 plasmid DNA in 2 mL culture medium. (C) Representative immunoblot; (D) Quantification normalized to β-tubulin. E and F) Comparative FDX1 expression in OSRC2 ccRCC cells versus normal renal epithelial cells (HK2 and primary TECs) 48 hours post-transfection with indicated FDX1 plasmid DNA concentrations (0-5 μg in 2 mL medium). (E) Representative immunoblot; (F) Quantification normalized to β-tubulin. All immunoblots are representative of three independent experiments. Data are presented as mean ± SD; ns, not significant; ***p* < 0.01; ****p* < 0.001; *****p* < 0.0001, one-way ANOVA with Dunnett's multiple comparisons test (B,D and F).

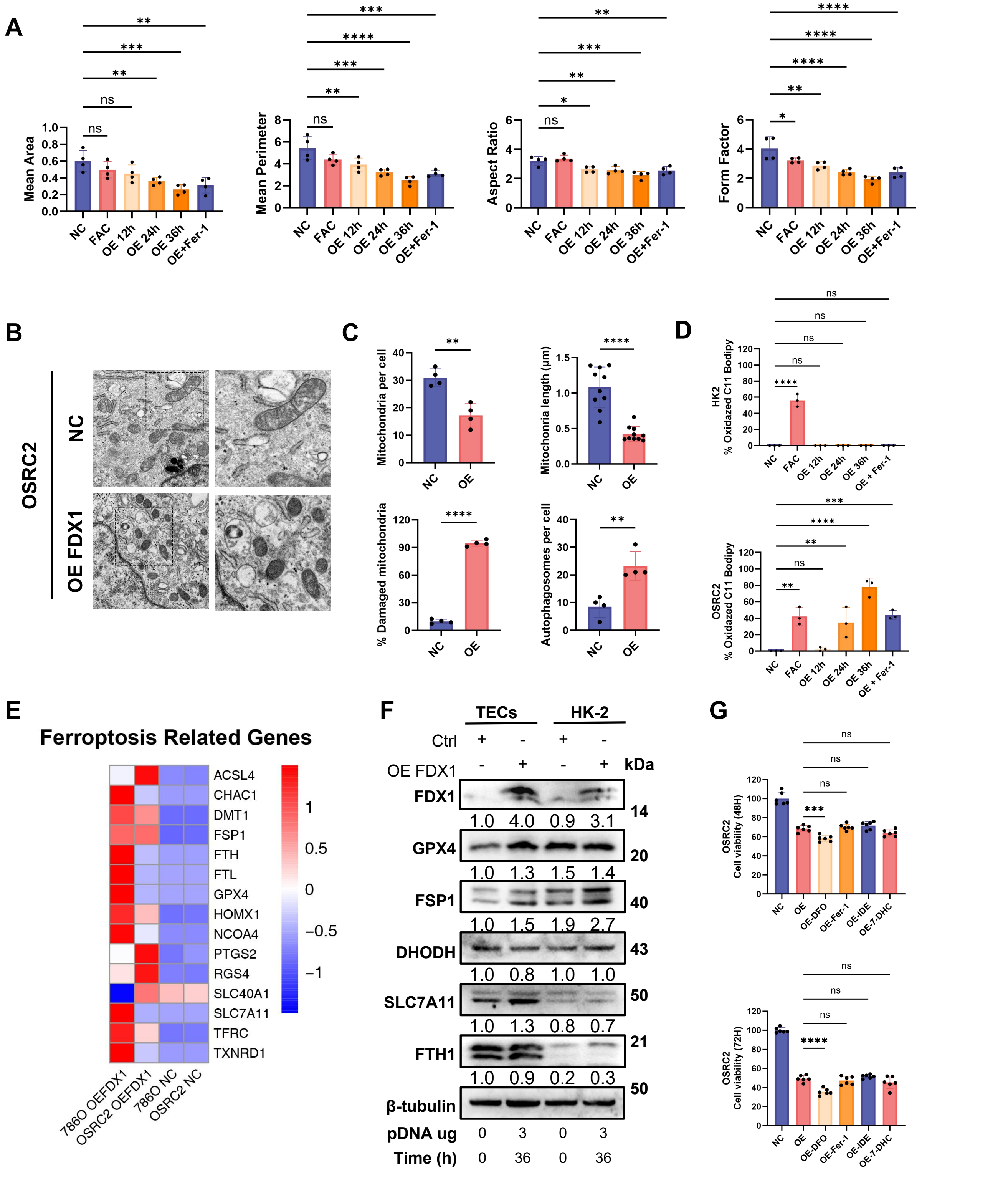

Fig.S2. Mitochondrial remodeling and ferroptosis signatures upon FDX1 overexpression. A) Quantification of mitochondrial morphology in OSRC2 cells overexpressing FDX1 at different time points (images shown in Fig. 2B). B) TEM images showing mitochondrial structure in control and FDX1-overexpressing OSRC2 cells. Scale bars: 2 µm (left), 1 µm (right). C) TEM-based quantification of mitochondrial features (n = 4). D) Lipid peroxidation levels measured by C11-BODIPY fluorescence in HK2 and OSRC2 cells. E) Heatmap of mRNA expression for iron metabolism and ferroptosis-related genes in ccRCC cells (n = 4). F) Immunoblot of ferroptosis defense proteins in primary TECs and HK2 cells with or without FDX1, normalized to β-tubulin. G) Cell viability of OE-FDX1 OSRC2 cells treated with ferroptosis inhibitors at 48 h and 72 h (n = 6). Data are presented as mean ± SD; ns, not significant; $**p < 0.01$; $***p < 0.001$; $****p < 0.0001$, Student's t-test (C) and one-way ANOVA with Dunnett's multiple comparisons test (A,D and G).

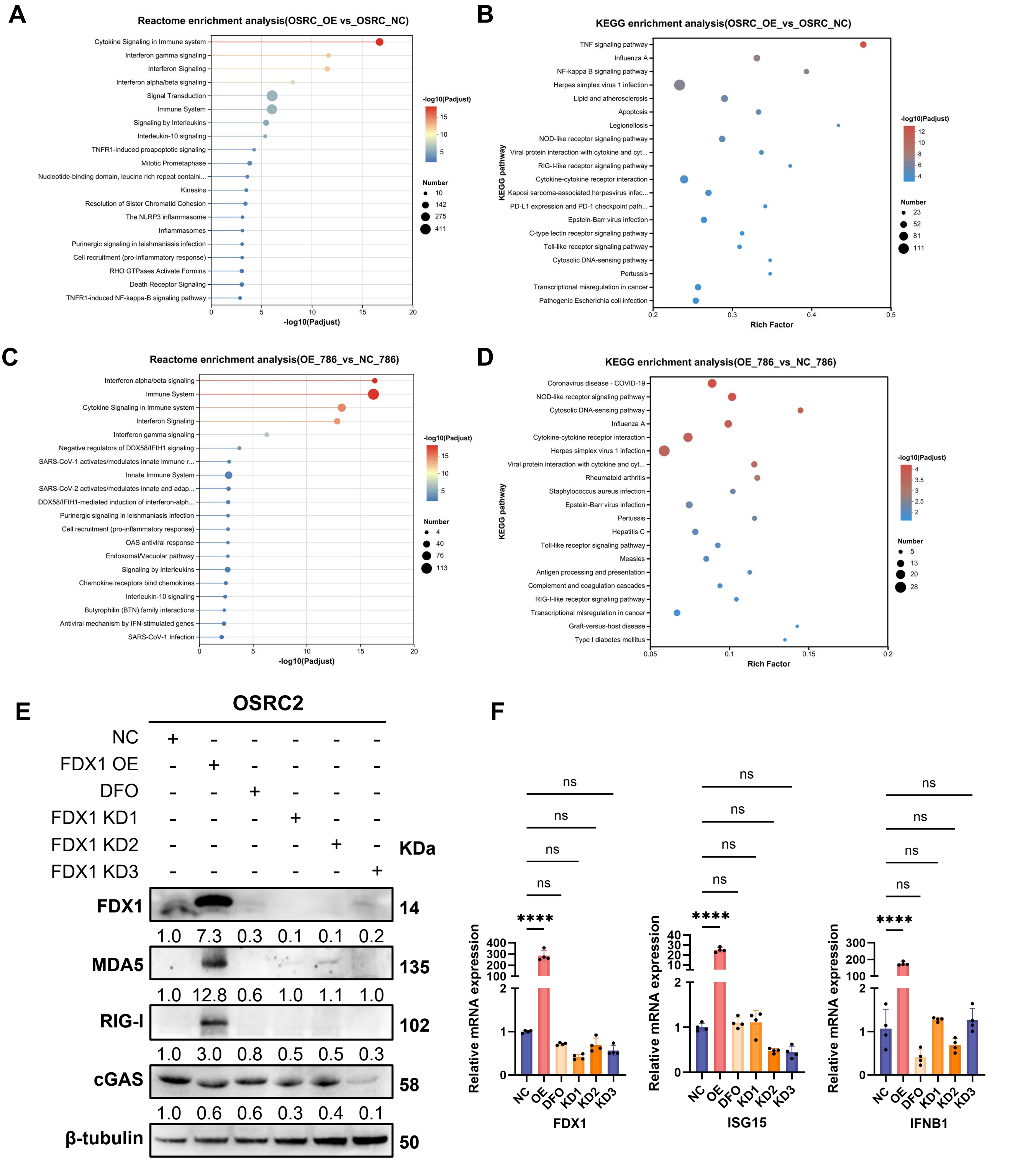

Fig.S3. Pathway enrichment and validation analysis reveal activation of immune system and interferon signaling by FDX1 in ccRCC. A and B) OSRC2 cells: (A) Reactome and (B) KEGG pathway enrichment analyses of DEGs (top 20 terms by adjusted p-value). C and D) 786-O cells: (C) Reactome and (D) KEGG pathway enrichment analyses (top 20 terms). RNA-seq analyses performed 24 hours post-transfection with FDX1 OE construct. E) Immunoblot analysis of FDX1 overexpression and FDX1 knockdown cells showing the expression levels of nucleic acid sensors. DFO (100 μM, 24 h) treatment was included as a negative control. F) Quantitative PCR analysis of downstream ISGs within the Type I interferon signaling pathway （n=4）. For all panels: y-axis = pathway terms; x-axis = rich factor (proportion of DEGs in pathway relative to background); point size = number of genes per term; point color gradient = adjusted p-value (Padjust) range (darker hues indicate greater significance). Data are presented as mean ± SD; ns, not significant; ****$p < 0.0001$, one-way ANOVA with Dunnett's multiple comparisons test (F).

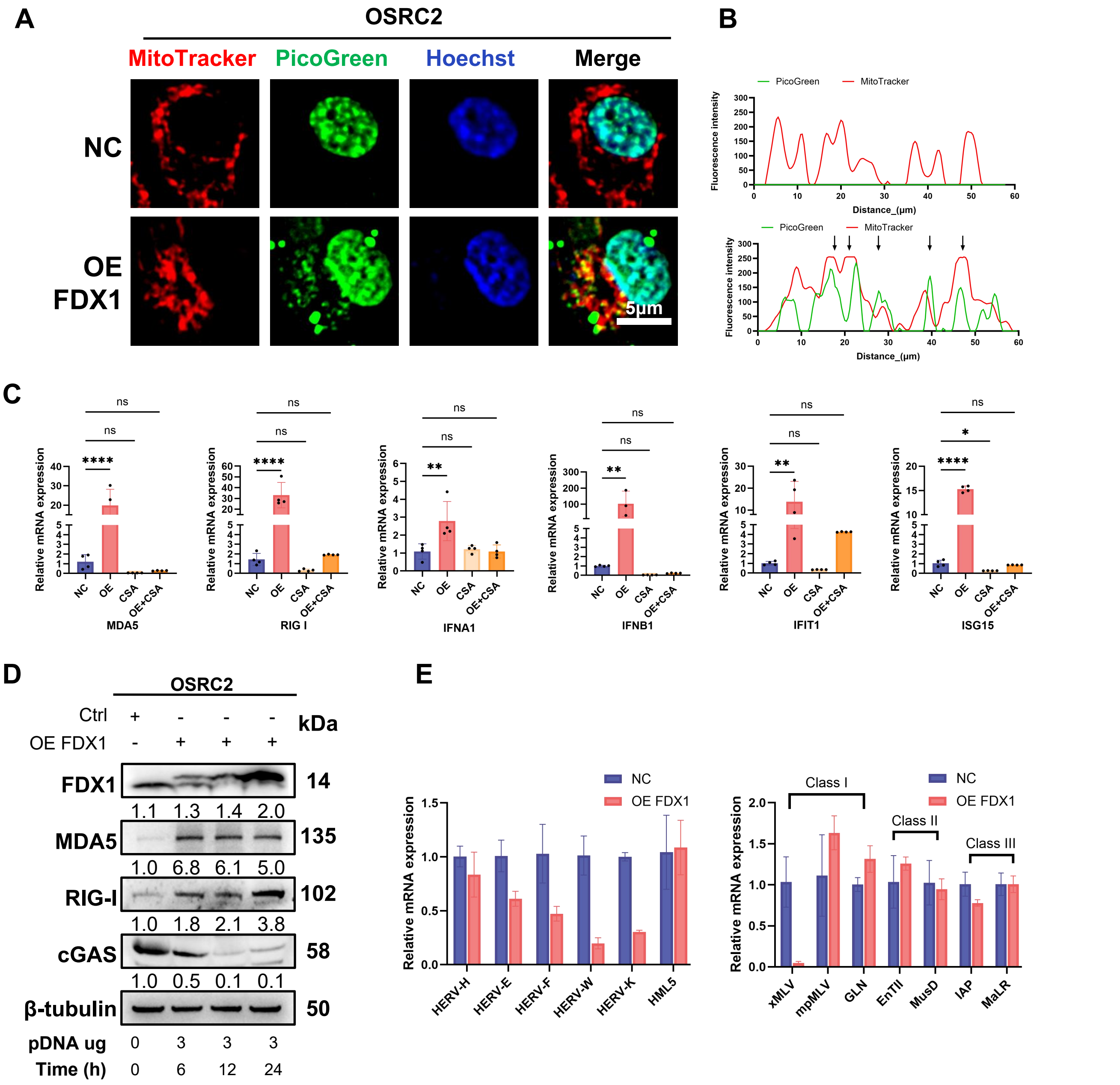

Fig.S4. Detection of FDX1-induced mtDNA release using PicoGreen and exclusion of endogenous retroviral transcripts. A) Confocal images of OSRC2 cells 12 h after FDX1 overexpression show extranuclear dsDNA stained by PicoGreen colocalizing with mitochondria. Scale bar: 5 μm. B) Fluorescence intensity profiles across selected regions. C) RT-qPCR analysis of type I interferons (IFNs) and interferon-stimulated genes (ISGs) in OSRC2 cells 24 h post-FDX1 transfection, with or without CsA 10 μM. D) Immunoblot showing time-dependent upregulation of DNA (cGAS, STING) and RNA (MDA5, RIG-I) sensors. E) RT-qPCR quantification of endogenous retroviral transcripts in human OSRC2 and murine RENCA cells. Data are presented as mean ± SD; ns, not significant; ***p* < 0.01; *****p* < 0.0001, one-way ANOVA with Dunnett's multiple comparisons test (C).

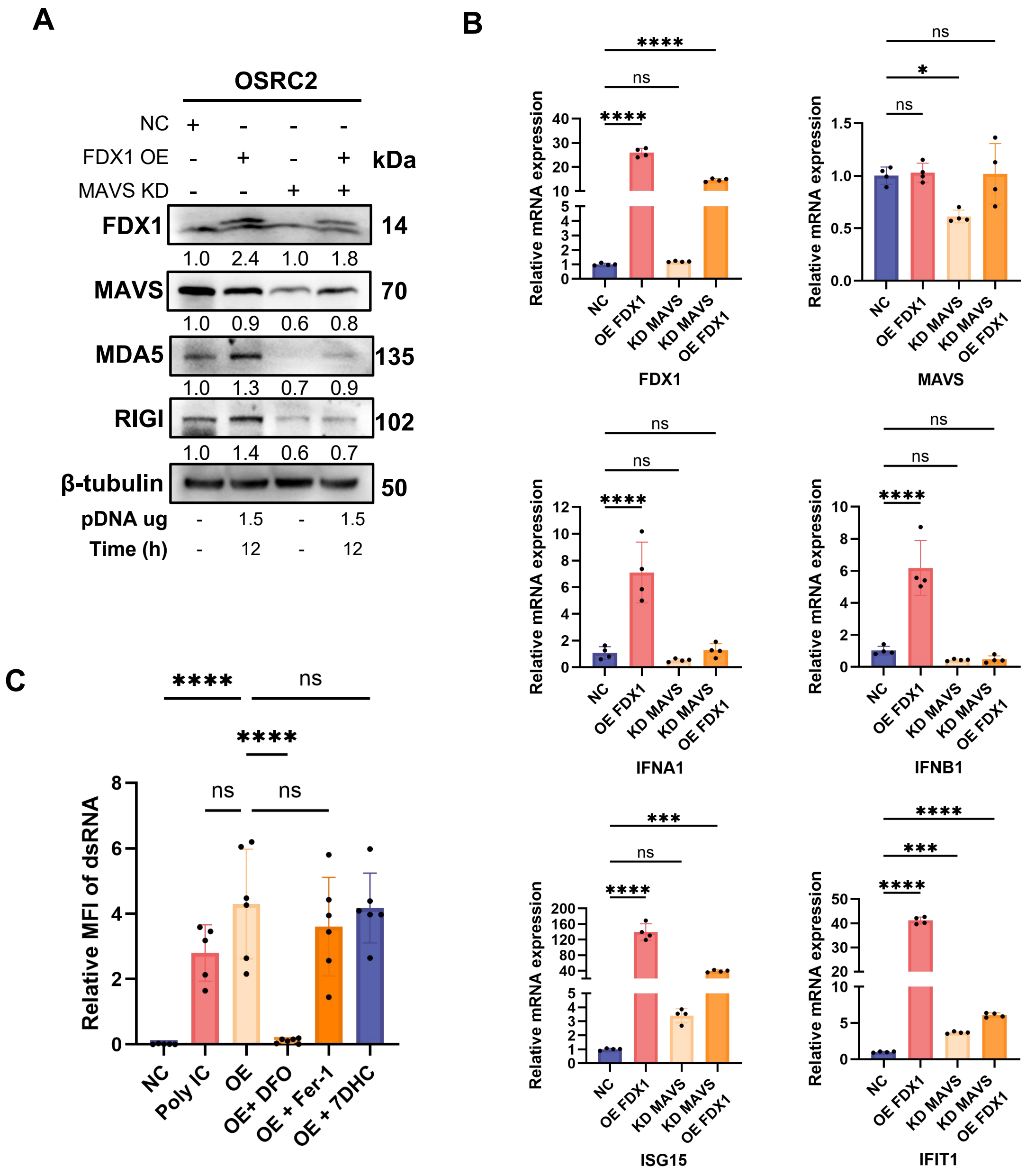

Fig.S5. FDX1-induced innate immune activation is dependent on MAVS signaling. A) Immunoblot analysis of 786-O cells transfected with FDX1 overexpression plasmid, MAVS shRNA, or both. Protein levels of MDA5, RIG-I, and MAVS were assessed after 12 h of FDX1 OE. B) qRT-PCR analysis of type I interferons and interferon-stimulated genes in the same treatment groups as in (A) (n=4). C) Quantification of cytosolic dsRNA levels by J2 immunofluorescence staining in 786-O cells. Fluorescence intensity was measured from five fields per group (n = 3). Data are presented as mean ± SD; ns, not significant; $*p < 0.05$; $***p < 0.001$; $****p < 0.0001$, Student's *t*-test (C) and one-way ANOVA with Dunnett's multiple comparisons test (B).

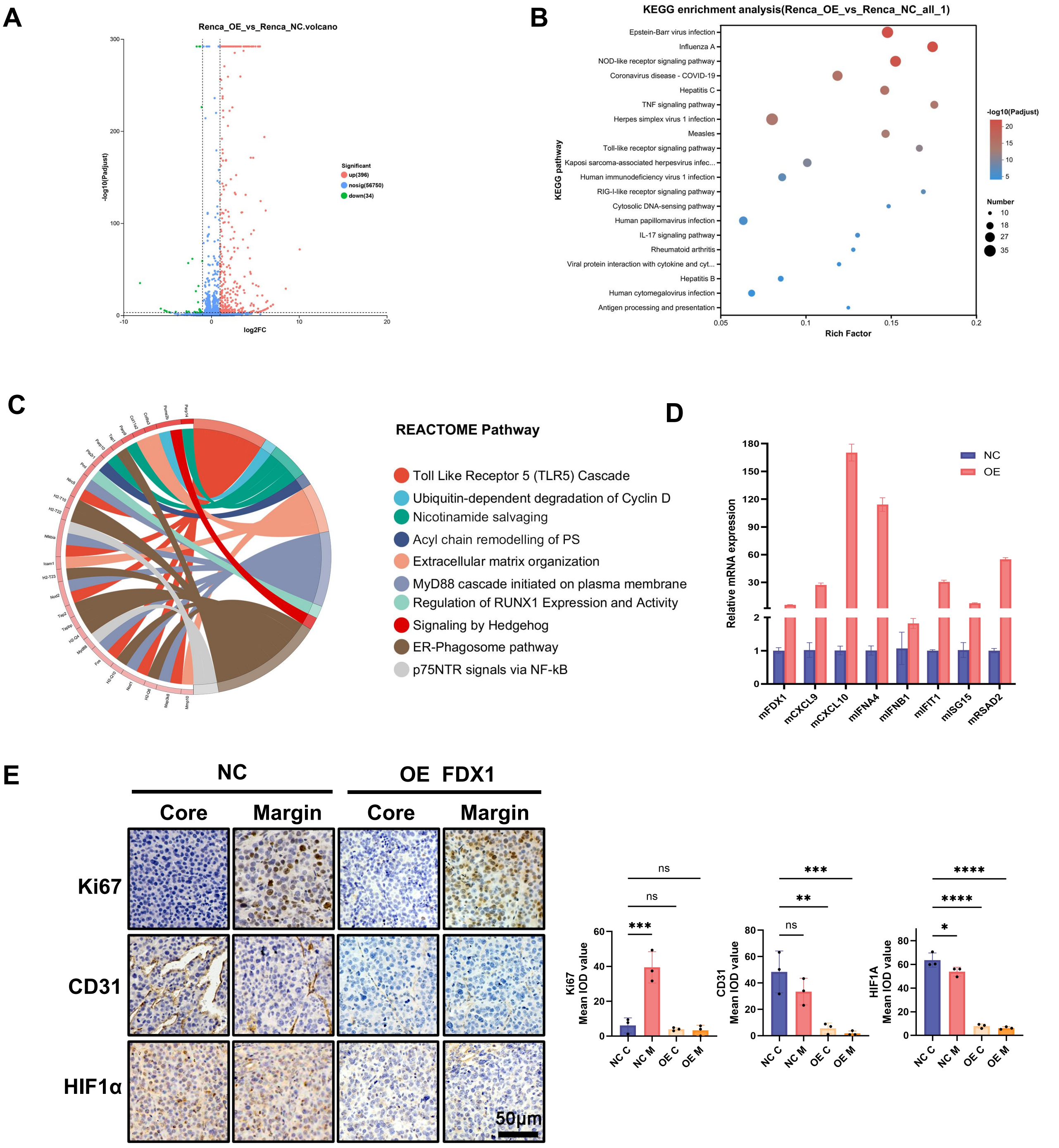

Fig.S6. Transcriptomic and histological characterization of RENCA orthotopic tumors overexpressing FDX1. A) Volcano plot showing DEGs between FDX1-overexpressing and control RENCA tumors. Red and green dots indicate significantly upregulated and downregulated genes, respectively. B) KEGG enrichment analysis of DEGs highlights top pathways, including antiviral responses and cytosolic DNA sensing. C) Reactome chord plot displays enriched immune-related pathways and associated DEGs. D) RT-qPCR validation of selected interferon-stimulated genes and CXCL chemokines in OE-FDX1 tumors. E) IHC staining for Ki-67 (proliferation), CD31 (angiogenesis), and HIF1α (hypoxia) in NC and OE-FDX1 tumor sections. Quantification is shown as integrated optical density (IOD). Scale bars: 50 μm. Data are presented as mean ± SD. ns, not significant; *$p < 0.05$; **$p < 0.01$; ***$p < 0.001$; ****$p < 0.0001$, one-way ANOVA with Dunnett's multiple comparisons test (E).
