# Supplementary material for: Fe‐S Protein FDX1 Triggers Tumor‐Intrinsic Innate Immunity via Mitochondrial Nucleic Acids Release to Orchestrate Ferroptosis in CCRCC
Source: Adv Sci (Weinh). 2025 Nov 7;13(6):e18323. doi: 10.1002/advs.202518323 (PMC12866870; doi:10.1002/advs.202518323)
Supplement: Supplementary file 3 — Supporting Information [file ADVS-13-e18323-s003.docx]

**Table S1. Primers used in the qPCR and PCR assays and shRNA for plasmid construction**

| **Genes** | **Forward Primers** | **Reverse Primers** |
| --- | --- | --- |
| **RT-qPCR primers sequence** | | |
| DMT1 | TGGAGATCATGGGGAGTCTG | AAGAAAACCTGGTCCGGTGAA |
| FSP1 | GTGAGCGGGTGAGCAATCT | CTTGATGCCGGTGCAGAGAA |
| FTL | CAGCCTGGTCAATTTGTACCT | GCCAATTCGCGGAAGAAGTG |
| FTH1 | CGAGGTGGCCGAATCTTCC | GTTTGTGCAGTTCCAGTAGTGA |
| GPX4 | GAGGCAAGACCGAAGTAAACTAC | CCGAACTGGTTACACGGGAA |
| HMOX1 | AAGACTGCGTTCCTGCTCAAC | AAAGCCCTACAGCAACTGTCG |
| NCOA4 | GAGGTGTAGTGATGCACGGAG | GACGGCTTATGCAACTGTGAA |
| SLC40A1 | TGGATGGGTTCTCACTTCCTG | GTCAATCCTTCGTATTGTGGCAT |
| SLC7A11 | TCTCCAAAGGAGGTTACCTGC | AGACTCCCCTCAGTAAAGTGAC |
| TFRC | GGCTACTTGGGCTATTGTAAAGG | CAGTTTCTCCGACAACTTTCTCT |
| TXNRD1 | ATATGGCAAGAAGGTGATGGTCC | GGGCTTGTCCTAACAAAGCTG |
| ACSL4 | CATCCCTGGAGCAGATACTCT | TCACTTAGGATTTCCCTGGTCC |
| CHAC1 | GAACCCTGGTTACCTGGGC | CGCAGCAAGTATTCAAGGTTGT |
| PTGS2 | CTGGCGCTCAGCCATACAG | CGCACTTATACTGGTCAAATCCC |
| RGS4 | ACATCGGCTAGGTTTCCTGC | GTTGTGGGAAGAATTGTGTTCAC |
| HERV-W | TGAGTCAATTCTCATACCTG | AGTTAAGAGTTCTTGGGTGG |
| HERV-H | TGGTGCCGTGACTCGGAT | GCTGAGTCCGAAAAGAGAGTC |
| HERV-E | GGTGTCACTACTCAATACAC | GCAGCCTAGGTCTCTGG |
| HERV-F | CCTCCAGTCACAACAACTC | TATTGAAGAAGGCGGCTGG |
| HERV-K | AAAGAACCAGCCACCAGG | CAGTCTGAAAACTTTTCTCTC |
| HML-5 | TGAAAGGCCAGCTTGCTG | CAATTAGGAAATTCTTTTCTAC |
| FDX1 | CTGGCTTGTTCAACCTGTCACC | GATTTGGCAGCCCAACCGTGAT |
| MAVS | CAGGCCGAGCCTATCATCTG | GGGCTTTGAGCTAGTTGGCA |
| MDA5 | GCCCGCTACATGAACCCTG | CAGCAATCCGGTTTCTGTCTT |
| RIG-I | CTGGACCCTACCTACATCCTG | GGCATCCAAAAAGCCACGG |
| IFNA1 | GCCTCGCCCTTTGCTTTACT | CTGTGGGTCTCAGGGAGATCA |
| IFNB1 | GCTTGGATTCCTACAAAGAAGCA | ATAGATGGTCAATGCGGCGTC |
| IFIT1 | TTGATGACGATGAAATGCCTGA | CAGGTCACCAGACTCCTCAC |
| ISG15 | CGCAGATCACCCAGAAGATCG | TTCGTCGCATTTGTCCACCA |
| TUBA1A | TCGATATTGAGCGTCCAACCT | CAAAGGCACGTTTGGCATACA |
| mouse-xMLV | TCTATGGTACCTGGGGCTC | GGCAGAGGTATGGTTGGAGTAG |
| mouse-mpMLV | CCGCCAGGTCCTCAATATAG | CGTCCCAGGTTGATAGAGG |
| mouse-GLN | TGTGTAAGTCCAGACGCAG | CCAACCTACTCCAAAAACAG |
| mouse-EnTII | GTGCTAACCCAACGCTGGTTC | ACTGGGGCAATCCGCCTATTC |
| mouse-MusD | GTGCTAACCCAACGCTGGTTC | CTCTGGCCTGAAACAACTCCTG |
| mouse-IAP | AAGCAGCAATCACCCACTTTGG | CAATCATTAGATGCGGCTGCCAAG |
| mouse-MaLR | ATGTTTTGGGGAGGACTGTG | AGCCCCAGCTAACCAGAAC |
| mouse-FDX1 | AGTCCACTTCAAGAACCGAGAT | GACAAGTAGAGCAAGCCAACG |
| mouse-CXCL9 | ATCTTCCTGGAGCAGTGTGG | AGTCCGGATCTAGGCAGGTT |
| mouse-CXCL10 | CGATGACGGGCCAGTGAGAATG | TCAACACGTGGGCAGGATAGGCT |
| mouse-IFNA4 | ACCCACAGCCCAGAGAGTGACC | AGGCCCTCTTGTTCCCGAGGT |
| mouse-IFNB1 | CATTACCTGAAGGCCAAGGA | CAGCATCTGCTGGTTGAAGA |
| mouse-RSAD2 | TGCTGGCTGAGAATAGCATTAGG | GCTGAGTGCTGTTCCCATCT |
| mouse-IFIT1 | CTGAGATGTCACTTCACATGGAA | GTGCATCCCCAATGGGTTCT |
| mouse-ISG15 | GGGGGAGTATGGCCTAAAGC | CCAACACTGGCTCTGGATGG |
| mouse-GAPDH | AGGTCGGTGTGAACGGATTTG | TGTAGACCATGTAGTTGAGGTCA |
| **PCR primers sequence** | | |
| ND1 | CGGGCTACTACAACCCTTCG | GCGATGGTGAGAGCTAAGGT |
| ND2 | CTATCACCCTATTAACCACTCA | TTCGCCTGTAATATTGAACGTA |
| ND4 | CAGCCACATAGCCCTCGTAG | CCCGTGGGCGATTATGAGAA |
| D-loop | AATCTACCATCCTCCGTGAAACC | TCAGTTTAGCTACCCCCAAGTTTAA |
| 18S | TAGAGGGACAAGTGGCGTTC | CGCTGAGCCAGTCAGTGT |
| **shRNA Oligo Sequence** | | |
| MAVS shRNA | CCGGGATGTGGATGTTGTAGAGATTCCTCGAGGAATCTCTACAACATCCACATTTTTTG | |
| FDX1 shRNA1 | CCGGGCAATCACTGATGAGGAGAATCTCGAGATTCTCCTCATCAGTGATTGCTTTTTG | |
| FDX1 shRNA2 | CCGGTGGTGAAACATTAACAACCAACTCGAGTTGGTTGTTAATGTTTCACCATTTTTG | |
| FDX1 shRNA3 | CCGGGATGCCAGACAATCCATTGATCTCGAGATCAATGGATTGTCTGGCATCTTTTTG | |
