# Supplementary material for: Fe‐S Protein FDX1 Triggers Tumor‐Intrinsic Innate Immunity via Mitochondrial Nucleic Acids Release to Orchestrate Ferroptosis in CCRCC
Source: Adv Sci (Weinh). 2025 Nov 7;13(6):e18323. doi: 10.1002/advs.202518323 (PMC12866870; doi:10.1002/advs.202518323)
Supplement: Supplementary file 4 — Supporting Information [file ADVS-13-e18323-s004.docx]

**Table S2. Antibodies used in this study for immunoblotting, immunofluorescence and immunohistochemistry**

| **Antibody target** | **Vendor** | **Catalog No.** | **Host** | **Dilution** |
| --- | --- | --- | --- | --- |
| **FDX1** | Abcam | ab108257 | Rabbit mAb | 1:1000 |
| **LC3B** | Abcam | ab192890 | Rabbit mAb | 1:2000 |
| **TOMM20** | proteintech | 11802-1-AP | Rabbit mAb | 1:1000 |
| **β-Tubulin** | Easybio | BE0025 | mouse mAb | 1:2000 |
| **GPX4** | CST | 52455 | Rabbit mAb | 1:1000 |
| **AIFM2/ FSP1** | proteintech | 20886-1-AP | Rabbit pAb | 1:5000 |
| **DHODH (E-8)** | Santa Cruz | sc-166348 | Mouse mAb | 1:500 |
| **SLC7A11/xCT** | proteintech | 26864-1-AP | Rabbit pAb | 1:1000 |
| **FTH1** | Abcam | ab75973 | Rabbit pAb | 1:2000 |
| **P62** | proteintech | 18420-1-AP | Rabbit pAb | 1:1000 |
| **cGAS** | proteintech | 26416-1-AP | Rabbit pAb | 1:1000 |
| **MDA5** | CST | 5321 | Rabbit mAb | 1:1000 |
| **RIG-I** | CST | 3743 | Rabbit mAb | 1:1000 |
| **STING** | proteintech | 19851-1-AP | Rabbit pAb | 1:1000 |
| **Phospho-STING** | CST | 50907 | Rabbit mAb | 1:1000 |
| **TBK1** | MCE | YA662 | Mouse mAb | 1:500 |
| **Phospho-TBK1** | CST | 5483 | Rabbit mAb | 1:1000 |
| **MAVS** | CST | 24930 | Rabbit mAb | 1:1000 |
| **rJ2** | Sigma | MABE1134 | Mouse mAb | 1:200 |
| **HIF-1α** | Abcam | ab237544 | Rabbit pAb | 1:400 |
| **Ki67** | proteintech | 28074-1-AP | Rabbit pAb | 1:400 |
| **CD31** | proteintech | 28083-1-AP | Rabbit pAb | 1:400 |
| **PAX8** | proteintech | 10336-1-AP | Rabbit pAb | 1:400 |
| **CD4** | Abcam | ab183685 | Rabbit mAb | 1:400 |
| **CD8** | Abcam | ab217344 | Rabbit mAb | 1:400 |
