# Supplementary material for: Fe‐S Protein FDX1 Triggers Tumor‐Intrinsic Innate Immunity via Mitochondrial Nucleic Acids Release to Orchestrate Ferroptosis in CCRCC
Source: Adv Sci (Weinh). 2025 Nov 7;13(6):e18323. doi: 10.1002/advs.202518323 (PMC12866870; doi:10.1002/advs.202518323)
Supplement: Supplementary file 5 — Supporting Information [file ADVS-13-e18323-s001.zip › 786O STR RRID CVCL_1051.pdf]

STR Loci	样品名称：PC-H2025082801	数据库名称：786-O
Amelogenin	X, Y	X,Y
CSF1PO	10	10
D2S1338	17,18	17,18
D3S1358	16	16
D5S818	9	9
D7S820	11,12	11,12
D8S1179	13	13
D13S317	8	8
D16S539	12	12
D18S51	13,14	13,14
D19S433	14,15	14,15
D21S11	29,30	29,30
FGA	24	24
Penta D	9,12	9,12
Penta E	7,16	7,16
TH01	6,9.3	6,9.3
TPOX	8,11	8,11
vWA	15,17	15,17
D6S1043	11	
D12S391	17,18	
D2S441	10,11	
ExPASy数据库匹配度100.00%，匹配位点数17（https://www.cellosaurus.org/index.html）		

普诺赛® | Procell system
普诺赛® | Procell system

Applied Biosystems

GeneMapper Software 6

20250828

Sample File	Sample Name	Panel	SQI	SQ
A3_1_Sample_20250828_171507.fsa	PC-H2025082801	MR21_v2.1		

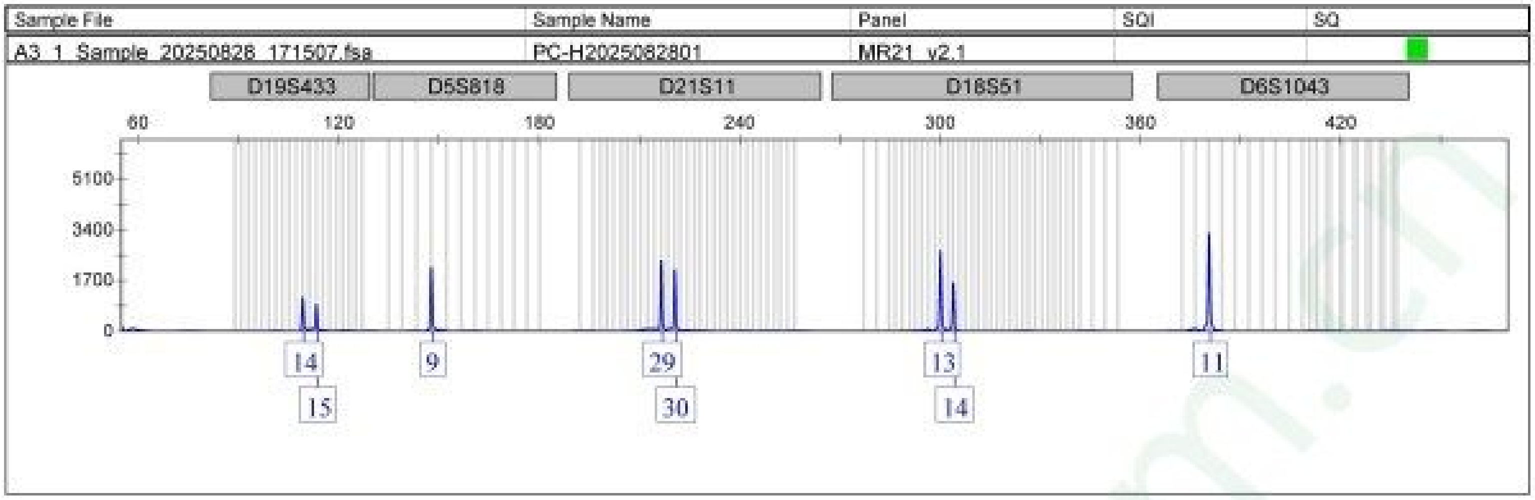

Sample File	Sample Name	Panel	SQI	SQ
A3_1_Sample_20250828_171507.fsa	PC-H2025082801	MR21_v2.1		

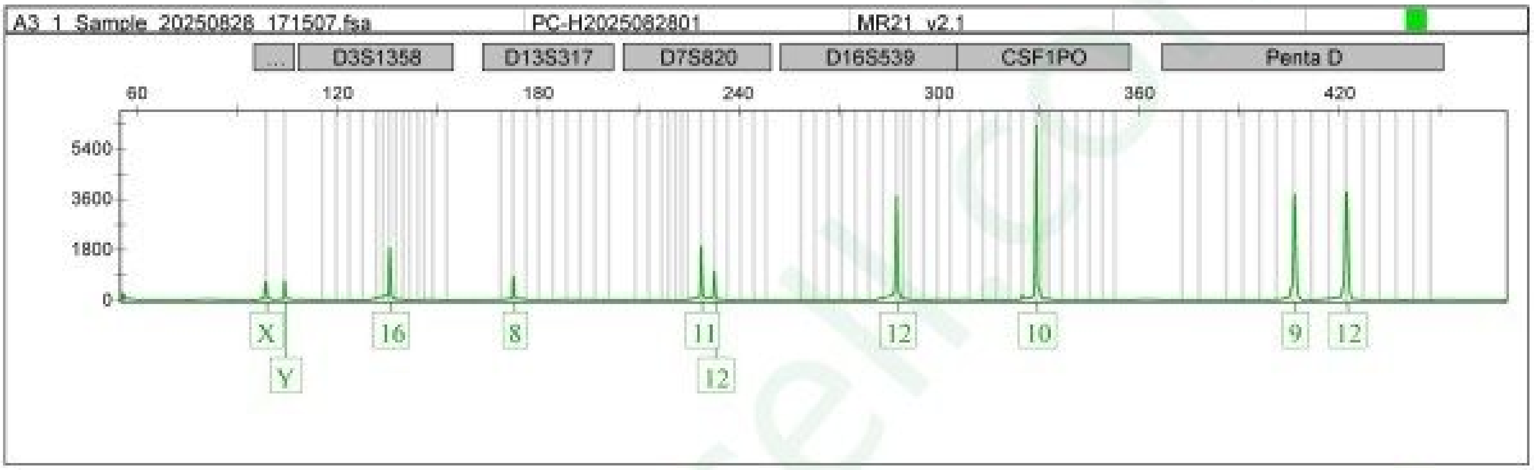

Sample File	Sample Name	Panel	SQI	SQ
A3_1_Sample_20250828_171507.fsa	PC-H2025082801	MR21_v2.1		

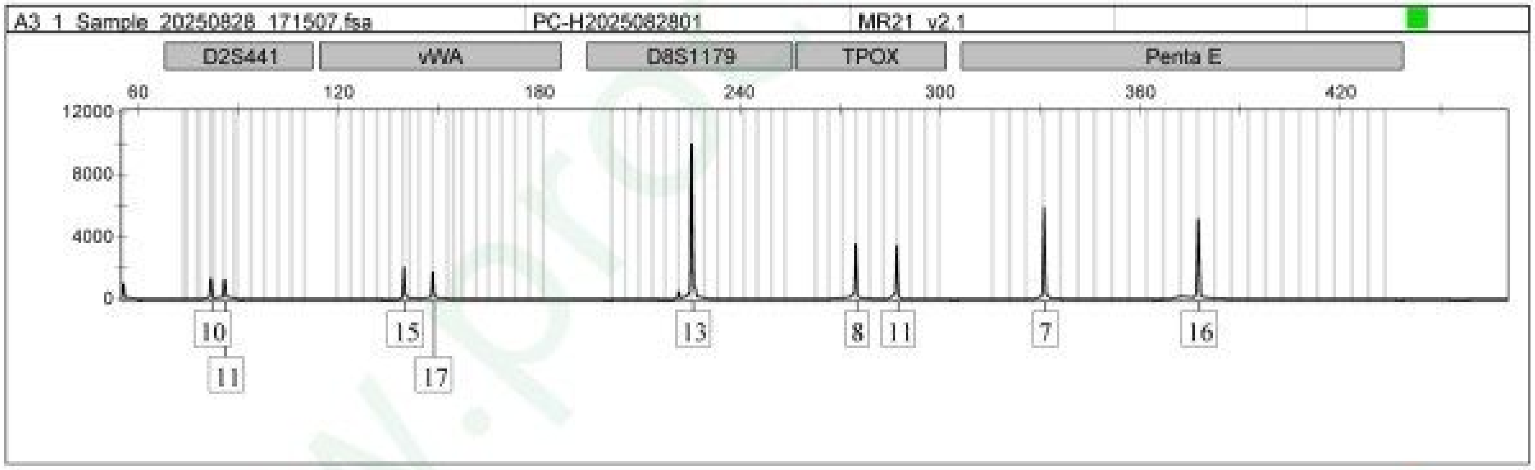

Sample File	Sample Name	Panel	SQI	SQ
A3_1_Sample_20250828_171507.fsa	PC-H2025082801	MR21_v2.1		

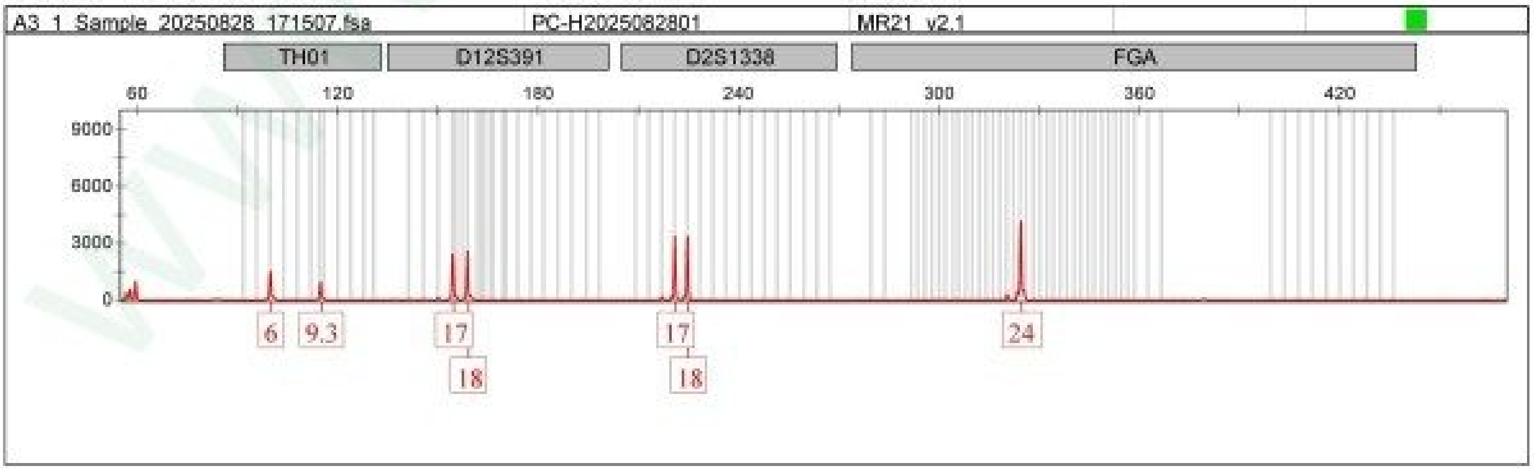

www.procell.com.cn
