# Supplementary material for: Fe‐S Protein FDX1 Triggers Tumor‐Intrinsic Innate Immunity via Mitochondrial Nucleic Acids Release to Orchestrate Ferroptosis in CCRCC
Source: Adv Sci (Weinh). 2025 Nov 7;13(6):e18323. doi: 10.1002/advs.202518323 (PMC12866870; doi:10.1002/advs.202518323)

STR Loci	样品名称：PC-H2025090303	数据库名称：HEK293T
Amelogenin	X	X
CSF1PO	11,12	11,12
D2S1338	19	19
D3S1358	15,16,17	15,16,17
D5S818	8,9	8,9
D7S820	11	11
D8S1179	11,12,14	12,14
D13S317	12,14	12,14
D16S539	9,13	9,13
D18S51	17,18	17,18
D19S433	18	18
D21S11	28,30.2	28,30.2
FGA	23	23
Penta D	9,10	9,10
Penta E	7,15	7,15
TH01	7,9.3	7,9.3
TPOX	11	11
vWA	16,19	16,19
D6S1043	11	
D12S391	19,21	19,21
D2S441	11,15	11,15
ExPASy数据库匹配度98.55%，匹配位点数19（https://www.cellosaurus.org/index.html）		

普诺赛® | Procell system
普诺赛® | Procell system

Applied Biosystems

GeneMapper Software 6

20250903

Sample File	Sample Name	Panel	SQI	SQ
C2_3_Sample_20250904_151911.fsa	PC-H2025090303	MR21_v2.1		

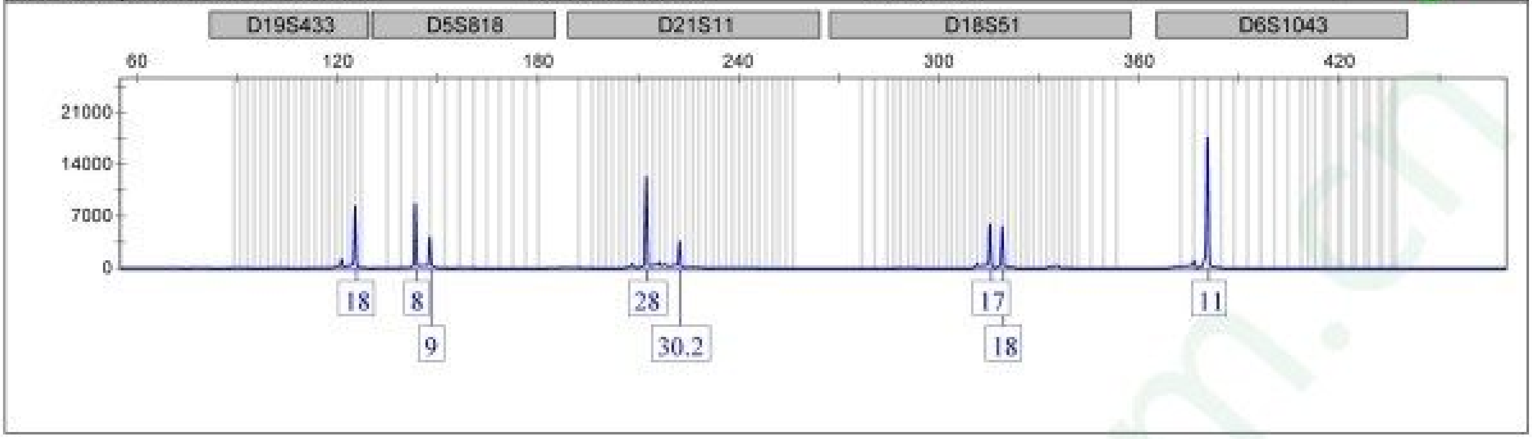

Sample File	Sample Name	Panel	SQI	SQ
C2_3_Sample_20250904_151911.fsa	PC-H2025090303	MR21_v2.1		

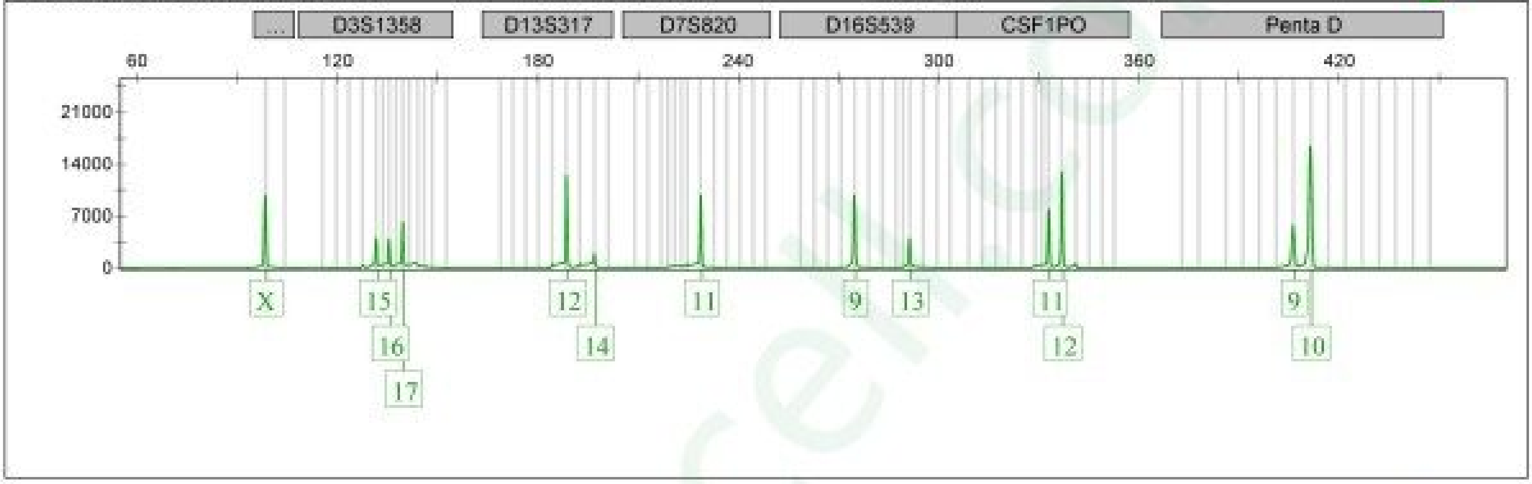

Sample File	Sample Name	Panel	SQI	SQ
C2_3_Sample_20250904_151911.fsa	PC-H2025090303	MR21_v2.1		

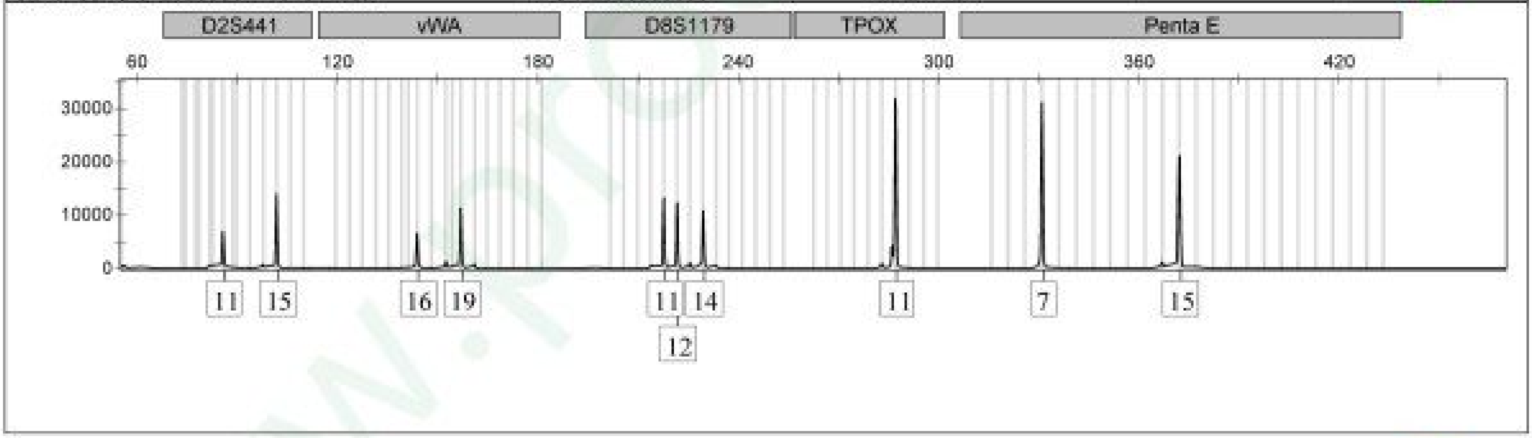

Sample File	Sample Name	Panel	SQI	SQ
C2_3_Sample_20250904_151911.fsa	PC-H2025090303	MR21_v2.1		

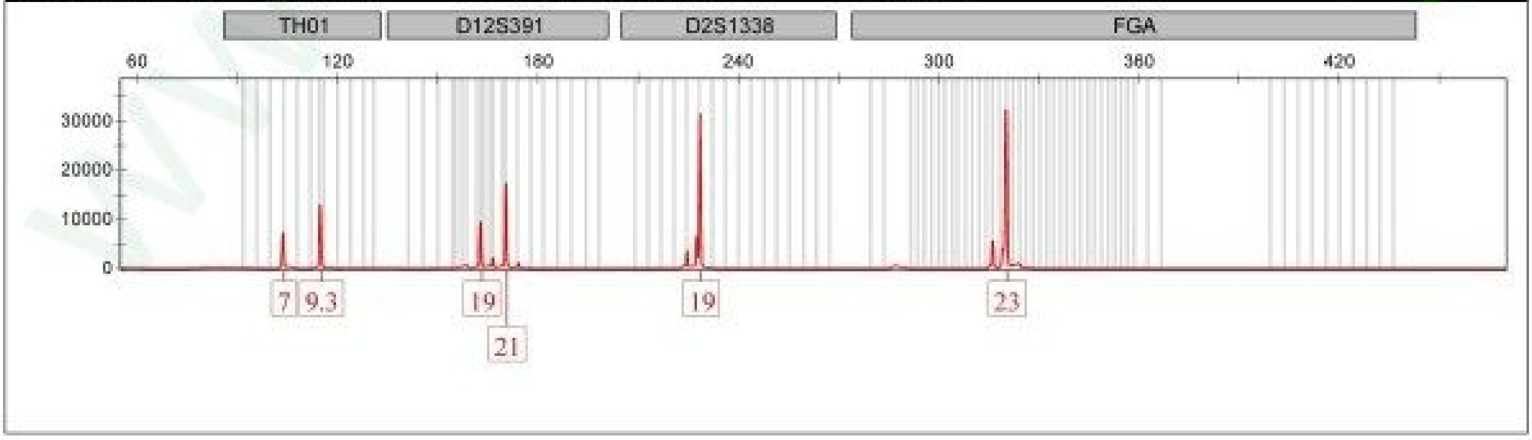

Supplement: Supplementary file 5 — Supporting Information [file ADVS-13-e18323-s001.zip › HEK293T STR RRID CVCL_0045.pdf]
