# Supplementary material for: Fe‐S Protein FDX1 Triggers Tumor‐Intrinsic Innate Immunity via Mitochondrial Nucleic Acids Release to Orchestrate Ferroptosis in CCRCC
Source: Adv Sci (Weinh). 2025 Nov 7;13(6):e18323. doi: 10.1002/advs.202518323 (PMC12866870; doi:10.1002/advs.202518323)
Supplement: Supplementary file 5 — Supporting Information [file ADVS-13-e18323-s001.zip › HK-2 STR RRID CVCL_0302.pdf]

STR Loci	样品名称：PC-H2025082834	数据库名称：HK-2 [Human kidney]
Amelogenin	X	X
CSF1PO	13	13
D2S1338	17,25	17,25
D3S1358	16	16,17
D5S818	12	12
D7S820	10,11	10,11
D8S1179	10,14	10,14
D13S317	9	9
D16S539	12	11,12
D18S51	12	12
D19S433	15,15.2	15,15.2
D21S11	28,30	28,30
FGA	20	20,22
Penta D	9,12	9,12
Penta E	10,11	10,11
TH01	9	9
TPOX	8,9	8,9
vWA	17,18	17,18
D6S1043	12,13	
D12S391	17.3,22	
D2S441	11,12	
ExPASy数据库匹配度94.55%，匹配位点数17（https://www.cellosaurus.org/index.html）		

普诺赛® | Procell system

Sample File	Sample Name	Panel	SQI	SQ
H7_34_Sample_20250828_225877.fsa	PC-H2025082834	MR21_v2.1		

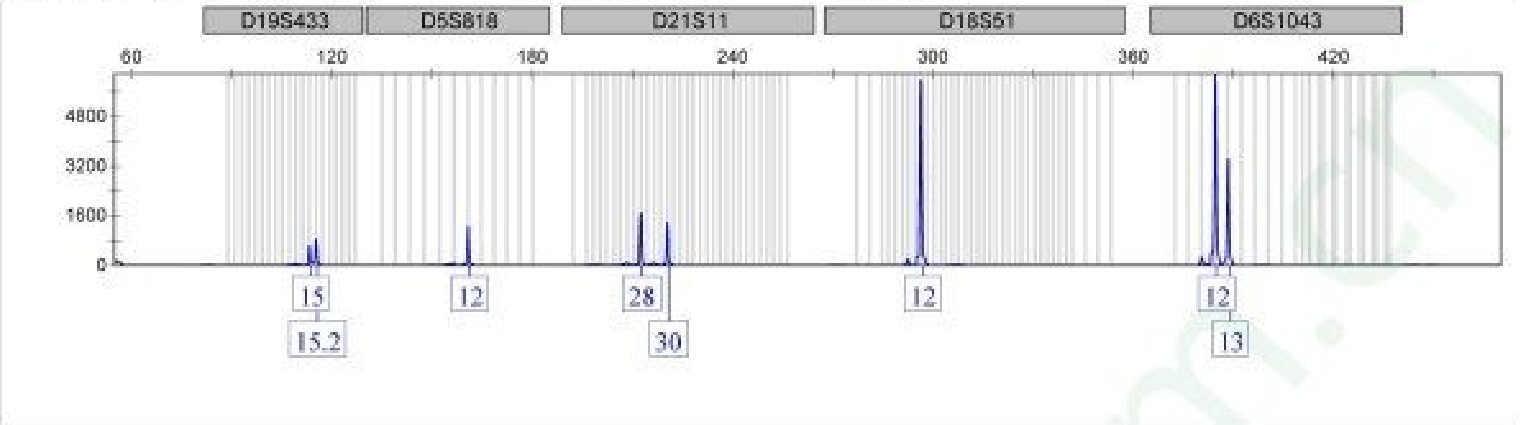

H7_34_Sample_20250828_225877.fsa	PC-H2025082834	MR21_v2.1		

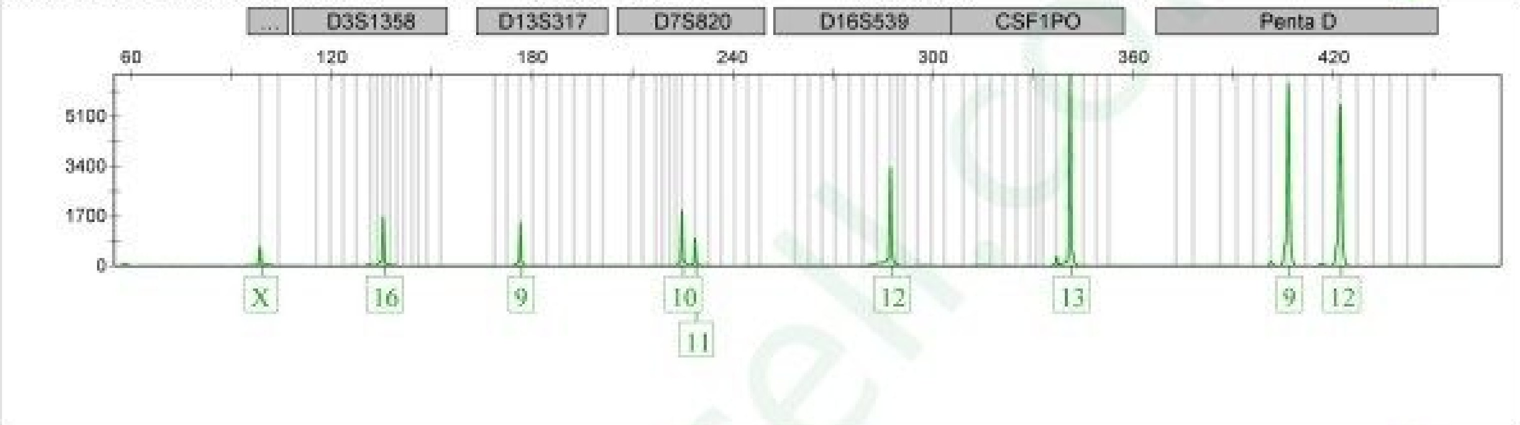

H7_34_Sample_20250828_225877.fsa	PC-H2025082834	MR21_v2.1		

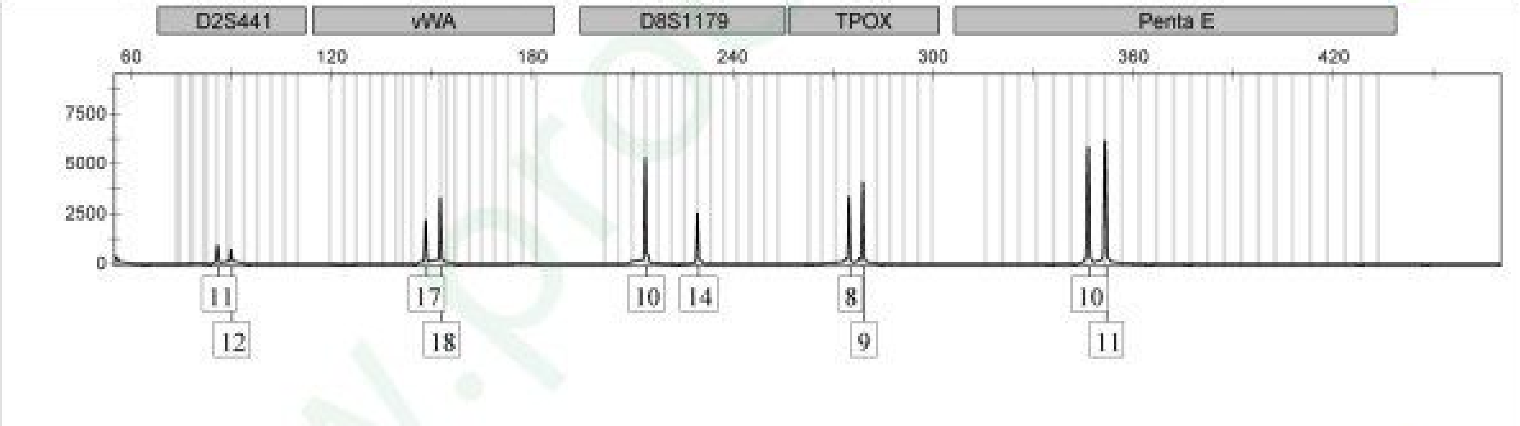

H7_34_Sample_20250828_225877.fsa	PC-H2025082834	MR21_v2.1		

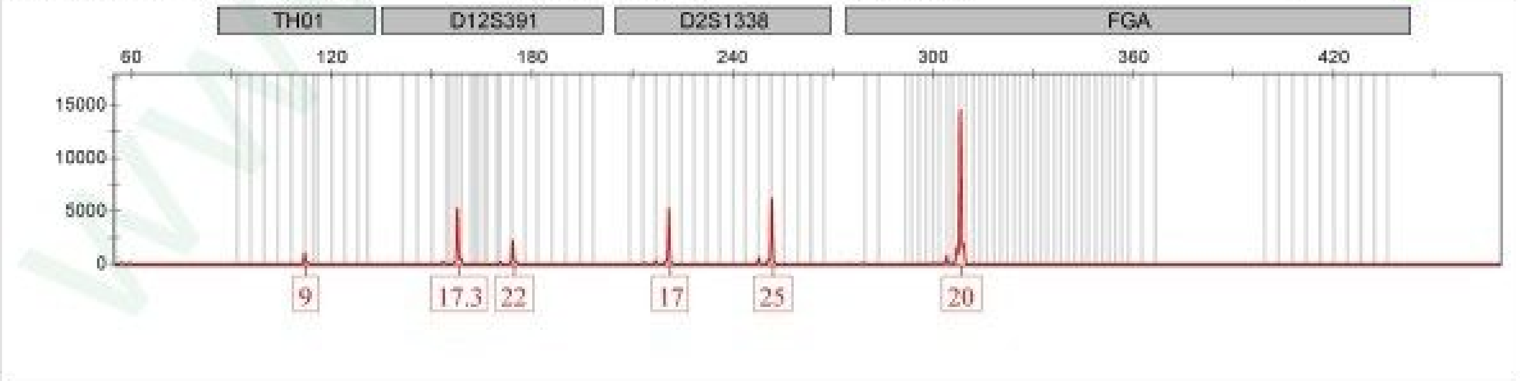

www.procell.com.cn
