# Supplementary material for: Fe‐S Protein FDX1 Triggers Tumor‐Intrinsic Innate Immunity via Mitochondrial Nucleic Acids Release to Orchestrate Ferroptosis in CCRCC
Source: Adv Sci (Weinh). 2025 Nov 7;13(6):e18323. doi: 10.1002/advs.202518323 (PMC12866870; doi:10.1002/advs.202518323)
Supplement: Supplementary file 5 — Supporting Information [file ADVS-13-e18323-s001.zip › OS-RC-2 STR RRID CVCL_1626.pdf]

STR Loci	样品名称：PC-H2025062545	数据库名称：OS-RC-2
Amelogenin	X, Y	X,Y
CSF1PO	12,13	12,13
D2S1338	20	20
D3S1358	17	17
D5S818	11,12	11,12
D7S820	10,11	10,11
D8S1179	14	14
D13S317	8,12	8,12
D16S539	11	11
D18S51	14	14
D19S433	13,14	13,14
D21S11	31.2	31.2
FGA	24	24
Penta D	11	
Penta E	11,17	
TH01	6	6
TPOX	8,11	8,11
vWA	16	16
D6S1043	12	
D12S391	20,23	
D2S441	11	
ExPASy数据库匹配度100.00%，匹配位点数15（https://www.cellosaurus.org/index.html）		

普诺赛® Procell system

Sample File	Sample Name	Panel	SQI	SQ
F7_45_Sample_20250625_213352.fsa	PC-H2025062545	MR21_v2.1		

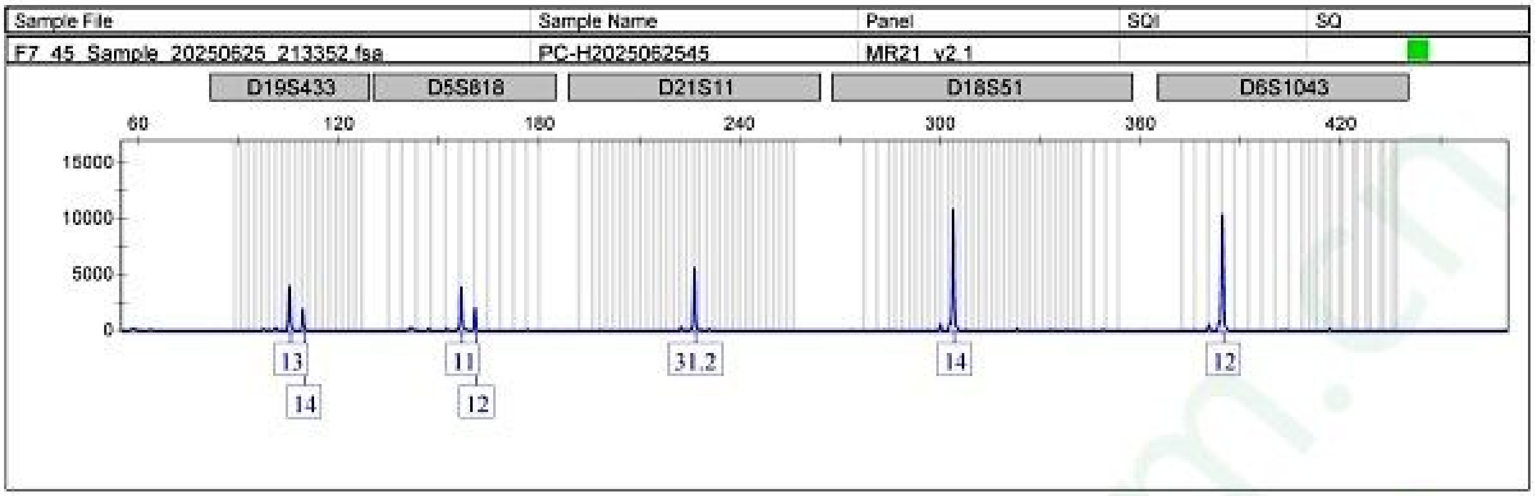

F7_45_Sample_20250625_213352.fsa	PC-H2025062545	MR21_v2.1

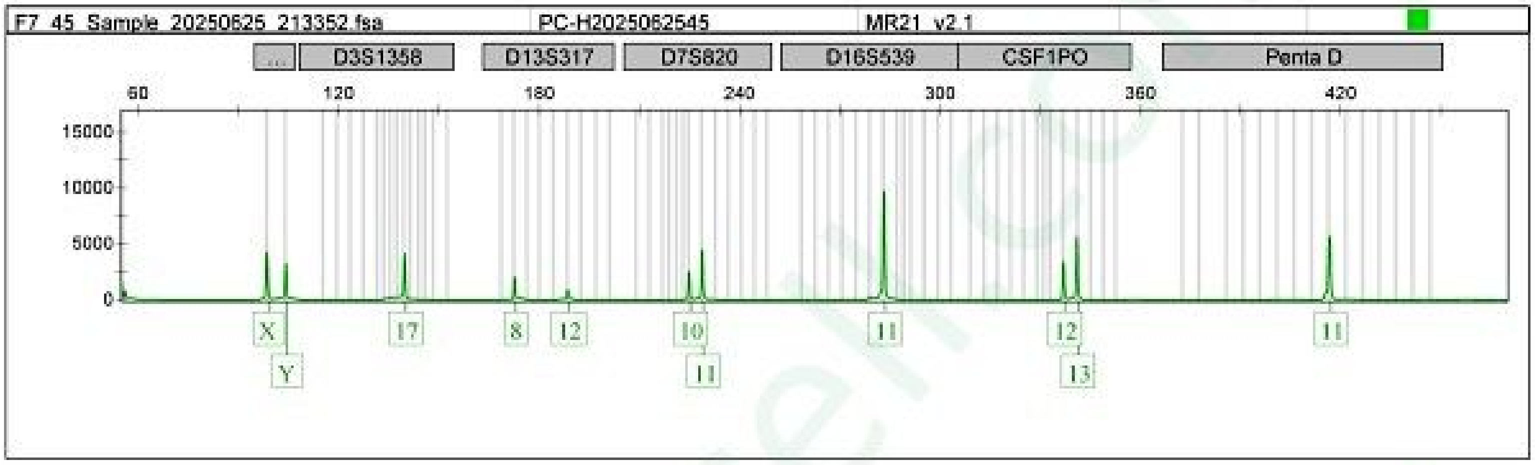

F7_45_Sample_20250625_213352.fsa	PC-H2025062545	MR21_v2.1

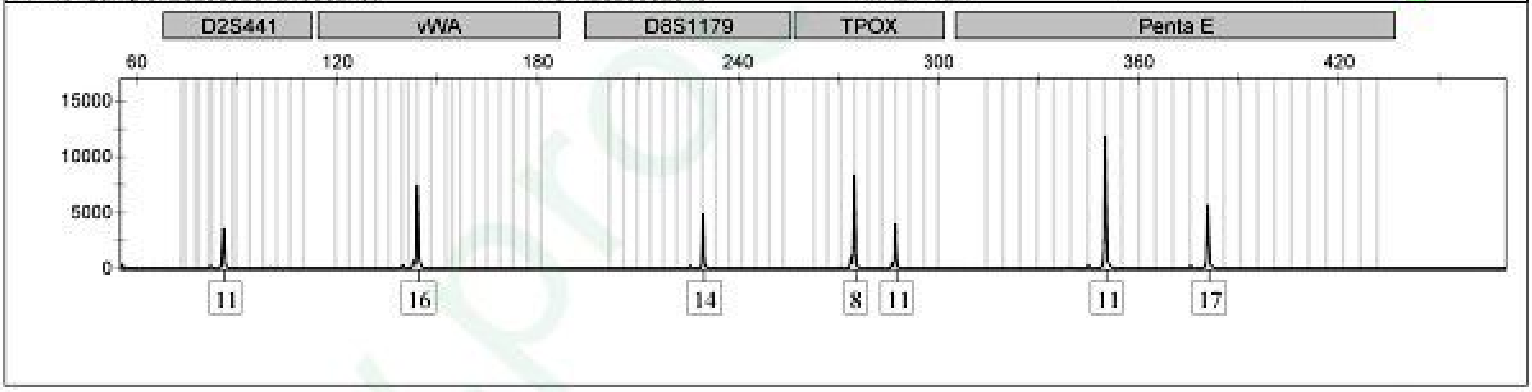

F7_45_Sample_20250625_213352.fsa	PC-H2025062545	MR21_v2.1

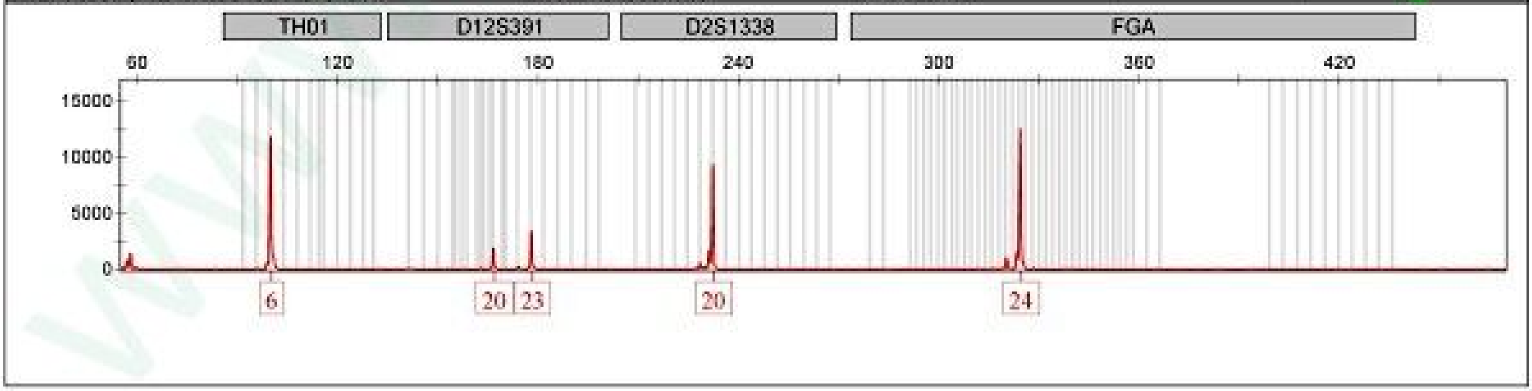

www.procell.com.cn
