# Supplementary material for: Fe‐S Protein FDX1 Triggers Tumor‐Intrinsic Innate Immunity via Mitochondrial Nucleic Acids Release to Orchestrate Ferroptosis in CCRCC
Source: Adv Sci (Weinh). 2025 Nov 7;13(6):e18323. doi: 10.1002/advs.202518323 (PMC12866870; doi:10.1002/advs.202518323)
Supplement: Supplementary file 5 — Supporting Information [file ADVS-13-e18323-s001.zip › SN12C STR RRID CVCL_1705.pdf]

STR Loci	样品名称：PC-H2025081435	数据库名称：SN12C
Amelogenin	X	X
CSF1PO	9,10	9,10
D2S1338	17,25	17,25
D3S1358	15	15
D5S818	11	11
D7S820	9	9
D8S1179	12	12
D13S317	9	9
D16S539	11	11
D18S51	12	12
D19S433	14	14
D21S11	29,30	29,30
FGA	21,22	21,22
Penta D	10,15	
Penta E	9,16	
TH01	6,8	6,8
TPOX	8,11	8,11
vWA	15	15
D6S1043	17	
D12S391	21	
D2S441	10,11	
ExPASy数据库匹配度100.00%，匹配位点数15（https://www.cellosaurus.org/index.html）		

普诺赛® | Procell system
普诺赛® | Procell system

Applied Biosystems
GeneMapper Software 6

20250814

Sample File	Sample Name	Panel	SQI	SQ
A7_35_Sample_20250814_233405.fsa	PC-H2025081435	MR21_v2.1		

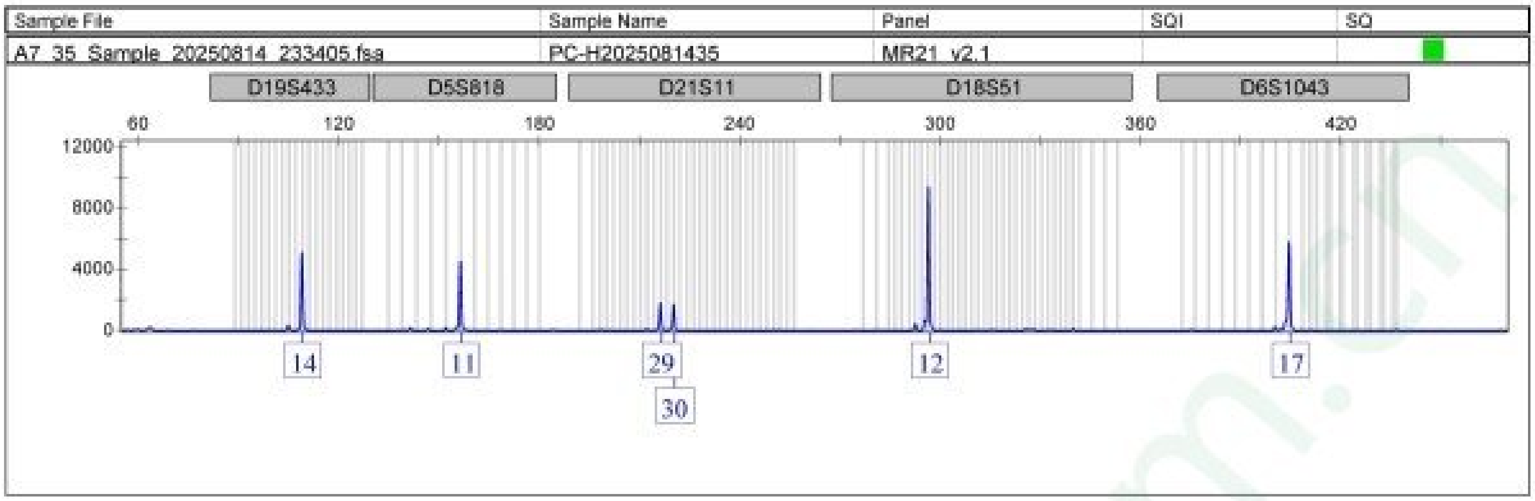

A7_35_Sample_20250814_233405.fsa	PC-H2025081435	MR21_v2.1		

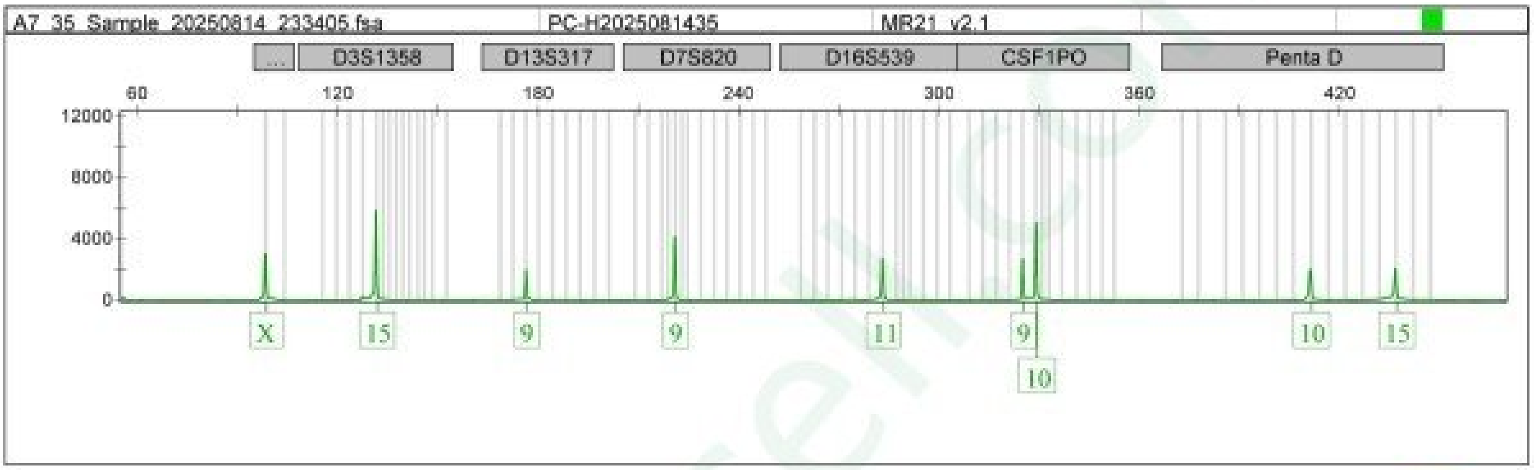

A7_35_Sample_20250814_233405.fsa	PC-H2025081435	MR21_v2.1		

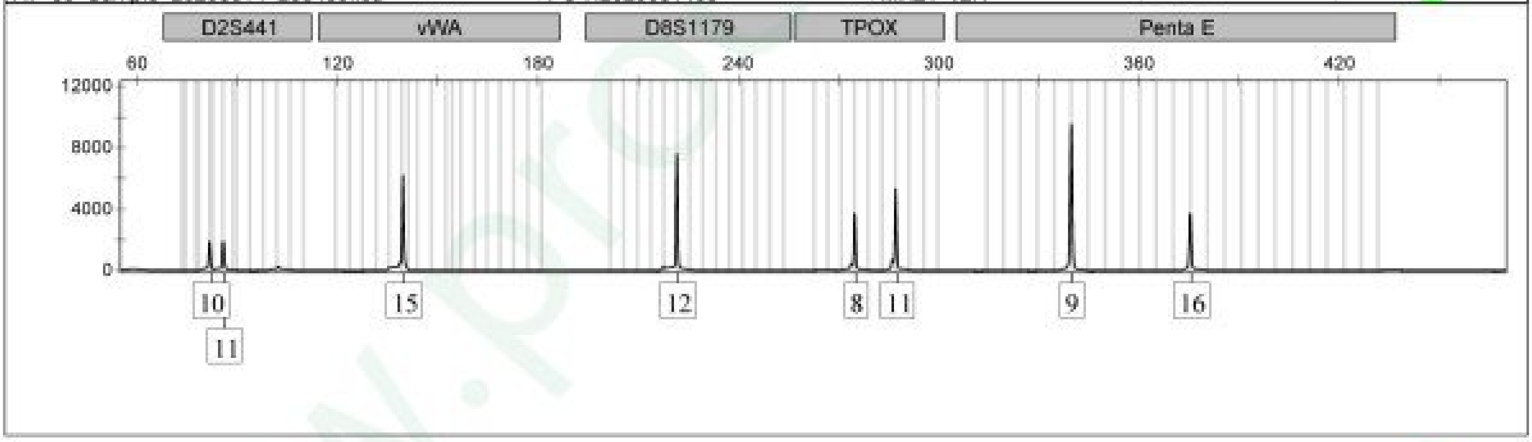

A7_35_Sample_20250814_233405.fsa	PC-H2025081435	MR21_v2.1		

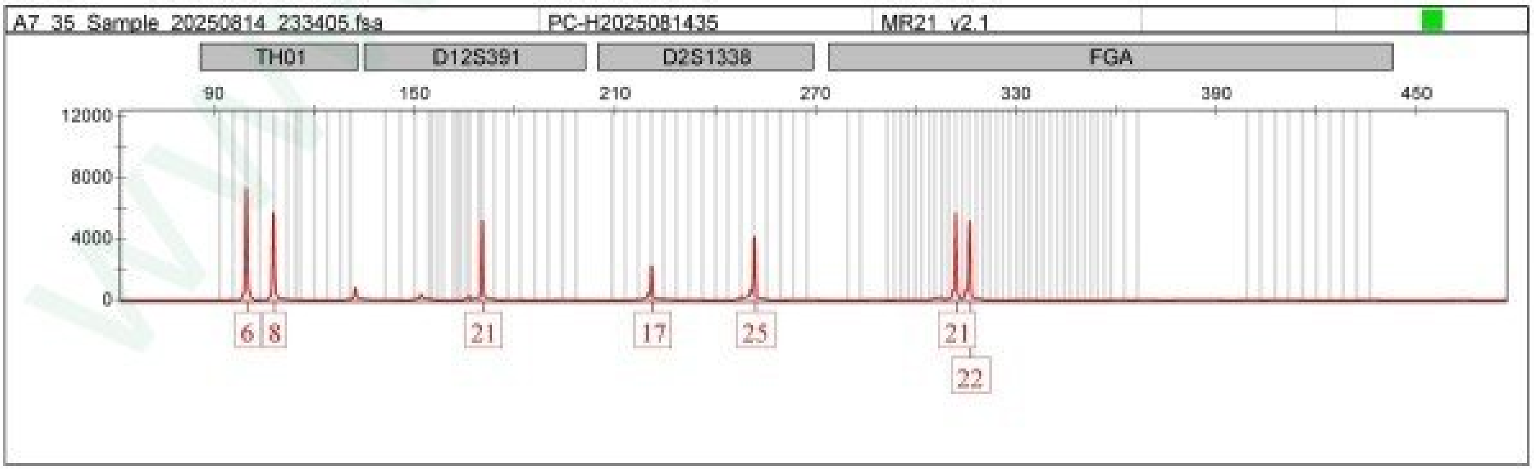

www.procell.com.cn
