# Supplementary material for: Fe‐S Protein FDX1 Triggers Tumor‐Intrinsic Innate Immunity via Mitochondrial Nucleic Acids Release to Orchestrate Ferroptosis in CCRCC
Source: Adv Sci (Weinh). 2025 Nov 7;13(6):e18323. doi: 10.1002/advs.202518323 (PMC12866870; doi:10.1002/advs.202518323)
Supplement: Supplementary file 5 — Supporting Information [file ADVS-13-e18323-s001.zip › TCMK-1 STR RRID CVCL_2772.pdf]

TCMK-1 （小鼠肾小管上皮细胞）

细胞基本信息

**产品货号**	**AW-CCM510**
**产品规格**	1×10^6cells
**包装规格**	T25培养瓶/1ml冻存管
**细胞形态**	上皮细胞样，贴壁生长
**来源**	C3H小鼠， 肾小管
**培养条件**	**MEM+10%FBS+1%P/S**<br><br>空气，95%；二氧化碳，5%<br>37℃
**细胞描述**	该细胞是1963年从C3H小鼠肾小管组织分离培养的细胞，通过SV40 转化的并且对 SV40 T 抗原呈阳性。约 2% 的细胞对 SV40 病毒抗原呈阳性，但病毒不可恢复。经检测，鼠痘病毒（鼠痘）呈阴性。 所有细胞显示 2-6 个具有次级收缩的近端着丝粒染色体和每个细胞 1-4 个双臂染色体。百分之六十的细胞有一条或多条微小的染色体
**倍增时间**	

仅供科研使用，不可用于临床诊断和治疗。

售后服务告知书

1、 收到细胞

1） 收到细胞后，活细胞首先观察培养瓶是否完好，培养液是否漏液，培养基是否浑浊；冻存细胞是否干冰已挥发完，冻存管盖是否脱落，破碎，若有这类情况，请务必拍照记录，并于收货 24h 内与我们联系。

2） 细胞处理：

复苏的细胞：如果是 T-25 培养瓶活细胞，收到后请用 75%的酒精对培养瓶表面进行消毒处理，然后转入培养箱中静置 2~3h 后再进行后续处理。

备注：运输用的培养基不宜再次用来培养细胞，请按照说明书新配置完全培养基来培养细胞。

冻存细胞：如果是干冰运输的冻存细胞，收到后请立即转入液氮存储或者短暂（24h）放置-80 度冰箱保存，或者直接进行细胞复苏。

2、 细胞出现问题，可以重发的情况有哪些？

1） 细胞运输过程中的各种问题，比如细胞丢失，培养基漏液，培养瓶破碎等，重发；

2） 细胞污染问题，请在收到细胞 **48h 内**，联系我们，并提供真实的图片及结果，核实后重发；

3） 细胞活力问题，活细胞培养 **24h**，干冰冻存发货的细胞复苏后 24h，绝大多数细胞未存活，重发；

4） **1 周内**出现问题，并提供收到细胞前 3 天细胞拍照记录，期间与销售人员沟通反馈情况的，由技术员判断为我方责任的，重发；技术人员判断为双方共同承担责任的，由双方进行协商处理或者按照合同价的 50%收费重发；

5） **1 周以后**，细胞出现问题或者污染，可以申请合同价 50%再发一瓶。

3、 细胞出现问题，不予重发的情况有哪些？

1） 客户操作不当导致细胞污染，不重发；**1 周内**可以申请合同价 50%再发一瓶；

2） 客户未按照推荐培养基培养，导致细胞状态不好，不重发；

3） 细胞状态不好，收到细胞 **3 天内**，未告知，不重发；

4） 视具体情况而定。

发表[中文论文]请标注：TCMK-1 （AW-CCM510）由艾碧维生物科技有限公司提供；
发表[英文论文]请标注：TCMK-1 （AW-CCM510）were provided by *Abiowell Biotechnology Co., Ltd.*

细胞复苏、传代及冻存流程参考

1、 细胞复苏

1） 配制完全培养基：基础培养基+胎牛血清+双抗（特殊培养基特殊配置）；

2） 细胞复苏：取 5ml 完全培养基于 15ml 离心管中，37℃水浴锅预热，从液氮管（或者-80 度冰箱）中快速取出冻存的细胞，放入 37℃水浴锅中，摇晃使快速化冻（1min 左右），然后将化冻的细胞和预热的培养基，移入超净工作台中，化冻的细胞加入到含预热培养基的 15ml 离心管中，1000rpm 离心 5min;

3） 吸弃上清，得到细胞沉淀，用 2ml 完全培养基轻轻重悬细胞，加入到 T25 培养瓶中，做好标记，放入 37℃，5%CO_2饱和适度培养箱中培养（培养皿复苏效果更好）；

4） 24h 后，观察细胞贴壁情况（未贴壁的即为死细胞--针对贴壁细胞）， 吸弃旧培养基，加入新鲜的预热（室温或 37℃）的完全培养基，继续培养。

2、 细胞传代

1） 待细胞生长到 80%-90%汇合度时， 吸弃旧的培养基，加入 1ml 无菌 PBS 润洗一次，以去除残余的培养基及血清（血清含有胰酶的抑制因子），然后加入 1ml 0.25%胰酶，37℃培养箱中消化（1~2min 左右，不同细胞消化时间不同），取出细胞，镜下观察细胞至细胞皱缩变圆;

2） 加入 1ml 完全培养基（含 FBS）终止消化，轻轻拍打，使细胞脱落下来成单个细胞悬液，收集细胞于 15ml 无菌离心管中，1000rpm，离心 5min;

3） 收集细胞沉淀，完全培养基重悬，一分为二（可根据细胞生长速度调整比例），分别加入到 2 个新的培养瓶中，做好标记，放入培养箱中培养。

3、细胞冻存

1） 按照细胞传代方法，在超净工作台内消化收集细胞沉淀，取少量细胞用于计数;

2） 用预冷的 1ml 冻存液（90%完全培养基+10%DMSO）或者无血清细胞冻存液重悬细胞，加入到 1.2ml 冻存管中，密度为 $1*10^6$ 个/ml。

3） 放入程序冻存盒，-80℃过夜后，转入液氮长期保存。

STR检 测 结 果

(一) 检验基本情况

编号	多等位基因	匹配细胞系	人源污染	与对比细胞匹配度EV值	匹配说明
	无	鼠源细胞系	无	1.0	完全匹配

- 多等位基因指三等位及以上基因现象。
- 本次检测各细胞分型结果良好。

(二) 各样本描述

- 该株细胞鉴定结果为小鼠细胞系，因数据库EXPASY未登录TCMK-1相关STR数据信息，细胞STR分型结果中未检索到匹配的细胞系，样本与对照小鼠细胞系TCMK-1基因型一致，细胞号对应CVCL_2772，STR分型结果完全匹配。本次检测在该细胞系中没有发现多等位基因，无人源污染。
- 备注：待测细胞系与收录于ATCC, DSMZ, JCRB 和 RIKEN数据库的细胞系STR数据进行比对，未收录于以上细胞库的细胞系将无法匹配。下列位点中D4S2408为人源位点，用于检测该细胞是否有人源污染。

（三）样本分型结果

细胞的STR位点和Amelogenin位点的基因分型结果

Loci	送检细胞STR信息				细胞库细胞STR信息		
	送检细胞名：Tcmk-1				细胞库细胞名：TCMK-1		
	Allele1	Allele2	Allele3	Allele4	Allele1	Allele2	Allele3
4-2	237.86【20.3】				237.35【20.3】		
5-5	335.93【14】				335.53【14】		

6-4	300.15<br>【18】	299.69<br>【18】
6-7	334.58<br>【12】	334.1<br>【12】
9-2	221.37<br>【15】	220.87<br>【15】
12-1	226<br>【16】	225.26<br>【16】
15-3	213.26<br>【25.3】	213.61<br>【25.3】
18-3	160.42<br>【18】	161.37<br>【18】
X-1	404.52<br>【25.3】	404.92<br>【25.3】
D4S2408		
